# The influence of a short-term gluten-free diet on the human gut microbiome

**DOI:** 10.1186/s13073-016-0295-y

**Published:** 2016-04-21

**Authors:** Marc Jan Bonder, Ettje F. Tigchelaar, Xianghang Cai, Gosia Trynka, Maria C. Cenit, Barbara Hrdlickova, Huanzi Zhong, Tommi Vatanen, Dirk Gevers, Cisca Wijmenga, Yang Wang, Alexandra Zhernakova

**Affiliations:** Department of Genetics, University of Groningen, University Medical Centre Groningen, Groningen, The Netherlands; Top Institute Food and Nutrition, Wageningen, The Netherlands; BGI, Shenzhen, 518083 China; Wellcome Trust Sanger Institute, Hinxton, Cambridge, CB10 1SA UK; Broad Institute of MIT and Harvard, Cambridge, MA 02142 USA; Department of Computer Science, Aalto University School of Science, Espoo, 02150 Finland

**Keywords:** Microbiome, Gluten-free diet, Biomarker, Observation study

## Abstract

**Background:**

A gluten-free diet (GFD) is the most commonly adopted special diet worldwide. It is an effective treatment for coeliac disease and is also often followed by individuals to alleviate gastrointestinal complaints. It is known there is an important link between diet and the gut microbiome, but it is largely unknown how a switch to a GFD affects the human gut microbiome.

**Methods:**

We studied changes in the gut microbiomes of 21 healthy volunteers who followed a GFD for four weeks. We collected nine stool samples from each participant: one at baseline, four during the GFD period, and four when they returned to their habitual diet (HD), making a total of 189 samples. We determined microbiome profiles using 16S rRNA sequencing and then processed the samples for taxonomic and imputed functional composition. Additionally, in all 189 samples, six gut health-related biomarkers were measured.

**Results:**

Inter-individual variation in the gut microbiota remained stable during this short-term GFD intervention. A number of taxon-specific differences were seen during the GFD: the most striking shift was seen for the family *Veillonellaceae* (class *Clostridia*), which was significantly reduced during the intervention (*p* = 2.81 × 10^−05^). Seven other taxa also showed significant changes; the majority of them are known to play a role in starch metabolism. We saw stronger differences in pathway activities: 21 predicted pathway activity scores showed significant association to the change in diet. We observed strong relations between the predicted activity of pathways and biomarker measurements.

**Conclusions:**

A GFD changes the gut microbiome composition and alters the activity of microbial pathways.

**Electronic supplementary material:**

The online version of this article (doi:10.1186/s13073-016-0295-y) contains supplementary material, which is available to authorized users.

## Background

Gluten is a major dietary component of wheat, barley, and rye. In genetically susceptible individuals, the consumption of gluten triggers the development of coeliac disease – an autoimmune disorder commonly seen in populations of European ancestry (with a frequency of approximately 1 %) [1]. In the absence of any medication, the only treatment is a life-long gluten-free diet (GFD), which is effective and well tolerated by the majority of patients. Non-coeliac gluten sensitivity, another common disorder linked to the consumption of gluten-containing food and resulting in a range of symptoms of intestinal discomfort (such as diarrhea and abdominal pain), has also been shown to improve on a GFD [2, 3]. More recently, a GFD is being considered as a way to ameliorate symptoms in patients with irritable bowel syndrome (IBS) [4].

However, beyond these medical indications, more and more individuals are starting on a GFD to improve their health and/or to control weight. The diet’s popularity has risen rapidly in the last few years, making it one of the most popular diets worldwide, along with a low-carbohydrate diet and a fat-free diet. The numbers of those adopting the diet for non-medical reasons now surpass the numbers of those who are addressing a permanent gluten-related disorder [3].

Several studies have reported the effect of a GFD on the composition of the gut microbiome in coeliac disease patients [5–7]. In these studies, the microbiome composition in coeliac patients on a GFD was compared with untreated patients and healthy individuals. The most consistent observation across these studies is the difference in the abundance and diversity of *Lactobacillus* and *Bifidobacterium* in the treated and untreated coeliac disease patients. It should be noted that these studies were relatively small (seven to 30 participants in each group). Specifically, De Palma et al. [8] assessed the effect of a one-month GFD on ten healthy individuals, but the study was limited to the use of non-sequence based methods, including FISH and qPCR. Their study described how *Bifidobacterium*, *Clostridium lituseburense*, *Faecalibacterium prausnitzii*, *Lactobacillus*, and *Bifidobacterium longum* were decreased during GFD, whereas *Escherichia coli*, *Enterobacteriaceae*, and *Bifidobacterium angulatum* were increased. To the best of our knowledge, there has been no comprehensive analysis of the effect of a GFD on the entire gut microbiome composition using a next-generation sequencing approach.

The effect of other diet interventions on the microbiome composition was recently studied using the 16S rRNA sequencing method [9]. In particular, it was shown that a short-term animal-based diet led to an increased abundance of bile-tolerant microorganisms (*Alistipes*, *Bilophila*, and *Bacteroides*) and a decreased abundance of Firmicutes, which metabolize dietary plant polysaccharides (*Roseburia*, *Eubacterium rectale*, and *Ruminococcus bromii*) [9].

In this work we assessed the effect of a GFD on gut microbiota using the next-generation 16S rRNA sequencing method. The analysis was performed in 189 samples, representing up to nine time points for 21 individuals. We investigated the diet-related changes both on the level of taxonomic units as well as on the predicted bacterial pathways. Next to this, we assessed a set of selected biomarkers to assess the gut health in relation to changes in bacterial composition and their association to a GFD. Our study offers insights into the interaction between the gut microbiota and a GFD.

## Methods

### Study design

**Fig. 1:**
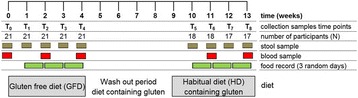
Timeline of GFD study, including number of participants and collected samples

We enrolled 21 participants (nine men and twelve women), without any known food intolerance and without known gastrointestinal disorders, in our GFD study for 13 weeks (Fig. 1). After baseline measurements (T = 0), all the participants started a GFD for four weeks (T = 1–4), followed by a “wash-out” period of five weeks. Subsequently, data were collected when they returned to their habitual diets (HD, gluten-containing) for a period of four weeks (T = 5–8) (Fig. 1). Fecal samples were collected at all time points. Blood was collected at baseline, at T = 2 and T = 4 on GFD, and at T = 6 and T = 8 on HD.

The participants were aged between 16 and 61 years (mean age, 36.3 years). Mean BMI was 24.0 and 28.6 % (*n* = 6) of participants were smokers. The majority of participants were European (*n* = 19), two participants were South American, and one was Asian. Except for one, none of the participants had taken an antibiotic treatment for the year prior to the study start. In both diet periods (GFD, HD), participants kept a detailed three-day food record. All 21 participants completed the GFD period; for 17 participants all data points were available. An overview of the participants’ characteristics can be found in Additional file 1: Figure S1.

Written consent was obtained from all participants and the study followed the sampling protocol of the LifeLines-DEEP study [10], which was approved by the ethics committee of the University Medical Centre Groningen, document no. METC UMCG LLDEEP: M12.113965.

### Gluten-free diet and dietary intake assessment

Methods to assess GFD adherence and dietary intake have been described previously by Baranska et al. [11] In short, before the start of the study, the participants were given information on gluten-containing food products by a dietician and they were instructed how to keep a three-day food record. The food records were checked for completeness and the macronutrient intake was calculated. Days on which a participant had a daily energy intake below 500 kcal or above 5000 kcal were excluded from our analysis (*n* = 2). Of 21 participants, 15 (71 %) completed the dietary assessments; three were excluded from food intake analysis because of incomplete food records. We used the paired t-test to compare group means between GFD and HD.

### Blood sample collection

Participants’ blood samples were collected after an overnight fast by a trained physician assistant. We collected two EDTA tubes of whole blood at baseline (T0) and during the GFD period at time points T2 and T4; during the HD period one EDTA tube was collected at time points T6 and T8. Plasma was extracted from the whole blood within 8 h of collection and stored at −80 °C for later analysis.

### Microbiome analysis

#### Fecal sample collection

Fecal samples were collected at home and immediately stored at −20 °C. At the end of the 13-week study period, all samples were stored at −80 °C. Aliquots were made and DNA was isolated with the QIAamp DNA Stool Mini Kit. Isolated DNA was sequenced at the Beijing Genomics Institute (BGI).

#### Sequencing

We used 454 pyrosequencing to determine the bacterial composition of the fecal samples. Hyper-variable region V3 to V4 was selected using forward primer F515 (GTGCCAGCMGCCGCGG) and reverse primer: “E. coli 907-924” (CCGTCAATTCMTTTRAGT) to examine the bacterial composition.

We used QIIME [12], v1.7.0, to process the raw data files from the sequencer. The raw data files, sff files, were processed with the defaults of QIIME v1.7.0, however we did not trim the primers. Six out of 161 samples had fewer than 3000 reads and were excluded from the analysis. The average number of reads was 5862, with a maximum of 12,000 reads.

#### OTU picking

The operational taxonomic unit (OTU) formation was performed using the QIIME reference optimal picking, which uses UCLUST [13], version 1.2.22q, to perform the clustering. As a reference database, we used a primer-specific version of the full GreenGenes 13.5 database [14].

Using TaxMan [15], we created the primer-specific reference database, containing only reference entries that matched our selected primers. During this process we restricted the mismatches of the probes to the references to a maximum of 25 %. The 16S regions that were captured by our primers, including the primer sequences, were extracted from the full 16S sequences. For each of the reference clusters, we determined the overlapping part of the taxonomy of each of the reference reads in the clusters and used this overlapping part as the taxonomic label for the cluster. This is similar to the processes described in other studies [9, 15–18].

OTUs had to be supported by at least 100 reads and had to be identified in two samples; less abundant OTUs were excluded from the analysis.

#### Estimation of gene abundance and pathway activity

After filtering the OTUs, we used PICRUSt [19] to estimate the gene abundance and the PICRUSt output was then used in HUMAnN [20] to calculate the bacterial pathway activity. First, the reference database was clustered based on 97 % similarity to the reference sequence to better reflect the normal GreenGenes 97 % database required for PICRUSt. Three out of 1166 OTUs did not contain a representative sequence in the GreenGenes 97 % set and were therefore excluded from the analysis. Since merging the reference database at 97 % similarity level led to merging of previously different clusters, for the pathway analysis we chose to permute the cluster representative names in the OTU-table 25 times; this was to be sure that our OTU picking strategy would not cause any problems in estimating the genes present in each micro-organism. Next, we ran PICRUSt on the 25 permuted tables and calculated the average gene abundance per sample. The average correlations between the permutations within a sample was higher than 0.97 (Pearson r). Hence, we averaged the PICRUSt output, which was then used to calculate the pathway activity in HUMAnN.

#### Changes in the gut microbiome or in gene abundance due to diet

To identify differentially abundant taxa, microbial biomarkers, and differences in pathway activity between the GFD and HD periods, we used QIIME and MaAsLin [21]. QIIME was used for the alpha-diversity analysis, principal coordinate analysis (PCoA) over unifrac distances, and visualization. In the MaAsLin analysis we corrected for ethnicity (defined as continent of birth) and gender. MaAsLin was used to search for differentially abundant taxonomic units to discriminate between the GFD and HD time points. Additionally, we tested for during transition from HD to GFD (T0–T4). MaAsLin uses a boosted, additive, general linear model to discriminate between groups of data.

In the MaAsLin analysis we did not test individual OTUs, but focused on the most detailed taxonomic label each OTU represented. Using the QIIMETOMAASLIN [22] tool, we aggregated the OTUs if the taxonomic label was identical and, if multiple OTUs represented a higher order taxa, we added this higher order taxa to the analysis. In this process, we went from 1166 OTUs to 114 separate taxonomic units that were included in our analysis. Using the same tool, QIIMETOMAASLIN, we normalized the microbial abundance using acrsin square root transformation. This transformation leads to the percentages being normally distributed.

In all our analyses we used the Q-value calculated using the R [23] Q-value package [24] to correct for multiple testing. The Q-value is the minimal false discovery rate at which a test may be called significant. We used a Q-value of 0.05 as a cutoff in our analyses.

### Biomarkers

Six biomarkers related to gut health were measured in the “Dr. Stein & Colleagues” medical laboratory (Maastricht, the Netherlands). These biomarkers included: fecal calprotectin and a set of plasma cytokines as markers for the immune system activation [25–27]; fecal human-β-defensin-2 as a marker for defense against invading microbes [28, 29]; fecal chromogranin A as a marker for neuro-endocrine system activation [30–32]; fecal short-chain fatty acids (SCFA) secretion as a marker for colonic metabolism [33]; and plasma citrulline as a measure for enterocyte mass [34, 35]. The plasma citrulline level and the panel of cytokines (IL-1β, IL-6, IL-8, IL-10, IL-12, and TNFα) were measured by high-performance liquid chromatography (HPLC) and electro-chemiluminescence immunoassay (ECLIA), respectively. In feces, we measured calprotectin and human-β-defensin-2 levels by enzyme-linked immunosorbent assay (ELISA), chromogranin A level by radioimmunoassay (RIA), and the short-chain fatty acids acetate, propionate, butyrate, valerate, and caproate by gas chromatography–mass spectrometry (GC-MS). All biomarker analyses were performed non-parametrically, with tie handling, because of the high number of samples with biomarker levels below the detection limit. We used the Wilcoxon test to compare the average biomarker levels between the diet periods and the Spearman correlation to search for relations between the microbiome or gene activity data and the biomarker levels.

## Results

### Food intake

We first investigated if a GFD had a significant effect on the daily intake of macronutrients by analyzing the GFD and HD food records from participants (Additional file 2: Table S1). Mean (SD) daily intakes of energy, protein, fat, and carbohydrate during GFD and HD are shown in Table 1. We observed slightly higher carbohydrate intake and a slightly lower fat intake on GFD; however, none of the differences in energy or macronutrient intake were significantly different. We therefore concluded that dietary macronutrient composition was not significantly changed by following a GFD.

**Table 1:** Mean and standard deviation (SD) of energy, protein, carbohydrates, and fat intake during the gluten-free diet (GFD) and habitual diet (HD). g = grams, en% = energy %

	GFD (*n* = 12)		HD (*n* = 12)		
Nutrient	Mean	SD	Mean	SD	*p* value
Energy (kcal)	1709.5	344.0	1811.5	433.9	0.243
Protein (g)	73.1	18.4	78.1	18.2	0.401
Protein (en%)	17.1		17.2		
Carbohydrates (g)	211.1	50.3	199.9	63.2	0.275
Carbohydrates (en%)	49.4		44.1		
Fat (g)	63.7	18.1	72.5	24.3	0.109
Fat (en%)	33.6		36.0		

### Microbial differences due to diet

In total we used 155 fecal samples, originating from 21 individuals, for the microbiota analysis and we observed 114 different taxonomic units. We first checked if GFD influenced the number and proportion of bacteria in individual participants, for which we investigated differences in alpha diversity between the GFD and HD time points using several alpha diversity measures (Observed species, Shannon, Chao1, and Simpson indexes). We found no differences in the alpha diversity in any of these tests. Therefore, we concluded that a change in diet did not influence the bacterial diversity within a sample.

Next, we tested if there was any difference in the bacterial diversity related to variation in diet between participants (beta-diversity) by comparing the unweighted unifrac distance in sample groups. We observed a strong difference when comparing different time points from a single individual to all other individuals, regardless of diet type, Wilcoxon *p* value <2.2 × 10^−16^. When we compared the diet-induced differences within the same individual, we saw a small but significant change, Wilcoxon *p* value = 0.024, although the same diet time points were slightly more alike (Additional file 3: Figure S2).

**Fig. 2:**
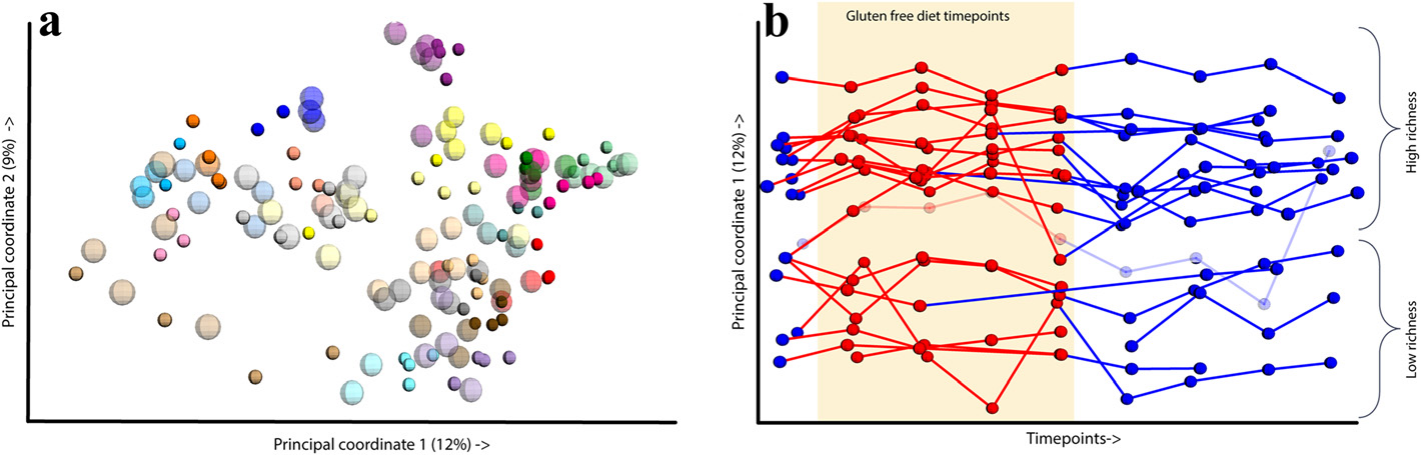
PCoA plot showing the differences in the samples. **a** Samples plotted on PCoA 1 and 2, percentage of explained variation is given in the legends. Each color represents an individual, the *larger and less opaque spheres* are gluten-free diet time points, and the *smaller spheres* in the same color are habitual diet time points. **b** The differences in the first component over the time points. There are two groups based on richness, i.e. high versus low, one individual had samples in both groups. The sample belonging to both richness groups has a bolder color

In the PCoA analysis over the unweighted unifrac distance (Fig. 2a), we also saw that the main driver of the diversity is the inter-individual difference, with participants clustering together, both during and after the dietary intervention. In the first ten principal coordinates, which explain more than half of the total variation, we observed changes between the time points for individual participants, although there was no single component, or combination of components, capturing the difference between the GFD versus HD time points in the first ten components.

We therefore concluded that a GFD has a significant effect on the diversity between the groups, but that the inter-individual effect on the variation of the microbiome is stronger than the effect of diet.

We further investigated changes in beta-diversity in relation to the time points (Fig. 2b). When we plotted PCo1 versus the time points, we observed a separation into two groups. Since PCo1 describes the difference in alpha-diversity between samples, we concluded that this separation is based on richness. The richness separates all but one participant into either a clear high-richness or low-richness group (Fig. 2b). There is a significant difference in richness between the two groups, Wilcoxon *p* value = 0.0016, excluding the one participant who seems to be an intermediate. However, unlike the study by Le Chatelier et al. [36], we did not see any significant difference in stability, i.e. in variation in richness, between the low- and high-richness groups.

#### Differentially abundant taxa

**Fig. 3:**
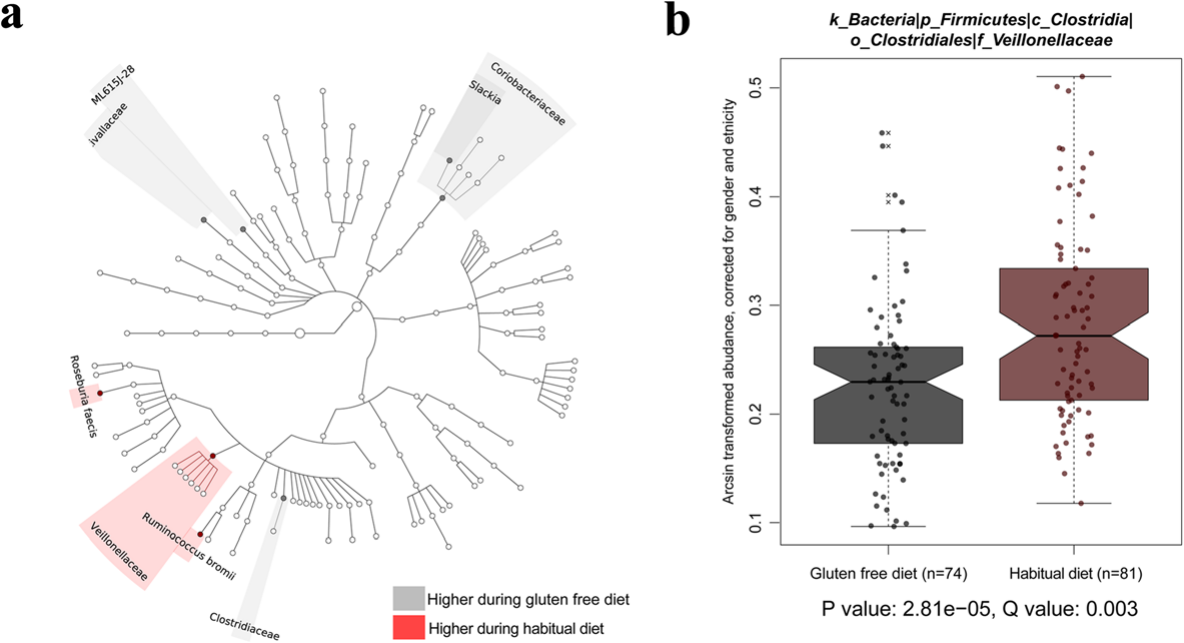
**a** Cladogram showing the differentially abundant taxa. This plot shows the different levels of taxonomy. *Gray* indicates bacteria higher in the habitual diet and *red* indicates those higher in the gluten-free diet. The different *circles* represent the different taxonomic levels. (From inside to outside: Kingdom, Phylum, Class, Order, Family, Genus, and Species). **b** Comparison of the abundance of Veillonellaceae* in the gluten-free diet vs. habitual diet. In the plot, the aggregate “overall weeks” including correction is shown. * Veillonellaceae is placed in the order Clostridiales in GreenGenes 13.5. However, according to the NCBI classification, it belongs to order Negativicutes

When comparing the HD and GFD time points, corrected for age and ethnicity in MaAsLin, we observed eight significant microbial changes (Fig. 3 and Table 2). The strongest association was found to the family *Veillonellaceae*, of which the abundance in the gut dropped significantly on a GFD (*p* = 2.81 × 10^−05^, q = 0.003) (Fig. 3b and Additional file 4: Figure S3). Other species that decreased on a GFD included *Ruminococcus bromii* (*p* = 0.0003, q = 0.01) and *Roseburia faecis* (*p* = 0.002, q = 0.03). While families *Victivallaceae* (*p* = 0.0002, q = 0.01), *Clostridiaceae* (*p* = 0.0006, q = 0.015), and *Coriobacteriaceae* (*p* = 0.003, q = 0.035), order *ML615J-28* (*p* = 0.001, q = 0.027), and genus *Slackia* (*p* = 0.002, q = 0.01) increased in abundance on a GFD.

**Table 2:** GFD-induced changes in taxonomic composition

Taxonomic unit	Coefficient	N.not.0/N	*p* value	Q-value
p_Firmicutes|c_Clostridia|o_Clostridiales|f_Veillonellaceae^a^	0.0424	155/155	2.81 × 10^−5^	0.0030
p_Lentisphaerae|c_Lentisphaeria|o_Victivallales|f_Victivallaceae	−0.0093	89/155	2.30 × 10^−4^	0.0105
p_Firmicutes|c_Clostridia|o_Clostridiales|f_Ruminococcaceae|g_Ruminococcus|s_bromii	0.0151	99/155	2.94 × 10^−4^	0.0105
p_Firmicutes|c_Clostridia|o_Clostridiales|f_Clostridiaceae	−0.0121	150/155	5.69 × 10^−4^	0.0152
p_Tenericutes|c_RF3|o_ML615J-28	−0.0095	82/155	1.30 × 10^−3^	0.0277
p_Firmicutes|c_Clostridia|o_Clostridiales|f_Lachnospiraceae|g_Roseburia|s_faecis	0.0065	100/155	1.88 × 10^−3^	0.0326
p_Actinobacteria|c_Coriobacteriia|o_Coriobacteriales|f_Coriobacteriaceae|g_Slackia	−0.0044	43/155	2.14 × 10^−3^	0.0326
p_Actinobacteria|c_Coriobacteriia|o_Coriobacteriales|f_Coriobacteriaceae	−0.0137	155/155	2.67 × 10^−3^	0.0357

A positive coefficient means more of the microbe was present during the habitual diet, while a negative coefficient means less of the microbe was present during the habitual diet. All associations were to the kingdom bacteria, for readability the kingdom label is not presented. ^a^ *Veillonellaceae* is placed in the order Clostridiales in GreenGenes 13.5. However, according to the NCBI classification, it belongs to order Negativicutes

Next, we tested for trends during the diet change; however, we did not observe a time-dependent change in the microbiome composition. Since we observed two different groups based on richness in the PCoA analysis, we tested for different reactions to the change in diet in the high-richness- and low-richness groups. However, no significant associations were found in this analysis.

Since six out of the 28 participants smoked, we tested for overlap between smoke-associated bacteria and diet-related bacteria. We did not find any overlap; Additional file 5: Table S2 shows the bacteria associated with smoking.

### Imputation of bacterial function

Next to the taxonomic associations, we also aimed to study differences in pathway composition in relation to GFD. We applied PICRUSt and HUMAnN for pathway annotation, as described in Methods. In total, 161 pathways and 100 modules were predicted, all of the pathways and modules were found in at least 1 % of the samples.

**Fig. 4:**
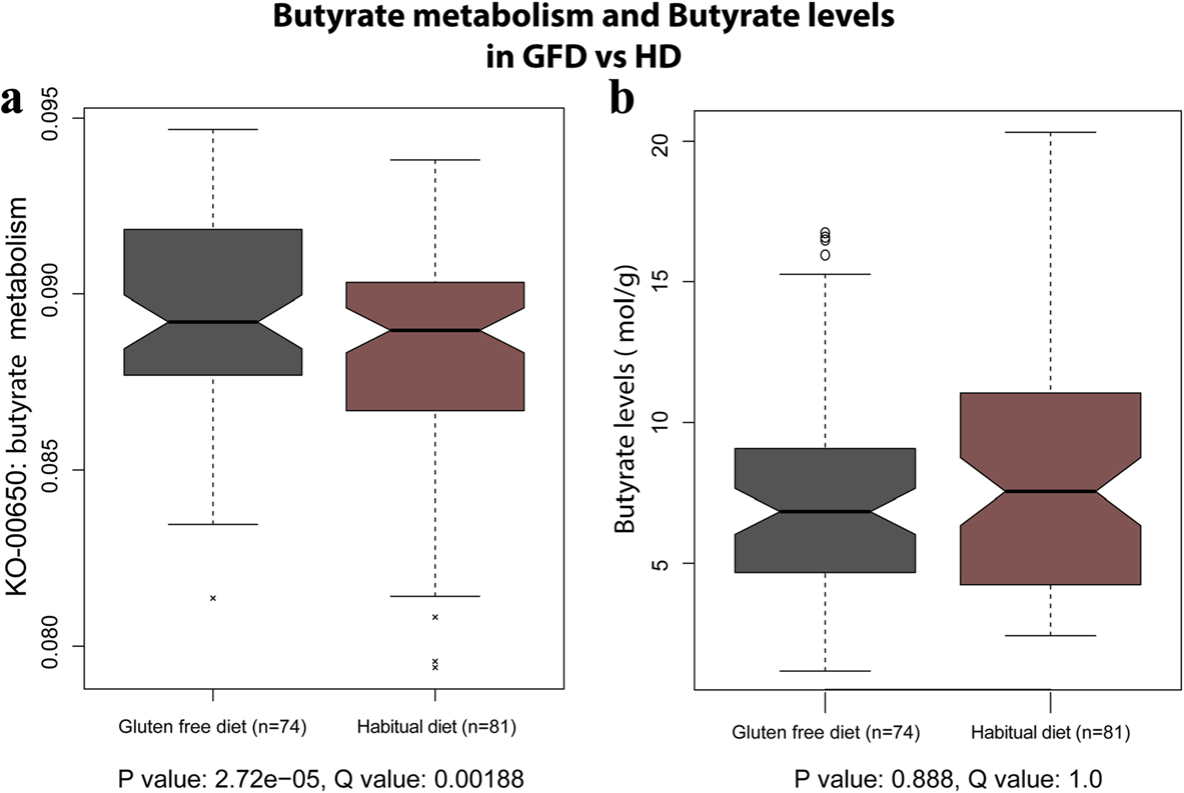
*Box plot* of predicted activity of butyrate metabolism per diet period (**a**) and the butyrate levels (mol/g) per diet period (**b**). There was a significant increase in activity in butyrate metabolism (q = 0.001877), but no change in butyrate level was observed

We used MaAsLin to identify differences in the pathway composition and conducted the same tests – GFD versus HD and the time-series test – as for the microbial composition. The data were again corrected for age and ethnicity. We observed that 19 KEGG pathways and two KEGG modules (Table 3) were different in abundance between GFD and HD. We did not observe associations related to the transition from GFD to HD (T0–T4). Four out of five top associations, all with a Q-value <0.0003, are related to metabolism changes: tryptophan metabolism, butyrate metabolism (Fig. 4a), fatty acid metabolism, and seleno-compound metabolism.

**Table 3:** GFD-induced changes in pathway and module activity

Feature	Coefficient	N.not.0/N	*p* value	Q-value
KO00380: Tryptophan metabolism	−0.0011	155/155	2.45 × 10^−5^	0.002
KO00650: Butyrate metabolism	−0.0014	155/155	2.72 × 10^−5^	0.002
KO00071: Fatty acid metabolism	−0.0011	155/155	4.74 × 10^−5^	0.002
KO00450: Selenocompound metabolism	0.0009	155/155	9.23 × 10^−5^	0.003
KO00630: Glyoxylate and dicarboxylate metabolism	−0.0010	155/155	2.53 × 10^−4^	0.007
KO00520 Amino sugar and nucleotide sugar metabolism	0.0009	155/155	2.83 × 10^−4^	0.007
M00064: ADP-L-glycero-D-manno-heptose biosynthesis	0.0066	155/155	4.12 × 10^−4^	0.023
KO00643: Styrene degradation	−0.0013	155/155	4.29 × 10^−4^	0.008
M00077: Chondroitin sulphate degradation Chondroitin sulphate degradation	−0.0037	76/155	5.81 × 10^−4^	0.023
KO00760: Nicotinate and nicotinamide metabolism	0.0008	155/155	6.79 × 10^−4^	0.012
KO00620: Pyruvate metabolism	−0.0012	155/155	0.002	0.023
KO00253: Tetracycline biosynthesis	−0.0027	155/155	0.002	0.024
KO00471: D-Glutamine and D-glutamate metabolism	0.0012	155/155	0.002	0.024
KO04122: Sulphur relay system	−0.0020	155/155	0.002	0.024
KO00633: Nitrotoluene degradation	−0.0022	155/155	0.002	0.024
KO00072: Synthesis and degradation of ketone bodies	−0.0020	155/155	0.003	0.028
KO00310: Lysine degradation	−0.0007	155/155	0.003	0.031
KO00624: Polycyclic aromatic hydrocarbon degradation	0.0006	155/155	0.005	0.043
KO00561: Glycerolipid metabolism	−0.0012	155/155	0.005	0.043
KO00680: Methane metabolism	−0.0006	155/155	0.006	0.047
KO00550: Peptidoglycan biosynthesis	0.0011	155/155	0.007	0.047

A positive coefficient means more activity of the pathway/module during the habitual diet, while a negative coefficient means less activity of the pathway/module during the habitual diet

### Biomarkers in relation to diet changes

#### Biomarkers related to GFD versus HD

We measured four biomarkers in feces: calprotectin, human-β-defensin-2, chromogranin A, and a set of five short-chain fatty acids (acetate, propionate, butyrate, valerate, and caproate). In addition, we measured citrulline levels and a panel of cytokines (IL-1β, IL-6, IL-8, IL-10, IL-12, and TNFα) in blood. The Wilcoxon test was used to test biomarker level differences between the average values and the GFD and HD period values. We saw no significant change in biomarker levels in relation to GFD (Table 4A and B).

**Table 4:** Median and 25 %/75 % quantiles of the measured biomarkers

	Habitual diet	Gluten-free diet	Wilcoxon test *p* value
A) Plasma			
Citrullin (mol/L)	45.60 (38.15–51.50)	48.00 (36.35–56.85)	0.9328
IL 1 Beta (g/L)	1.60 (0.68–2.10)	1.23 (0.79–1.68)	0.8870
IL 6 (g/L)	BDL (BDL–1.60)	BDL (BDL–0.38)	0.1240
IL 8 (g/L)	6.04 (2.89–12.61)	5.41 (3.34–11.19)	0.9030
IL 10 (g/L)	0.83 (0.74–1.01)	0.83 (0.74–0.97)	0.9322
IL 12P70 (g/L)	1.53 (0.95–1.78)	1.53 (0.95–2.11)	0.2131
TNF Alpha (g/L)	0.56 (BDL–4.33)	BDL (BDL–5.13)	0.9761
B) Feces			
Chromogranin A (nmol/g)	10.85 (7.69–23.09)	11.44 (7.37–27.18)	0.8128
Beta Defensin 2 (ng/g)	24.90 (18.78–35.03)	26.10 (20.03–46.90)	0.5256
Calprotectin (g/g)	21.55 (BDL–42.88)	13.05 (BDL–31.28)	0.0528
Acetate (mol/g)	24.37 (17.35–34.34)	23.61 (18.58–35.12)	0.8651
Propionate (mol/g)	7.55 (4.24–10.98)	6.84 (4.67–9.07)	0.6986
Butyrate (mol/g)	6.86 (3.53–10.63)	6.48 (4.27–10.40)	0.8882
Valerat (mol/g)	1.09 (0.74–1.76)	1.24 (0.79–1.70)	0.6824
Caproat (mol/g)	0.28 (0.05–0.85)	0.21 (0.04–0.66)	0.2488

None of the differences were statistically significant. BDL = below detection limit

#### Correlations between fecal biomarkers and microbiome

We correlated the fecal biomarker levels to the microbiome composition as well as to the microbiome predicted pathways and modules. After multiple testing correction, we observed many statistically significant correlations between the levels of biomarkers and microbiome/pathway abundances; the absolute correlation, Spearman Rho, was between 0.14 and 0.59. An expected observation was the correlation of the butyrate pathway activity to the butyrate biomarker, as we had previously observed a significant correlation between the predicted butyrate pathway activity and diet change (Table 3). When correlating the actual butyrate measurements with the predicted activity of the butyrate metabolism, we observed a low but significant correlation of −0.269 (*p* = 0.0009, q = 0.0012, Additional file 6: Figure S4). However, there was no significant difference in butyrate levels in the two diet periods (Fig. 4b and Table 4). Another interesting correlation was found between the predicted pyruvate metabolism pathway and the levels of propionate (mol/g), since propionate can be oxidized to pyruvate [37], for which we observed a correlation of −0.54 (*p* = 9.44 × 10^–13^, q = 1.48 × 10^–10^, Additional file 7: Figure S5). A complete list of the significant correlations between the fecal biomarkers and the microbiome compositions, the predicted KEGG pathway activity scores, and predicted activity of KEGG modules can be found in Additional file 8: Tables S3, Additional file 9: Table S4, and Additional file 10: Table S5.

## Discussion

We investigated the role of a four-week GFD on microbiome composition in healthy individuals and identified moderate but significant changes in their microbiome compositions and even stronger effects on the imputed activity levels of bacterial pathways.

On a taxonomic level we identified eight bacteria that change significantly in abundance on GFD: *Veillonellaceae*, *Ruminococcus bromii*, and *Roseburia faecis* decreased on GFD, and *Victivallaceae*, *Clostridiaceae*, *ML615J-28*, *Slackia*, and *Coriobacteriaceae* increased on GFD. The strongest effect was seen in the decrease of *Veillonellaceae* during GFD, Gram-negative bacteria known for lactate fermentation. This is the first time that the *Veillonellaceae* family has been associated to a dietary intervention, but it was recently shown to be decreased in autistic patients [38]. Remarkably, the patients in that study were more often on a GFD (9/10) than the control group (5/10). Our findings suggest that GFD, rather than autism, can be the cause of a lower abundance of *Veillonellaceae* in these patients, thus highlighting the importance of including dietary information in analyses of microbiota in relation to diseases. *Veillonellaceae* is considered to be a pro-inflammatory family of bacteria; an increase in *Veillonellaceae* abundance was consistently reported in IBD, IBS, and cirrhosis patients [39–41]. It is conceivable that a decrease in *Veillonellaceae* abundance might be one of the mediators of the GFD’s beneficial effect observed in patients with IBS and gluten-related disorders.

Several of the associated bacteria have been previously linked to diet changes and starch metabolism. In particular, *Ruminococcus bromii* is important for the degradation of resistant starch in the human colon [42] and is increased when on a resistant starch diet [43]. It is also known that degradation of cellulose by *Ruminococcus* results in the production of SCFA and hydrogen gas [44]; a decrease in abundance of *Ruminococcus* and its fermentation products might explain the beneficial effect of a GFD that is experienced by some IBS patients as previously reported by Aziz et al. [45]. Both *Ruminococcus bromii* and *Roseburia faecis* were recently reported to be influenced by switching from a vegetarian to a meat-containing diet [9]. It is likely that changes in these bacteria observed in relation to GFD are the consequences of the different starch composition of a GFD versus HD. Moreover, stool consistency could influence the results of microbiome composition [46]; unfortunately, data on stool composition were not collected in our study.

The five bacteria for which we found an increased abundance on GFD are less well characterized although the *Slackia* genus, its family *Coriobacteriaceae*, and the family *Clostridiaceae* have been previously linked to gastrointestinal diseases in humans – inflammatory bowel disease, celiac disease, and colorectal cancer [47–49]. The *Victivallaceae* family and ML615J-28 order have not been previously associated to diet change or phenotypic change in human. However, in general, it could be hypothesized that these bacteria benefit from a change in available substrates as a result from the change in diet, which could in turn result in altered metabolite production and related gastrointestinal complaints.

In this study we found a stronger effect of diet on the imputed KEGG pathways than on the taxonomic level. So, although the changes in the overall microbiome were moderate, there were more profound effects on the pathway activities of the microbiome.

The strength of our study lies in our analysis of the microbiome at multiple time points for the same individuals. We identified that the inter-individual variability is the strongest determinant of sample variability, suggesting that in healthy individuals the gut microbiome is stable, even with short-term changes in the habitual diet. We did not observe differences in the downstream effect of GFD in relation to high or low richness, which contradicts previous observations [50]. The study by David et al. [9] identified a profound effect of short-term diet change from a vegetarian to an animal-based diet and vice versa. This profound short-term diet effect was not observed in our study when changing from a gluten-containing to a gluten-free diet. Induced by the diet change, David et al. [9] found significant differences in macronutrient intake between meat-based and plant-based diet, whereas macronutrient intake in this study was not changed during the diets. These results suggest that changing the main energy source (meat vs. plant) has a more profound effect on the microbiome than changing the carbohydrate source (gluten). Although De Palma et al. [8] did observe a reduction in polysaccharide intake for GFD in healthy individuals, we were unable to reproduce their finding because we could not distinguish between different classes of carbohydrates in our dataset as the food composition data on GFD foods lacked this information. Further, it is possible that changes in nutritional intake other than those driven by gluten exclusion might influence microbiome changes.

For our selection of blood and stool biomarkers, we observed no significant associations with the diet change. All the selected biomarkers are markers of inflammation or metabolic changes and remained in the normal range in all our participants, with a high proportion of the values of blood inflammatory markers being below the detection limit. Overall, we conclude that a GFD and its downstream effects on the microbiome do not cause major inflammatory or metabolic changes in gut function in healthy participants. However, the lower abundance of *Veillonellaceae*, the pro-inflammatory bacterium linked to Crohn’s disease and other gut disease phenotypes, suggests a reduction in gut inflammatory state. This change in bacterial composition might be linked with a beneficial effect of GFD for patients with gut disorders such as gluten-related disorders and/or IBS.

## Conclusions

We have identified eight taxa and 21 bacterial pathways associated with a change from a habitual diet to a GFD in healthy individuals. We conclude that the effect of gluten intake on the microbiota is less pronounced than that seen for a shift from a meat-based diet to a vegetarian diet (or vice versa). However, a GFD diet clearly influences the abundance of several species, in particular those involved specifically in carbohydrate and starch metabolism. Our study illustrates that variations in diet could confound the results of microbiome analysis in relation to disease phenotypes, so dietary variations should be carefully considered and reported in such studies. The short-term GFD did not influence the levels of inflammatory gut biomarkers in healthy individuals. Further research is needed to assess the impact of a GFD on inflammatory and metabolic changes in gut function in individuals with gastrointestinal conditions such as IBS and gluten-related disorders.

### Ethics approval and consent to participate

This GFD study followed the sampling protocol of the LifeLines-DEEP study, which was approved by the ethics committee of the University Medical Centre Groningen and conform the Declaration of Helsinki, document no. METC UMCG LLDEEP: M12.113965. All participants signed their informed consent prior to study enrolment.

### Availability of data and materials

The supporting data are available to researchers in the European Nucleotide Archive, under study accession number PRJEB13219 (http://www.ebi.ac.uk/ena/data/view/PRJEB13219).

## Additional files

Additional file 1: Figure S1. Baseline characteristics of the GFD study group. (TIF 1068 kb) Additional file 2: Table S1. Results macronutrient intake per participant. (XLSX 13 kb) Additional file 3: Figure S2. Unweighted unifrac distances when comparing inter-individual vs intra individual distances. In group 1 the intra-individual differences are shown regardless of diet. Group 2 shows the intra-sample differences are shown within the same diet. Group 3 shows the intra-individual differences are shown between the two diet groups. In group 4 the inter-individual differences are shown regardless of diet. Group 5 shows the inter-sample differences are shown within the same diet. Group 6 shows the inter-individual differences are shown between the two diet groups. The main difference is the intra- vs. inter-individual difference. Also the same diet points in the samples are slightly closer to each other. However, we do not see such a phenomenon for group 5 vs. group 6. (TIF 1862 kb) Additional file 4: Figure S3. Abundance of Veillonellaceae family in the GFD participants. In all but four participants we see a clear trend of higher levels of Veillonellaceae on the habitual diet. The rightmost samples do not show this phenomenon. (TIF 4036 kb) Additional file 5: Table S2. Relation of smoking and microbiome composition. (XLSX 9 kb) Additional file 6: Figure S4. Measured butyrate levels vs. the predicted activity of butyrate metabolism. (TIF 6 kb) Additional file 7: Figure S5. Measured propionate levels vs. the predicted activity of pyruvate metabolism. (TIF 646 kb) Additional file 8: Table S3. Correlation of bacteria and levels of fecal biomarkers. (PDF 139 kb) Additional file 9: Table S4. Correlation of predicted HUMAnN pathway activity and levels of fecal biomarkers. (PDF 228 kb) Additional file 10: Table S5. Correlation of predicted HUMAnN module activity and levels of fecal biomarkers. (PDF 143 kb)

## Abbreviations

BGI: Beijing Genomics Institute
ECLIA: electro-chemiluminescence immunoassay
EDTA: ethylenediaminetetraacetic acid
ELISA: enzyme-linked immunosorbent essay
FISH: fluorescence in situ hybridization
GC-MS: gas chromatography–mass spectrometry
GFD: gluten-free diet
HD: habitual diet
HPLC: high performance liquid chromatography
IBS: irritable bowel syndrome
KEGG: Kyoto encyclopedia of genes and genomes
OTU: operational taxonomic unit
PCoA: principal coordinate analysis
qPCR: quantitative real-time polymerase chain reaction
RIA: radioimmunoassay
SCFA: short chain fatty acids
SD: standard deviation
