# Supplementary figures and images for: The influence of a short-term gluten-free diet on the human gut microbiome
Source: Genome Med. 2016 Apr 21;8:45. doi: 10.1186/s13073-016-0295-y (PMC4841035; doi:10.1186/s13073-016-0295-y)

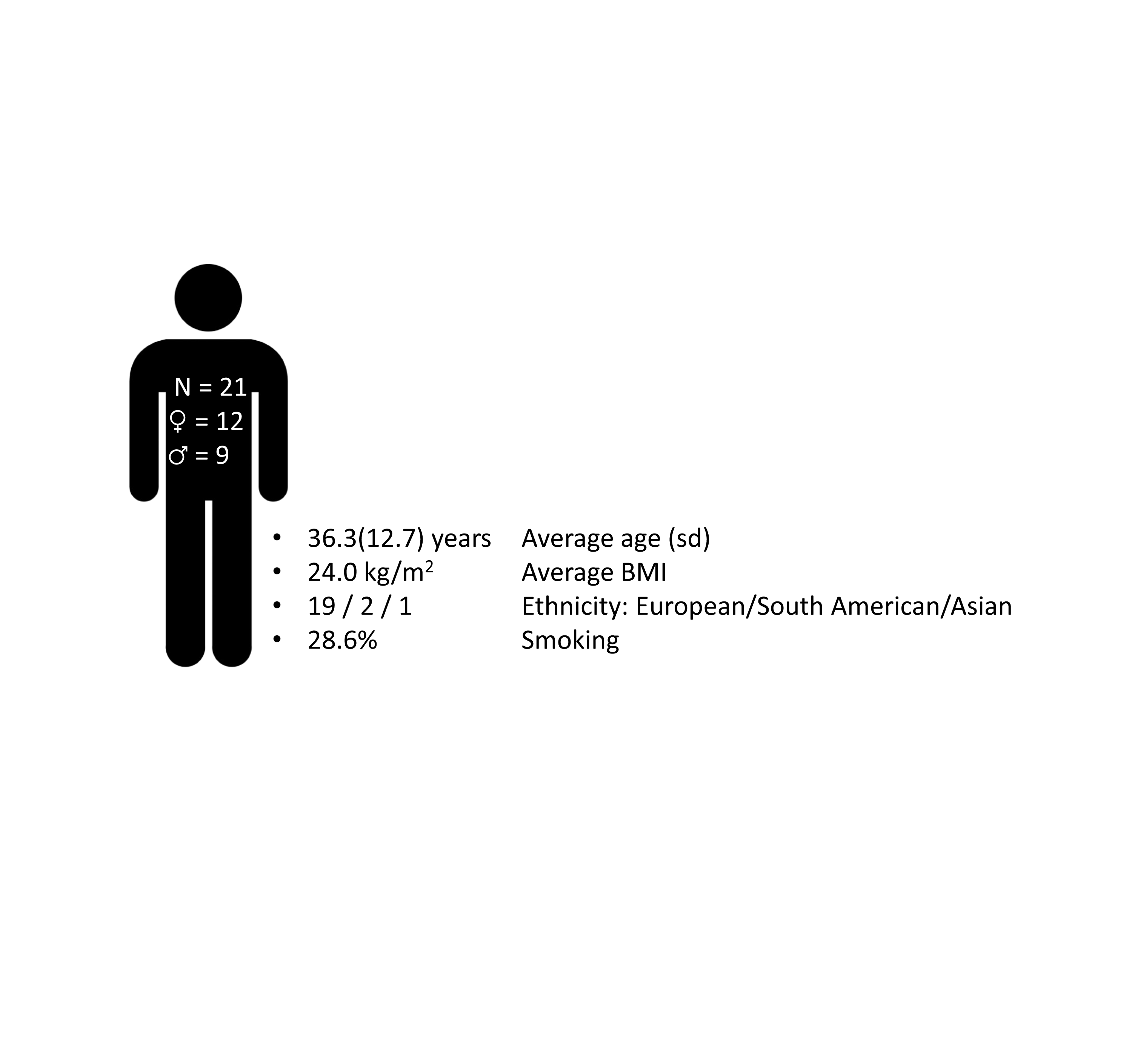

Supplement: Additional file 1: Figure S1. — Baseline characteristics of the GFD study group. (TIF 1068 kb) [file 13073_2016_295_MOESM1_ESM.tif]

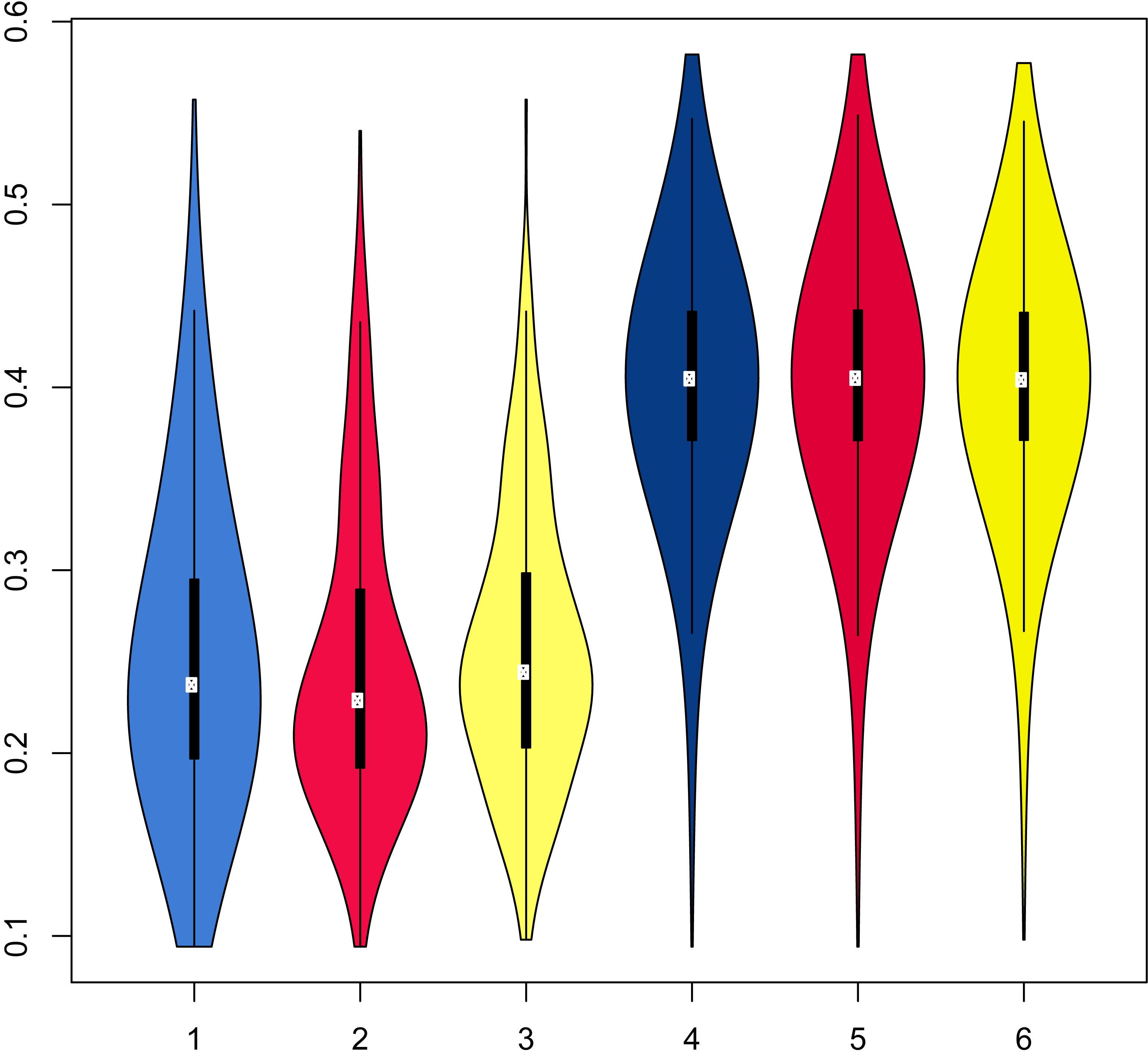

Supplement: Additional file 3: Figure S2. — Unweighted unifrac distances when comparing inter-individual vs intra individual distances. In group 1 the intra-individual differences are shown regardless of diet. Group 2 shows the intra-sample differences are shown within the same diet. Group 3 shows the intra-individual differences are shown between the two diet groups. In group 4 the inter-individual differences are shown regardless of diet. Group 5 shows the inter-sample differences are shown within the same diet. Group 6 shows the inter-individual differences are shown between the two diet groups. The main difference is the intra- vs. inter-individual difference. Also the same diet points in the samples are slightly closer to each other. However, we do not see such a phenomenon for group 5 vs. group 6. (TIF 1862 kb) [file 13073_2016_295_MOESM3_ESM.tif]

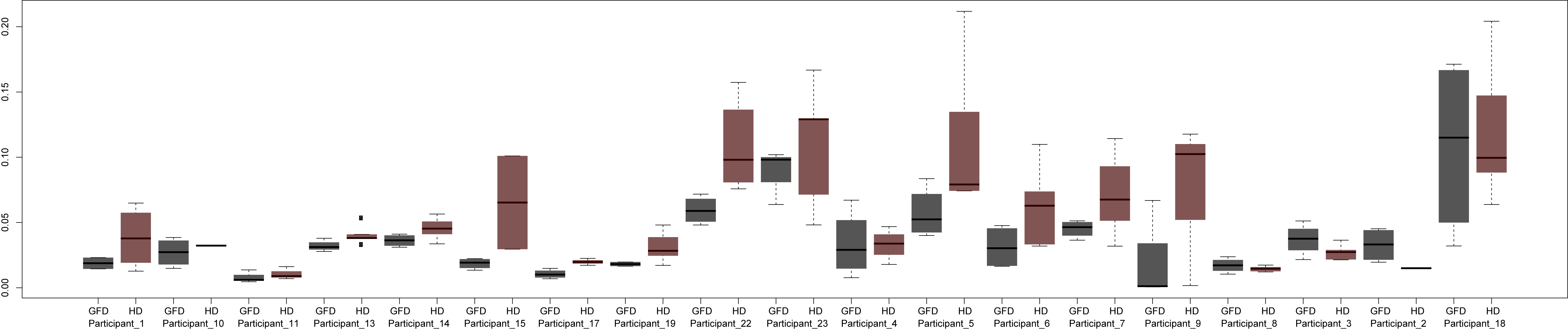

Supplement: Additional file 4: Figure S3. — Abundance of Veillonellaceae family in the GFD participants. In all but four participants we see a clear trend of higher levels of Veillonellaceae on the habitual diet. The rightmost samples do not show this phenomenon. (TIF 4036 kb) [file 13073_2016_295_MOESM4_ESM.tif]

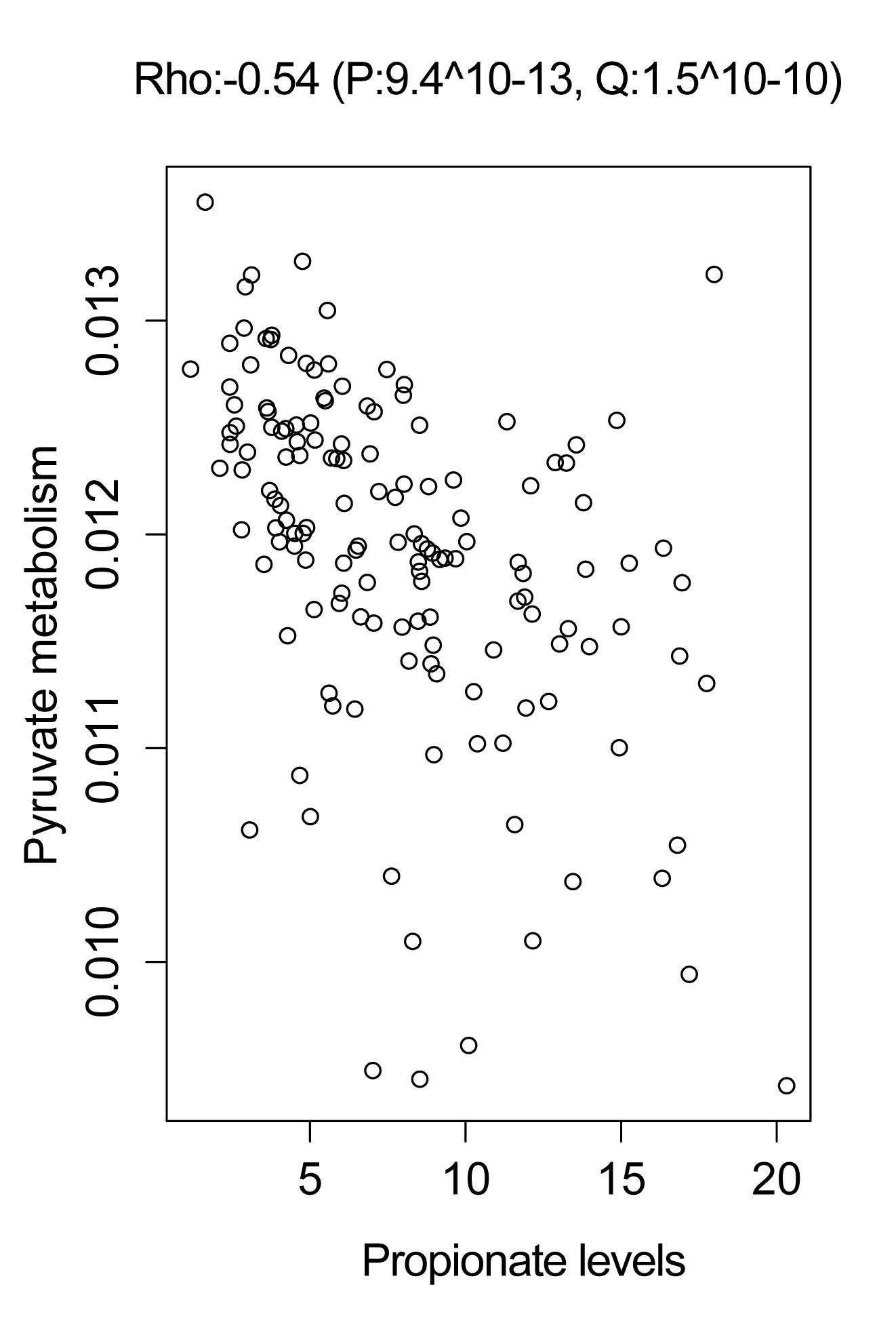

Supplement: Additional file 7: Figure S5. — Measured propionate levels vs. the predicted activity of pyruvate metabolism. (TIF 646 kb) [file 13073_2016_295_MOESM7_ESM.tif]
