# Supplementary material for: The influence of a short-term gluten-free diet on the human gut microbiome
Source: Genome Med. 2016 Apr 21;8:45. doi: 10.1186/s13073-016-0295-y (PMC4841035; doi:10.1186/s13073-016-0295-y)
Supplement: Additional file 8: Table S3. — Correlation of bacteria and levels of fecal biomarkers. (PDF 139 kb) [file 13073_2016_295_MOESM8_ESM.pdf]

Taxa	Fecal_Biomarker	Cor(rho)	P-val	Q-val
k__Bacteria|p__Tenericutes|c__RF3|o__ML615J-28	Chromogranin.A..nmol.g.	-0.58457262	6.17E-15	2.19E-12
k__Bacteria|p__Proteobacteria|c__Betaproteobacteria|o__Burkholderiales|f__Oxalobacteraceae|g__Oxalobacter|s__formigenes	Chromogranin.A..nmol.g.	-0.554156724	2.74E-13	4.88E-11
k__Bacteria|p__Firmicutes|c__Clostridia|o__Clostridiales|f__Lachnospiraceae	Chromogranin.A..nmol.g.	0.514693661	2.20E-11	2.61E-09
k__Bacteria|p__Firmicutes|c__Clostridia|o__Clostridiales|f__Christensenellaceae	Chromogranin.A..nmol.g.	-0.498058166	1.19E-10	9.30E-09
k__Bacteria|p__Tenericutes	Chromogranin.A..nmol.g.	-0.497070916	1.31E-10	9.30E-09
k__Bacteria|p__Firmicutes|c__Clostridia	Valerat...mol.g.	-0.466712823	2.55E-09	1.29E-07
k__Bacteria|p__Firmicutes|c__Clostridia|o__Clostridiales	Valerat...mol.g.	-0.467056627	2.47E-09	1.29E-07
k__Bacteria|p__Firmicutes	Propionat...mol.g.	-0.463051517	3.52E-09	1.39E-07
k__Bacteria|p__Firmicutes|c__Clostridia|o__Clostridiales|f__Christensenellaceae	Butyrat...mol.g.	-0.463749335	3.31E-09	1.39E-07
k__Bacteria|p__Firmicutes|c__Clostridia	Propionat...mol.g.	-0.457405422	5.74E-09	1.92E-07
k__Bacteria|p__Firmicutes|c__Clostridia|o__Clostridiales	Propionat...mol.g.	-0.457003074	5.94E-09	1.92E-07
k__Bacteria|p__Firmicutes	Valerat...mol.g.	-0.455701631	6.64E-09	1.97E-07
k__Bacteria|p__Tenericutes|c__Mollicutes	Chromogranin.A..nmol.g.	-0.449559895	9.94E-09	2.72E-07
k__Bacteria|p__Tenericutes|c__Mollicutes|o__RF39	Chromogranin.A..nmol.g.	-0.448651232	1.07E-08	2.72E-07
k__Bacteria|p__Bacteroidetes|c__Bacteroidia|o__Bacteroidales|f__Barnesiellaceae	Chromogranin.A..nmol.g.	-0.447129747	1.22E-08	2.89E-07
k__Bacteria|p__Bacteroidetes|c__Bacteroidia|o__Bacteroidales|f__Prevotellaceae|g__Prevotella	Caproat...mol.g.	0.436314443	3.31E-08	7.35E-07
k__Bacteria|p__Bacteroidetes|c__Bacteroidia|o__Bacteroidales	Propionat...mol.g.	0.432144392	4.61E-08	9.11E-07
k__Bacteria|p__Bacteroidetes|c__Bacteroidia|o__Bacteroidales|f__Prevotellaceae|g__Prevotella	Valerat...mol.g.	0.432271363	4.57E-08	9.11E-07
k__Bacteria|p__Firmicutes|c__Clostridia|o__Clostridiales|f__Christensenellaceae	Propionat...mol.g.	-0.425141258	7.99E-08	1.49E-06
k__Bacteria|p__Bacteroidetes|c__Bacteroidia|o__Bacteroidales|f__S24-7	Chromogranin.A..nmol.g.	-0.419289581	1.13E-07	2.01E-06
k__Bacteria|p__Bacteroidetes|c__Bacteroidia|o__Bacteroidales|f__Odoribacteraceae|g__Butyricimonas	Caproat...mol.g.	0.411558139	2.24E-07	3.78E-06
k__Bacteria|p__Firmicutes|c__Clostridia|o__Clostridiales|f__Lachnospiraceae	Valerat...mol.g.	-0.40796191	2.91E-07	4.71E-06
k__Bacteria|p__Firmicutes|c__Clostridia|o__Clostridiales|f__Ruminococcaceae|g__Faecalibacterium|s__prausnitzii	Caproat...mol.g.	-0.405265259	3.55E-07	5.48E-06
k__Bacteria|p__Firmicutes|c__Clostridia|o__Clostridiales|f__Christensenellaceae	Acetat...mol.g.	-0.404176961	3.84E-07	5.68E-06
k__Bacteria|p__Proteobacteria|c__Alphaproteobacteria|o__RF32	Chromogranin.A..nmol.g.	-0.395994676	6.30E-07	8.95E-06
k__Bacteria|p__Bacteroidetes|c__Bacteroidia|o__Bacteroidales|f__Odoribacteraceae|g__Butyricimonas	Chromogranin.A..nmol.g.	-0.394272118	7.11E-07	9.72E-06
k__Bacteria|p__Bacteroidetes|c__Bacteroidia|o__Bacteroidales	Valerat...mol.g.	0.39192966	9.15E-07	1.20E-05
k__Bacteria|p__Bacteroidetes|c__Bacteroidia|o__Bacteroidales|f__Prevotellaceae|g__Prevotella|s__copri	Caproat...mol.g.	0.39043408	1.01E-06	1.29E-05
k__Bacteria|p__Bacteroidetes|c__Bacteroidia|o__Bacteroidales|f__S24-7	Calprotectin...g.g.	-0.387015125	1.18E-06	1.45E-05
k__Bacteria|p__Bacteroidetes|c__Bacteroidia|o__Bacteroidales|f__[Bacteroidaceae/Rikenellaceae]	Caproat...mol.g.	0.384274798	1.55E-06	1.83E-05
k__Bacteria|p__Firmicutes|c__Clostridia|o__Clostridiales|f__Lachnospiraceae|g__Roseburia	Chromogranin.A..nmol.g.	0.382009099	1.66E-06	1.90E-05
k__Bacteria|p__Firmicutes|c__Clostridia|o__Clostridiales|f__Clostridiaceae|g__Clostridium	Chromogranin.A..nmol.g.	0.375309125	2.60E-06	2.84E-05
k__Bacteria|p__Firmicutes|c__Clostridia|o__Clostridiales|f__Veillonellaceae	Propionat...mol.g.	0.376284137	2.64E-06	2.84E-05
k__Bacteria|p__Firmicutes|c__Clostridia|o__Clostridiales|f__Veillonellaceae	Acetat...mol.g.	0.366750378	4.90E-06	5.13E-05
k__Bacteria|p__Firmicutes|c__Clostridia|o__Clostridiales|f__Ruminococcaceae	Propionat...mol.g.	-0.366053543	5.13E-06	5.21E-05
k__Bacteria|p__Firmicutes|c__Clostridia|o__Clostridiales|f__Veillonellaceae|g__Acidaminococcus	Calprotectin...g.g.	0.362521918	5.96E-06	5.88E-05
k__Bacteria|p__Firmicutes|c__Clostridia|o__Clostridiales|f__Lachnospiraceae|g__Coprococcus	Chromogranin.A..nmol.g.	-0.360132946	6.93E-06	6.66E-05
k__Bacteria|p__Bacteroidetes|c__Bacteroidia|o__Bacteroidales|f__Prevotellaceae|g__Prevotella|s__copri	Valerat...mol.g.	0.357166552	8.96E-06	8.38E-05
k__Bacteria|p__Firmicutes|c__Clostridia|o__Clostridiales|f__Lachnospiraceae|g__Roseburia	Valerat...mol.g.	-0.354948519	1.03E-05	9.36E-05
k__Bacteria|p__Firmicutes|c__Clostridia|o__Clostridiales|f__Lachnospiraceae	Caproat...mol.g.	-0.351817725	1.24E-05	0.000110551
k__Bacteria|p__Firmicutes|c__Bacilli|o__Lactobacillales	Butyrat...mol.g.	0.350448326	1.35E-05	0.000117197
k__Archaea|p__Euryarchaeota|c__Methanobacteria|o__Methanobacteriales|f__Methanobacteriaceae|g__Methanobrevibacter	Chromogranin.A..nmol.g.	-0.346716935	1.58E-05	0.000130772
k__Bacteria	Chromogranin.A..nmol.g.	0.346915308	1.56E-05	0.000130772
k__Bacteria|p__Bacteroidetes|c__Bacteroidia|o__Bacteroidales|f__Prevotellaceae|g__Prevotella	Propionat...mol.g.	0.346233382	1.74E-05	0.000140538

k__Bacteria|p__Bacteroidetes|c__Bacteroidia|o__Bacteroidales|f__Barnesiellaceae	Caproat...mol.g.	0.345874052	1.78E-05	0.000140538
k__Bacteria|p__Firmicutes|c__Erysipelotrichi|o__Erysipelotrichales|f__Erysipelotrichaceae	Acetat...mol.g.	-0.340031545	2.51E-05	0.000194067
k__Bacteria|p__Firmicutes|c__Clostridia|o__Clostridiales|f__Ruminococcaceae|g__Oscillospira	Butyrat...mol.g.	-0.339012186	2.67E-05	0.00020157
k__Bacteria|p__Firmicutes|c__Clostridia|o__Clostridiales|f__Veillonellaceae	Butyrat...mol.g.	0.338046926	2.82E-05	0.000208758
k__Bacteria|p__Firmicutes|c__Clostridia|o__Clostridiales|f__[Ruminococcaceae/Clostridiaceae]	Beta.Defensin.2..ng.g.	-0.33610515	2.96E-05	0.000214812
k__Bacteria|p__Bacteroidetes|c__Bacteroidia|o__Bacteroidales|f__Rikenellaceae	Caproat...mol.g.	0.336199485	3.14E-05	0.000223004
k__Bacteria|p__Bacteroidetes|c__Bacteroidia|o__Bacteroidales|f__Bacteroidaceae|g__Bacteroides|s__coprophilus	Beta.Defensin.2..ng.g.	0.332058885	3.74E-05	0.00025432
k__Bacteria|p__Bacteroidetes|c__Bacteroidia|o__Bacteroidales|f__Odoribacteraceae|g__Butyricimonas	Beta.Defensin.2..ng.g.	0.3323298	3.68E-05	0.00025432
k__Bacteria|p__Lentisphaerae|c__Lentisphaeria|o__Victivallales|f__Victivallaceae	Calprotectin...g.g.	-0.331810232	3.79E-05	0.00025432
k__Bacteria|p__Proteobacteria|c__Betaproteobacteria|o__Burkholderiales|f__Oxalobacteraceae|g__Oxalobacter|s__formigenes	Calprotectin...g.g.	-0.331218068	3.92E-05	0.000258194
k__Bacteria|p__Firmicutes|c__Bacilli	Butyrat...mol.g.	0.327407956	5.17E-05	0.000328011
k__Bacteria|p__Firmicutes|c__Clostridia|o__SHA-98|365762	Butyrat...mol.g.	-0.327633752	5.10E-05	0.000328011
k__Bacteria|p__Firmicutes|c__Clostridia|o__Clostridiales|f__[Ruminococcaceae/Lachnospiraceae]	Chromogranin.A..nmol.g.	-0.324818376	5.63E-05	0.000344898
k__Bacteria|p__Firmicutes|c__Clostridia|o__Clostridiales|f__[Ruminococcaceae/[Mogibacteriaceae]]	Caproat...mol.g.	0.326028981	5.58E-05	0.000344898
k__Bacteria|p__Bacteroidetes|c__Bacteroidia|o__Bacteroidales|f__[Bacteroidaceae/Rikenellaceae]	Valerat...mol.g.	0.323783905	6.32E-05	0.000380787
k__Bacteria|p__Firmicutes|c__Bacilli|o__Lactobacillales|f__Lactobacillaceae|g__Lactobacillus|s__ruminis	Chromogranin.A..nmol.g.	0.318025476	8.19E-05	0.00048483
k__Bacteria|p__Firmicutes|c__Clostridia|o__Clostridiales|f__Lachnospiraceae|g__Blautia|s__obeum	Propionat...mol.g.	-0.3181936	8.58E-05	0.000499988
k__Bacteria|p__Bacteroidetes|c__Bacteroidia|o__Bacteroidales|f__Odoribacteraceae	Valerat...mol.g.	0.317225642	9.04E-05	0.000518347
k__Bacteria|p__Bacteroidetes|c__Bacteroidia|o__Bacteroidales|f__Bacteroidaceae|g__Bacteroides	Caproat...mol.g.	-0.314203907	0.000106354	0.000599944
k__Bacteria|p__Bacteroidetes|c__Bacteroidia|o__Bacteroidales|f__Prevotellaceae|g__Prevotella|s__copri	Propionat...mol.g.	0.313438017	0.000110787	0.000615185
k__Bacteria|p__Firmicutes|c__Clostridia|o__Clostridiales|f__[Ruminococcaceae/Lachnospiraceae]	Caproat...mol.g.	0.311970938	0.000119763	0.0006548
k__Bacteria|p__Firmicutes|c__Clostridia|o__Clostridiales|f__[Veillonellaceae/Clostridiaceae]	Calprotectin...g.g.	0.310662328	0.000121612	0.000654835
k__Bacteria|p__Bacteroidetes|c__Bacteroidia|o__Bacteroidales|f__Odoribacteraceae|g__Odoribacter	Valerat...mol.g.	0.3112456	0.000124448	0.000660103
k__Bacteria|p__Bacteroidetes|c__Bacteroidia|o__Bacteroidales|f__Bacteroidaceae|g__Bacteroides|s__plebeius	Beta.Defensin.2..ng.g.	0.309536908	0.000129081	0.000664834
k__Bacteria|p__Firmicutes|c__Clostridia|o__Clostridiales|f__Lachnospiraceae|g__Roseburia|s__faecis	Butyrat...mol.g.	0.310568816	0.000128973	0.000664834
k__Bacteria|p__Firmicutes|c__Clostridia|o__Clostridiales|f__Ruminococcaceae|g__Oscillospira	Acetat...mol.g.	-0.310274643	0.000130987	0.00066501
k__Bacteria|p__Firmicutes|c__Clostridia|o__Clostridiales|f__Veillonellaceae|g__Dialister	Acetat...mol.g.	0.309350114	0.00013751	0.000688293
k__Bacteria|p__Firmicutes|c__Clostridia|o__Clostridiales|f__Clostridiaceae|g__Clostridium	Calprotectin...g.g.	0.305876397	0.000156439	0.000772168
k__Bacteria|p__Firmicutes|c__Clostridia|o__Clostridiales|f__Lachnospiraceae|g__Dorea	Acetat...mol.g.	-0.306400054	0.000160404	0.000780892
k__Bacteria|p__Firmicutes|c__Clostridia|o__Clostridiales|f__Lachnospiraceae	Beta.Defensin.2..ng.g.	-0.304735039	0.000166017	0.000797297
k__Bacteria|p__Firmicutes|c__Clostridia|o__Clostridiales|f__Peptococcaceae|g__rc4-4	Chromogranin.A..nmol.g.	-0.30424724	0.000170275	0.000806842
k__Bacteria|p__Firmicutes|c__Clostridia|o__Clostridiales|f__Lachnospiraceae|g__Roseburia|s__faecis	Chromogranin.A..nmol.g.	0.303829674	0.000174	0.000813645
k__Bacteria|p__Tenericutes	Caproat...mol.g.	0.30334239	0.000187844	0.00086697
k__Bacteria|p__Firmicutes|c__Clostridia|o__Clostridiales|f__Lachnospiraceae|g__Roseburia	Caproat...mol.g.	-0.302404719	0.000197096	0.00089801
k__Bacteria|p__Firmicutes|c__Bacilli|o__Lactobacillales|f__Streptococcaceae	Butyrat...mol.g.	0.301378143	0.00020771	0.000923455
k__Bacteria|p__Firmicutes|c__Bacilli|o__Lactobacillales|f__Streptococcaceae|g__Streptococcus	Butyrat...mol.g.	0.301362377	0.000207877	0.000923455
k__Bacteria|p__Firmicutes|c__Bacilli|o__Lactobacillales	Acetat...mol.g.	0.301000619	0.000211746	0.000929026
k__Bacteria|p__Bacteroidetes|c__Bacteroidia|o__Bacteroidales|f__Bacteroidaceae|g__Bacteroides|s__fragilis	Caproat...mol.g.	-0.299823292	0.000224802	0.000974284
k__Bacteria|p__Bacteroidetes|c__Bacteroidia|o__Bacteroidales|f__Bacteroidaceae|g__Bacteroides|s__fragilis	Beta.Defensin.2..ng.g.	-0.298451729	0.00022927	0.000981675
k__Bacteria|p__Firmicutes|c__Clostridia|o__Clostridiales|f__Veillonellaceae|g__Acidaminococcus	Chromogranin.A..nmol.g.	0.297182456	0.000244503	0.001034319
k__Bacteria|p__Firmicutes|c__Clostridia|o__Clostridiales|f__Ruminococcaceae	Valerat...mol.g.	-0.297929333	0.000247386	0.001034319
k__Bacteria|p__Firmicutes	Acetat...mol.g.	-0.297359913	0.000254577	0.001052007
k__Bacteria|p__Firmicutes|c__Clostridia|o__Clostridiales|f__Lachnospiraceae|g__Roseburia|s__faecis	Acetat...mol.g.	0.296608991	0.000264356	0.001079863
k__Bacteria|p__Firmicutes	Caproat...mol.g.	-0.294582347	0.000292517	0.001181317
k__Bacteria|p__Firmicutes|c__Clostridia|o__Clostridiales|f__Ruminococcaceae|g__Ruminococcus	Butyrat...mol.g.	-0.293569403	0.000307611	0.001228318

k__Bacteria|p__Bacteroidetes|c__Bacteroidia|o__Bacteroidales|f__Bacteroidaceae|g__Bacteroides|s__caccae	Chromogranin.A..nmol.g.	-0.291766297	0.000320668	0.001243229
k__Bacteria|p__Firmicutes|c__Bacilli	Acetat...mol.g.	0.292436985	0.000325339	0.001243229
k__Bacteria|p__Firmicutes|c__Clostridia|o__Clostridiales|f__Ruminococcaceae|g__Oscillospira	Propionat...mol.g.	-0.292597647	0.000322767	0.001243229
k__Bacteria|p__Tenericutes|c__Mollicutes	Caproat...mol.g.	0.292611343	0.000322549	0.001243229
k__Bacteria|p__Firmicutes|c__Clostridia|o__Clostridiales|f__Ruminococcaceae|g__Ruminococcus	Propionat...mol.g.	-0.291955402	0.000333161	0.001259249
k__Bacteria|p__Tenericutes|c__Mollicutes|o__RF39	Caproat...mol.g.	0.291745892	0.000336617	0.001259249
k__Bacteria|p__Firmicutes|c__Clostridia|o__Clostridiales|f__Ruminococcaceae|g__Faecalibacterium|s__prausnitzii	Beta.Defensin.2..ng.g.	-0.287447051	0.000396571	0.001447306
k__Bacteria|p__Firmicutes|c__Clostridia|o__Clostridiales|f__Ruminococcaceae	Acetat...mol.g.	-0.288266523	0.000399106	0.001447306
k__Bacteria|p__Bacteroidetes|c__Bacteroidia|o__Bacteroidales|f__Bacteroidaceae|g__Bacteroides|s__caccae	Caproat...mol.g.	0.28849788	0.000394639	0.001447306
k__Bacteria|p__Firmicutes|c__Clostridia|o__Clostridiales|f__Lachnospiraceae|g__Ruminococcus|s__gnavus	Chromogranin.A..nmol.g.	0.285675978	0.000432248	0.001551659
k__Bacteria|p__Firmicutes|c__Clostridia|o__Clostridiales|f__Lachnospiraceae|g__Coprococcus|s__catus	Chromogranin.A..nmol.g.	-0.284153533	0.000465262	0.0016371
k__Bacteria|p__Firmicutes|c__Clostridia|o__Clostridiales|f__Lachnospiraceae|g__Lachnospira	Chromogranin.A..nmol.g.	0.284252309	0.000463052	0.0016371
k__Bacteria|p__Proteobacteria|c__Alphaproteobacteria|o__RF32	Acetat...mol.g.	-0.284522506	0.000478207	0.001666151
k__Bacteria|p__Firmicutes|c__Clostridia|o__Clostridiales|f__Lachnospiraceae|g__Coprococcus|s__eutactus	Chromogranin.A..nmol.g.	-0.281926719	0.00051776	0.00178193
k__Bacteria|p__Firmicutes|c__Clostridia	Beta.Defensin.2..ng.g.	-0.281777553	0.000521465	0.00178193
k__Bacteria|p__Firmicutes|c__Clostridia|o__Clostridiales	Beta.Defensin.2..ng.g.	-0.281370342	0.000531704	0.001797528
k__Bacteria|p__Proteobacteria|c__Alphaproteobacteria|o__RF32	Butyrat...mol.g.	-0.282127542	0.000536146	0.001797528
k__Bacteria|p__Lentisphaerae|c__Lentisphaeria|o__Victivallales|f__Victivallaceae	Chromogranin.A..nmol.g.	-0.280181618	0.000562663	0.001840334
k__Bacteria|p__Firmicutes|c__Clostridia|o__Clostridiales|f__[Clostridiaceae/Lachnospiraceae]	Calprotectin...g.g.	-0.28027055	0.000560291	0.001840334
k__Bacteria|p__Firmicutes|c__Clostridia|o__Clostridiales|f__Veillonellaceae	Valerat...mol.g.	0.281043239	0.000564449	0.001840334
k__Bacteria|p__Firmicutes|c__Erysipelotrichi|o__Erysipelotrichales|f__Erysipelotrichaceae|g__cc_115	Butyrat...mol.g.	-0.280609941	0.00057614	0.001861373
k__Bacteria|p__Bacteroidetes|c__Bacteroidia|o__Bacteroidales|f__Porphyromonadaceae|g__Parabacteroides|s__distasonis	Propionat...mol.g.	-0.279584233	0.000604708	0.001918316
k__Bacteria|p__Bacteroidetes|c__Bacteroidia|o__Bacteroidales|f__S24-7	Valerat...mol.g.	0.279213314	0.000615356	0.001918316
k__Bacteria|p__Firmicutes|c__Clostridia	Caproat...mol.g.	-0.279219276	0.000615184	0.001918316
k__Bacteria|p__Firmicutes|c__Clostridia|o__Clostridiales	Caproat...mol.g.	-0.279245738	0.000614418	0.001918316
k__Bacteria|p__Firmicutes	Butyrat...mol.g.	-0.278952608	0.000622943	0.001925082
k__Bacteria|p__Bacteroidetes|c__Bacteroidia|o__Bacteroidales	Acetat...mol.g.	0.277520304	0.000666186	0.002040967
k__Bacteria|p__Bacteroidetes|c__Bacteroidia|o__Bacteroidales|f__Bacteroidaceae|g__Bacteroides|s__ovatus	Caproat...mol.g.	-0.277222607	0.000675513	0.002051855
k__Bacteria|p__Bacteroidetes|c__Bacteroidia|o__Bacteroidales|f__[Bacteroidaceae/Rikenellaceae]	Beta.Defensin.2..ng.g.	0.274122988	0.000747893	0.002252453
k__Bacteria|p__Firmicutes|c__Clostridia|o__SHA-98|365762	Propionat...mol.g.	-0.273119191	0.000816932	0.002439704
k__Bacteria|p__Firmicutes|c__Clostridia|o__Clostridiales|f__Lachnospiraceae|g__Blautia	Beta.Defensin.2..ng.g.	-0.271652264	0.000838377	0.002482884
k__Bacteria|p__Firmicutes|c__Clostridia|o__Clostridiales|f__Ruminococcaceae|g__Faecalibacterium|s__prausnitzii	Valerat...mol.g.	-0.271686843	0.000872375	0.002562218
k__Bacteria|p__Proteobacteria|c__Betaproteobacteria|o__Burkholderiales	Calprotectin...g.g.	-0.270255981	0.000893853	0.002603781
k__Archaea|p__Euryarchaeota|c__Methanobacteria|o__Methanobacteriales|f__Methanobacteriaceae|g__Methanobrevibacter	Butyrat...mol.g.	-0.270737213	0.000911014	0.002610969
k__Bacteria	Butyrat...mol.g.	0.270764776	0.00090987	0.002610969
k__Bacteria|p__Firmicutes|c__Clostridia|o__Clostridiales|f__Lachnospiraceae|g__Blautia|s__obeum	Acetat...mol.g.	-0.269767856	0.000952069	0.002686372
k__Bacteria|p__Firmicutes|c__Clostridia|o__Clostridiales|f__Christensenellaceae|g__Christensenella	Caproat...mol.g.	0.269759238	0.000952441	0.002686372
k__Bacteria|p__Firmicutes|c__Bacilli	Chromogranin.A..nmol.g.	0.267784813	0.001000357	0.002777431
k__Bacteria|p__Bacteroidetes|c__Bacteroidia|o__Bacteroidales|f__Rikenellaceae	Valerat...mol.g.	0.268768283	0.000996174	0.002777431
k__Bacteria|p__Firmicutes|c__Clostridia|o__Clostridiales|f__Lachnospiraceae|g__Coprococcus|s__catus	Acetat...mol.g.	-0.267952763	0.001033535	0.002847305
k__Bacteria|p__Firmicutes|c__Clostridia|o__Clostridiales|f__Ruminococcaceae|g__Ruminococcus|s__flavefaciens	Chromogranin.A..nmol.g.	-0.266499375	0.001060241	0.00289663
k__Bacteria|p__Firmicutes|c__Clostridia|o__Clostridiales|f__Peptococcaceae|g__rc4-4	Acetat...mol.g.	-0.267229472	0.001067741	0.00289663
k__Bacteria|p__Actinobacteria|c__Coriobacteriia|o__Coriobacteriales|f__Coriobacteriaceae|g__Eggerthella|s__lenta	Caproat...mol.g.	-0.266885405	0.001084373	0.002919466
k__Bacteria|p__Tenericutes|c__RF3|o__ML615J-28	Calprotectin...g.g.	-0.264685277	0.001150348	0.003073804
k__Bacteria|p__Bacteroidetes|c__Bacteroidia|o__Bacteroidales|f__Odoribacteraceae|g__Butyricimonas	Valerat...mol.g.	0.26530627	0.001163795	0.003086526

k__Bacteria|p__Bacteroidetes|c__Bacteroidia|o__Bacteroidales|f__Prevotellaceae|g__Prevotella	Butyrat...mol.g.	0.264543102	0.001204053	0.003169644
k__Bacteria|p__Bacteroidetes|c__Bacteroidia|o__Bacteroidales|f__Bacteroidaceae|g__Bacteroides|s__ovatus	Chromogranin.A..nmol.g.	0.262525606	0.001266727	0.00328595
k__Bacteria|p__Firmicutes|c__Clostridia|o__Clostridiales|f__Lachnospiraceae|g__[Lachnospira/Roseburia]	Acetat...mol.g.	0.263405509	0.001266431	0.00328595
k__Bacteria|p__Firmicutes|c__Clostridia|o__Clostridiales|f__Lachnospiraceae|g__Coprococcus|s__catus	Propionat...mol.g.	-0.263203206	0.001277828	0.003290727
k__Bacteria|p__Bacteroidetes|c__Bacteroidia|o__Bacteroidales|f__[Bacteroidaceae/Rikenellaceae]	Chromogranin.A..nmol.g.	-0.261336566	0.0013353	0.003404064
k__Bacteria|p__Proteobacteria|c__Betaproteobacteria|o__Burkholderiales|f__Alcaligenaceae|g__Sutterella	Calprotectin...g.g.	-0.261240332	0.001340995	0.003404064
k__Bacteria|p__Firmicutes|c__Clostridia|o__Clostridiales|f__Peptococcaceae|g__rc4-4	Butyrat...mol.g.	-0.261632213	0.001369574	0.003451951
k__Bacteria|p__Bacteroidetes|c__Bacteroidia|o__Bacteroidales|f__Odoribacteraceae	Caproat...mol.g.	0.261237832	0.00139353	0.003487599
k__Bacteria|p__Firmicutes|c__Clostridia|o__Clostridiales|f__Ruminococcaceae|g__Ruminococcus|s__flavefaciens	Beta.Defensin.2..ng.g.	0.25893675	0.001484126	0.003670847
k__Bacteria|p__Bacteroidetes|c__Bacteroidia|o__Bacteroidales|f__Prevotellaceae|g__Prevotella|s__copri	Acetat...mol.g.	0.259749729	0.001487409	0.003670847
k__Bacteria|p__Actinobacteria|c__Coriobacteriia|o__Coriobacteriales|f__Coriobacteriaceae|g__Eggerthella|s__lenta	Chromogranin.A..nmol.g.	0.257738908	0.001563935	0.003833092
k__Bacteria|p__Proteobacteria	Calprotectin...g.g.	-0.257182693	0.001602308	0.003893918
k__Bacteria|p__Bacteroidetes|c__Bacteroidia|o__Bacteroidales	Butyrat...mol.g.	0.257920894	0.001610667	0.003893918
k__Bacteria|p__Bacteroidetes|c__Bacteroidia|o__Bacteroidales|f__Porphyromonadaceae|g__Parabacteroides|s__gordonii	Calprotectin...g.g.	-0.256685253	0.00163735	0.00393168
k__Bacteria|p__Bacteroidetes|c__Bacteroidia|o__Bacteroidales|f__Bacteroidaceae|g__Bacteroides|s__ovatus	Beta.Defensin.2..ng.g.	-0.25598308	0.001688003	0.003972781
k__Bacteria|p__Firmicutes|c__Bacilli|o__Lactobacillales|f__Streptococcaceae	Acetat...mol.g.	0.2568864	0.00168444	0.003972781
k__Bacteria|p__Firmicutes|c__Clostridia	Butyrat...mol.g.	-0.25714453	0.001665749	0.003972781
k__Bacteria|p__Firmicutes|c__Clostridia|o__Clostridiales	Butyrat...mol.g.	-0.256632621	0.001703001	0.003981712
k__Bacteria|p__Proteobacteria|c__Gammaproteobacteria|o__Aeromonadales|f__Succinivibrionaceae|g__Succinivibrio	Calprotectin...g.g.	0.255569569	0.001718496	0.003991678
k__Bacteria|p__Firmicutes|c__Erysipelotrichi|o__Erysipelotrichales|f__Erysipelotrichaceae	Butyrat...mol.g.	-0.255574099	0.001782447	0.004113336
k__Bacteria|p__Proteobacteria|c__Alphaproteobacteria|o__RF32	Propionat...mol.g.	-0.254186558	0.00189169	0.004309469
k__Bacteria|p__Firmicutes|c__Clostridia|o__Clostridiales|f__Lachnospiraceae|g__Roseburia|s__faecis	Caproat...mol.g.	-0.254294182	0.001883004	0.004309469
k__Bacteria|p__Firmicutes|c__Clostridia|o__Clostridiales|f__[Ruminococcaceae/[Mogibacteriaceae]]	Chromogranin.A..nmol.g.	-0.25292671	0.00192552	0.004331012
k__Bacteria|p__Bacteroidetes|c__Bacteroidia|o__Bacteroidales|f__Paraprevotellaceae|g__Prevotella	Butyrat...mol.g.	0.253853651	0.001918789	0.004331012
k__Bacteria|p__Bacteroidetes|c__Bacteroidia|o__Bacteroidales|f__Rikenellaceae	Chromogranin.A..nmol.g.	-0.252617687	0.001951151	0.004361062
k__Bacteria|p__Bacteroidetes|c__Bacteroidia|o__Bacteroidales|f__Bacteroidaceae|g__Bacteroides|s__caccae	Valerat...mol.g.	0.253057483	0.001985036	0.004409068
k__Archaea|p__Euryarchaeota|c__Methanobacteria|o__Methanobacteriales|f__Methanobacteriaceae|g__Methanobrevibacter	Calprotectin...g.g.	-0.250427215	0.002141908	0.004698771
k__Bacteria	Calprotectin...g.g.	0.25051229	0.002134194	0.004698771
k__Bacteria|p__Firmicutes|c__Clostridia	Acetat...mol.g.	-0.250043682	0.002255046	0.004916616
k__Bacteria|p__Firmicutes|c__Clostridia|o__Clostridiales	Acetat...mol.g.	-0.249254102	0.002331086	0.005051414
k__Bacteria|p__Firmicutes|c__Clostridia|o__Clostridiales|f__Lachnospiraceae|g__Roseburia	Beta.Defensin.2..ng.g.	-0.24732788	0.002440768	0.005207687
k__Bacteria|p__Firmicutes|c__Bacilli|o__Lactobacillales|f__Streptococcaceae|g__Streptococcus	Acetat...mol.g.	0.248213605	0.002434838	0.005207687
k__Bacteria|p__Firmicutes|c__Clostridia|o__Clostridiales|f__Veillonellaceae|g__Dialister	Butyrat...mol.g.	0.248092685	0.002447163	0.005207687
k__Bacteria|p__Firmicutes|c__Clostridia|o__Clostridiales|f__Veillonellaceae|g__Dialister	Propionat...mol.g.	0.247104879	0.00254998	0.005394186
k__Bacteria|p__Proteobacteria|c__Betaproteobacteria|o__Burkholderiales|f__Oxalobacteraceae|g__Oxalobacter|s__formigenes	Acetat...mol.g.	-0.24685968	0.0025761	0.005417195
k__Bacteria|p__Firmicutes	Beta.Defensin.2..ng.g.	-0.245052609	0.002683677	0.005610217
k__Bacteria|p__Bacteroidetes|c__Bacteroidia|o__Bacteroidales|f__Prevotellaceae|g__Prevotella	Acetat...mol.g.	0.244152465	0.002881013	0.005918436
k__Bacteria|p__Tenericutes	Acetat...mol.g.	-0.244294187	0.002864275	0.005918436
k__Bacteria|p__Synergistetes|c__Synergistia|o__Synergistales|f__Synergistaceae	Valerat...mol.g.	-0.244151935	0.002881075	0.005918436
k__Bacteria|p__Bacteroidetes|c__Bacteroidia|o__Bacteroidales|f__Prevotellaceae|g__Prevotella|s__stercorea	Beta.Defensin.2..ng.g.	0.243077133	0.002912087	0.00594776
k__Bacteria|p__Proteobacteria|c__Betaproteobacteria|o__Burkholderiales|f__Oxalobacteraceae|g__Oxalobacter|s__formigenes	Butyrat...mol.g.	-0.243540634	0.002954287	0.005999472
k__Bacteria|p__Firmicutes|c__Bacilli|o__Lactobacillales	Chromogranin.A..nmol.g.	0.242182786	0.003021153	0.006065938
k__Bacteria|p__Bacteroidetes|c__Bacteroidia|o__Bacteroidales	Caproat...mol.g.	0.243038186	0.003015715	0.006065938
k__Bacteria|p__Bacteroidetes|c__Bacteroidia|o__Bacteroidales|f__Odoribacteraceae|g__Odoribacter	Caproat...mol.g.	0.242583929	0.003072242	0.006133859
k__Bacteria|p__Firmicutes|c__Clostridia|o__Clostridiales|f__Ruminococcaceae	Butyrat...mol.g.	-0.24173437	0.003180531	0.006314587

k__Bacteria|p__Actinobacteria|c__Actinobacteria|o__Bifidobacteriales|f__Bifidobacteriaceae|g__Bifidobacterium|s__adolescentis	Butyrat...mol.g.	0.240889812	0.003291583	0.006498763
k__Bacteria|p__Bacteroidetes|c__Bacteroidia|o__Bacteroidales|f__Bacteroidaceae|g__Bacteroides|s__coprophilus	Acetat...mol.g.	0.240748372	0.003310519	0.006500038
k__Bacteria|p__Bacteroidetes|c__Bacteroidia|o__Bacteroidales|f__Prevotellaceae|g__Prevotella|s__copri	Butyrat...mol.g.	0.240441657	0.00335192	0.006516196
k__Bacteria|p__Firmicutes|c__Clostridia|o__Clostridiales|f__Dehalobacteriaceae|g__Dehalobacterium	Butyrat...mol.g.	-0.240415887	0.003355419	0.006516196
k__Bacteria|p__Firmicutes|c__Clostridia|o__Clostridiales|f__Veillonellaceae|g__Acidaminococcus	Propionat...mol.g.	0.23963504	0.003463035	0.006688634
k__Bacteria|p__Firmicutes|c__Clostridia|o__Clostridiales|f__Lachnospiraceae|g__Ruminococcus|s__gnavus	Caproat...mol.g.	-0.238377577	0.003642883	0.006997967
k__Bacteria|p__Firmicutes|c__Clostridia|o__Clostridiales|f__Dehalobacteriaceae|g__Dehalobacterium	Acetat...mol.g.	-0.238038624	0.003692778	0.007017946
k__Bacteria|p__Firmicutes|c__Erysipelotrichi|o__Erysipelotrichales|f__Erysipelotrichaceae	Propionat...mol.g.	-0.238167558	0.003673726	0.007017946
k__Bacteria|p__Firmicutes|c__Clostridia|o__Clostridiales|f__Lachnospiraceae|g__Blautia	Propionat...mol.g.	-0.237765699	0.0037334	0.007057406
k__Bacteria|p__Bacteroidetes|c__Bacteroidia|o__Bacteroidales|f__Paraprevotellaceae|g__Prevotella	Valerat...mol.g.	0.236765845	0.003885673	0.00730639
k__Bacteria|p__Bacteroidetes|c__Bacteroidia|o__Bacteroidales|f__Porphyromonadaceae|g__Parabacteroides|s__distasonis	Acetat...mol.g.	-0.236432619	0.003937648	0.007365153
k__Bacteria|p__Bacteroidetes|c__Bacteroidia|o__Bacteroidales|f__Porphyromonadaceae|g__Parabacteroides	Beta.Defensin.2..ng.g.	-0.234039383	0.004197265	0.007809648
k__Bacteria|p__Synergistetes|c__Synergistia|o__Synergistales|f__Synergistaceae	Butyrat...mol.g.	-0.234265501	0.004291185	0.007942815
k__Bacteria|p__Firmicutes|c__Clostridia|o__SHA-98|365762	Calprotectin...g.g.	-0.233211985	0.004337241	0.007986467
k__Bacteria|p__Firmicutes|c__Clostridia|o__Clostridiales|f__Lachnospiraceae|g__Anaerostipes	Beta.Defensin.2..ng.g.	-0.232127808	0.004526993	0.008292902
k__Bacteria|p__Cyanobacteria|c__4C0d-2|o__YS2	Chromogranin.A..nmol.g.	-0.23046206	0.004833054	0.008808165
k__Bacteria|p__Bacteroidetes|c__Bacteroidia|o__Bacteroidales|f__Prevotellaceae|g__Prevotella	Beta.Defensin.2..ng.g.	0.229725171	0.004974263	0.009019265
k__Bacteria|p__Firmicutes|c__Clostridia|o__Clostridiales|f__Ruminococcaceae|g__Ruminococcus|s__bromii	Beta.Defensin.2..ng.g.	-0.229178685	0.005081359	0.009120383
k__Bacteria|p__Firmicutes|c__Clostridia|o__SHA-98|365762	Acetat...mol.g.	-0.229992808	0.005072729	0.009120383
k__Bacteria|p__Firmicutes|c__Clostridia|o__Clostridiales|f__Ruminococcaceae|g__Faecalibacterium|s__prausnitzii	Calprotectin...g.g.	-0.228564247	0.00520423	0.009272175
k__Bacteria|p__Tenericutes|c__Mollicutes|o__RF39	Acetat...mol.g.	-0.229263824	0.00521811	0.009272175
k__Bacteria|p__Tenericutes|c__Mollicutes	Acetat...mol.g.	-0.228627921	0.005347957	0.009455626
k__Bacteria|p__Firmicutes|c__Clostridia|o__Clostridiales|f__Lachnospiraceae|g__Dorea	Propionat...mol.g.	-0.228089517	0.005460145	0.009558871
k__Bacteria|p__Firmicutes|c__Clostridia|o__Clostridiales|f__Veillonellaceae|g__Acidaminococcus	Butyrat...mol.g.	0.228136138	0.005450348	0.009558871
k__Bacteria|p__Firmicutes|c__Clostridia|o__Clostridiales|f__Peptostreptococcaceae	Chromogranin.A..nmol.g.	0.226623618	0.005609939	0.009747636
k__Bacteria|p__Bacteroidetes|c__Bacteroidia|o__Bacteroidales|f__Porphyromonadaceae|g__Parabacteroides|s__distasonis	Butyrat...mol.g.	-0.227199314	0.005650255	0.009747636
k__Bacteria|p__Firmicutes|c__Erysipelotrichi|o__Erysipelotrichales|f__Erysipelotrichaceae|g__Coprobacillus	Butyrat...mol.g.	-0.227226684	0.005644323	0.009747636
k__Bacteria|p__Firmicutes|c__Clostridia|o__Clostridiales|f__[Veillonellaceae/Clostridiaceae]	Beta.Defensin.2..ng.g.	0.22607849	0.005728873	0.00983552
k__Bacteria|p__Proteobacteria|c__Gammaproteobacteria|o__Aeromonadales|f__Succinivibrionaceae|g__Succinivibrio	Beta.Defensin.2..ng.g.	0.22579553	0.005791492	0.009895222
k__Bacteria|p__Firmicutes|c__Clostridia|o__SHA-98|365762	Chromogranin.A..nmol.g.	-0.224872199	0.006000092	0.010153997
k__Bacteria|p__Proteobacteria|c__Gammaproteobacteria|o__Enterobacteriales|f__Enterobacteriaceae	Calprotectin...g.g.	-0.224988433	0.005973468	0.010153997
k__Bacteria|p__Firmicutes|c__Erysipelotrichi|o__Erysipelotrichales|f__Erysipelotrichaceae|g__Eubacterium|s__biforme	Calprotectin...g.g.	0.224701784	0.006039316	0.010171938
k__Bacteria|p__Synergistetes|c__Synergistia|o__Synergistales|f__Synergistaceae	Propionat...mol.g.	-0.225173173	0.00610515	0.010234317
k__Bacteria|p__Firmicutes|c__Clostridia|o__Clostridiales|f__Lachnospiraceae|g__[Lachnospira/Roseburia]	Butyrat...mol.g.	0.224455299	0.006273987	0.010467969
k__Bacteria|p__Bacteroidetes|c__Bacteroidia|o__Bacteroidales|f__Barnesiellaceae	Valerat...mol.g.	0.223726097	0.006449734	0.010710912
k__Bacteria|p__Firmicutes|c__Bacilli|o__Lactobacillales	Propionat...mol.g.	0.223070534	0.00661146	0.010927016
k__Bacteria|p__Firmicutes|c__Clostridia|o__Clostridiales|f__Lachnospiraceae|g__Coprococcus|s__catus	Butyrat...mol.g.	-0.222950907	0.006641358	0.010927016
k__Bacteria|p__Firmicutes|c__Clostridia|o__Clostridiales|f__Lachnospiraceae|g__[Lachnospira/Roseburia]	Valerat...mol.g.	0.222366031	0.006789273	0.011118904
k__Bacteria|p__Firmicutes|c__Erysipelotrichi|o__Erysipelotrichales|f__Erysipelotrichaceae|g__Catenibacterium	Calprotectin...g.g.	0.220749368	0.007015548	0.011436774
k__Bacteria|p__Tenericutes	Propionat...mol.g.	-0.220467579	0.007289785	0.011829572
k__Bacteria|p__Bacteroidetes|c__Bacteroidia|o__Bacteroidales|f__Bacteroidaceae|g__Bacteroides	Chromogranin.A..nmol.g.	0.219417436	0.007374799	0.0118773
k__Bacteria|p__Bacteroidetes|c__Bacteroidia|o__Bacteroidales|f__Bacteroidaceae|g__Bacteroides|s__plebeius	Propionat...mol.g.	0.220115823	0.007386039	0.0118773
k__Bacteria|p__Firmicutes|c__Clostridia|o__Clostridiales|f__Lachnospiraceae|g__Dorea	Butyrat...mol.g.	-0.219801794	0.007472921	0.011962883
k__Bacteria|p__Bacteroidetes|c__Bacteroidia|o__Bacteroidales|f__Paraprevotellaceae|g__Prevotella	Acetat...mol.g.	0.219236705	0.00763155	0.012162037
k__Bacteria|p__Firmicutes|c__Clostridia|o__Clostridiales|f__Clostridiaceae|g__Clostridium	Butyrat...mol.g.	0.218580124	0.007819609	0.012406104

k__Bacteria|p__Firmicutes|c__Clostridia|o__Clostridiales|f__Ruminococcaceae|g__Ruminococcus	Valerat...mol.g.	-0.218236566	0.007919639	0.012508962
k__Bacteria|p__Proteobacteria|c__Betaproteobacteria|o__Burkholderiales|f__Alcaligenaceae|g__Sutterella	Acetat...mol.g.	0.217918954	0.008013122	0.012545105
k__Bacteria|p__Firmicutes|c__Clostridia|o__Clostridiales|f__Lachnospiraceae|g__[Lachnospira/Roseburia]	Propionat...mol.g.	0.218011478	0.007985789	0.012545105
k__Bacteria|p__Firmicutes|c__Clostridia|o__Clostridiales|f__Lachnospiraceae|g__Dorea|s__formicigenerans	Acetat...mol.g.	-0.217202523	0.008227588	0.012769096
k__Bacteria|p__Firmicutes|c__Clostridia|o__Clostridiales|f__Lachnospiraceae|g__Blautia|s__obeum	Butyrat...mol.g.	-0.21720098	0.008228055	0.012769096
k__Bacteria|p__Firmicutes|c__Clostridia|o__Clostridiales|f__Dehalobacteriaceae|g__Dehalobacterium	Chromogranin.A..nmol.g.	-0.215122411	0.008647026	0.013360951
k__Bacteria|p__Bacteroidetes|c__Bacteroidia|o__Bacteroidales|f__Bacteroidaceae|g__Bacteroides|s__uniformis	Propionat...mol.g.	0.215186021	0.008858768	0.013628868
k__Bacteria|p__Actinobacteria|c__Coriobacteriia|o__Coriobacteriales|f__Coriobacteriaceae	Acetat...mol.g.	-0.214856379	0.008965922	0.013734264
k__Bacteria|p__Firmicutes|c__Clostridia|o__Clostridiales|f__Lachnospiraceae|g__Blautia|s__obeum	Valerat...mol.g.	-0.214359593	0.009129572	0.013865418
k__Bacteria|p__Firmicutes|c__Clostridia|o__Clostridiales|f__[Ruminococcaceae/Lachnospiraceae]	Valerat...mol.g.	0.214463082	0.009095264	0.013865418
k__Bacteria|p__Proteobacteria|c__Alphaproteobacteria|o__RF32	Calprotectin...g.g.	-0.213483856	0.009181333	0.013884693
k__Bacteria|p__Bacteroidetes|c__Bacteroidia|o__Bacteroidales	Beta.Defensin.2..ng.g.	0.212764422	0.009425001	0.014192791
k__Bacteria|p__Synergistetes|c__Synergistia|o__Synergistales|f__Synergistaceae	Acetat...mol.g.	-0.212756533	0.009675784	0.014508959
k__Bacteria|p__Bacteroidetes|c__Bacteroidia|o__Bacteroidales|f__Bacteroidaceae|g__Bacteroides|s__uniformis	Beta.Defensin.2..ng.g.	-0.211610216	0.009827858	0.014554228
k__Bacteria|p__Bacteroidetes|c__Bacteroidia|o__Bacteroidales|f__Bacteroidaceae|g__Bacteroides|s__fragilis	Calprotectin...g.g.	-0.211377942	0.009910741	0.014554228
k__Bacteria|p__Firmicutes|c__Clostridia|o__Clostridiales|f__Peptococcaceae|g__rc4-4	Propionat...mol.g.	-0.212220943	0.009864595	0.014554228
k__Bacteria|p__Tenericutes	Butyrat...mol.g.	-0.212112492	0.009903221	0.014554228
k__Bacteria|p__Bacteroidetes|c__Bacteroidia|o__Bacteroidales|f__Bacteroidaceae|g__Bacteroides|s__coprophilus	Caproat...mol.g.	-0.212182379	0.009878315	0.014554228
k__Bacteria|p__Firmicutes|c__Clostridia|o__Clostridiales|f__Veillonellaceae|g__Phascolarctobacterium	Beta.Defensin.2..ng.g.	-0.210626404	0.010183152	0.014892732
k__Bacteria|p__Proteobacteria|c__Betaproteobacteria|o__Burkholderiales|f__Oxalobacteraceae|g__Oxalobacter|s__formigenes	Caproat...mol.g.	0.210869891	0.010355386	0.015082554
k__Bacteria|p__Bacteroidetes|c__Bacteroidia|o__Bacteroidales|f__Bacteroidaceae|g__Bacteroides|s__ovatus	Valerat...mol.g.	-0.210476776	0.010502172	0.015233913
k__Bacteria|p__Firmicutes|c__Clostridia|o__Clostridiales|f__Ruminococcaceae|g__Ruminococcus|s__bromii	Propionat...mol.g.	-0.210345957	0.010551423	0.015243138
k__Bacteria|p__Bacteroidetes|c__Bacteroidia|o__Bacteroidales|f__Barnesiellaceae	Calprotectin...g.g.	-0.208696901	0.010912972	0.015524708
k__Bacteria|p__Tenericutes|c__RF3|o__ML615J-28	Acetat...mol.g.	-0.209555157	0.0108535	0.015524708
k__Bacteria|p__Bacteroidetes|c__Bacteroidia|o__Bacteroidales|f__Porphyromonadaceae|g__Parabacteroides	Butyrat...mol.g.	-0.209414214	0.01090813	0.015524708
k__Bacteria|p__Firmicutes|c__Clostridia|o__Clostridiales|f__[Ruminococcaceae/[Mogibacteriaceae]]	Valerat...mol.g.	0.209380932	0.010921066	0.015524708
k__Bacteria|p__Firmicutes|c__Erysipelotrichi|o__Erysipelotrichales|f__Erysipelotrichaceae|g__cc_115	Propionat...mol.g.	-0.208945271	0.011091639	0.015668092
k__Bacteria|p__Firmicutes|c__Clostridia|o__Clostridiales|f__Lachnospiraceae|g__Coprococcus	Caproat...mol.g.	0.208898457	0.011110106	0.015668092
k__Bacteria|p__Actinobacteria|c__Coriobacteriia|o__Coriobacteriales|f__Coriobacteriaceae|g__Collinsella	Butyrat...mol.g.	0.208624844	0.011218585	0.0156965
k__Bacteria|p__Firmicutes|c__Erysipelotrichi|o__Erysipelotrichales|f__Erysipelotrichaceae|g__Coprobacillus	Valerat...mol.g.	-0.208630515	0.011216328	0.0156965
k__Bacteria|p__Firmicutes|c__Clostridia|o__Clostridiales|f__Lachnospiraceae|g__Roseburia	Calprotectin...g.g.	0.207450004	0.011408719	0.015837818
k__Bacteria|p__Firmicutes|c__Clostridia|o__Clostridiales|f__[Ruminococcaceae/Clostridiaceae]	Caproat...mol.g.	-0.208259581	0.011364851	0.015837818
k__Bacteria|p__Firmicutes|c__Bacilli|o__Lactobacillales|f__Streptococcaceae	Propionat...mol.g.	0.20794702	0.01149134	0.015890442
k__Bacteria|p__Tenericutes|c__Mollicutes|o__RF39	Butyrat...mol.g.	-0.20764489	0.011614781	0.015998886
k__Bacteria|p__Actinobacteria	Chromogranin.A..nmol.g.	-0.20580935	0.012091098	0.016400719
k__Bacteria|p__Firmicutes|c__Clostridia|o__Clostridiales|f__Lachnospiraceae	Propionat...mol.g.	-0.206608518	0.012047102	0.016400719
k__Bacteria|p__Tenericutes|c__Mollicutes|o__RF39	Propionat...mol.g.	-0.206773715	0.011977259	0.016400719
k__Bacteria|p__Bacteroidetes|c__Bacteroidia|o__Bacteroidales|f__Bacteroidaceae|g__Bacteroides|s__plebeius	Valerat...mol.g.	0.206571342	0.012062869	0.016400719
k__Bacteria|p__Tenericutes|c__Mollicutes	Propionat...mol.g.	-0.206249471	0.012200137	0.0164857
k__Bacteria|p__Firmicutes|c__Erysipelotrichi|o__Erysipelotrichales|f__Erysipelotrichaceae|g__Eubacterium	Calprotectin...g.g.	0.205113273	0.012391261	0.016555118
k__Bacteria	Acetat...mol.g.	0.205812879	0.012388517	0.016555118
k__Bacteria|p__Tenericutes|c__Mollicutes	Butyrat...mol.g.	-0.205973738	0.012318815	0.016555118
k__Archaea|p__Euryarchaeota|c__Methanobacteria|o__Methanobacteriales|f__Methanobacteriaceae|g__Methanobrevibacter	Acetat...mol.g.	-0.20564022	0.012463716	0.016589555
k__Bacteria|p__Actinobacteria|c__Actinobacteria|o__Bifidobacteriales|f__Bifidobacteriaceae|g__Bifidobacterium|s__adolescentis	Acetat...mol.g.	0.205330604	0.012599568	0.016707801
k__Bacteria|p__Bacteroidetes|c__Bacteroidia|o__Bacteroidales|f__Porphyromonadaceae|g__Parabacteroides	Acetat...mol.g.	-0.204763735	0.01285166	0.016978736

k__Bacteria|p__Firmicutes|c__Bacilli|o__Lactobacillales|f__Streptococcaceae|g__Streptococcus	Propionat...mol.g.	0.204462761	0.012987292	0.017094377
k__Bacteria|p__Proteobacteria|c__Betaproteobacteria|o__Burkholderiales|f__Alcaligenaceae|g__Sutterella	Butyrat...mol.g.	0.204241429	0.013087833	0.017163145
k__Bacteria|p__Firmicutes|c__Clostridia|o__Clostridiales|f__Clostridiaceae|g__Clostridium	Propionat...mol.g.	0.203162272	0.013587858	0.017753359
k__Bacteria|p__Actinobacteria|c__Coriobacteriia|o__Coriobacteriales|f__Coriobacteriaceae|g__Collinsella	Acetat...mol.g.	0.202984171	0.013671967	0.017797819
k__Bacteria|p__Firmicutes|c__Clostridia|o__Clostridiales|f__Dehalobacteriaceae|g__Dehalobacterium	Propionat...mol.g.	-0.202449908	0.013927011	0.017958993
k__Bacteria|p__Actinobacteria|c__Coriobacteriia|o__Coriobacteriales|f__Coriobacteriaceae|g__Eggerthella|s__lenta	Valerat...mol.g.	-0.202407608	0.01394738	0.017958993
k__Bacteria|p__Firmicutes|c__Erysipelotrichi|o__Erysipelotrichales|f__Erysipelotrichaceae|g__Coprobacillus	Caproat...mol.g.	-0.202476632	0.013914155	0.017958993
k__Bacteria|p__Firmicutes|c__Clostridia|o__Clostridiales|f__Lachnospiraceae|g__Coprococcus|s__eutactus	Caproat...mol.g.	0.202095069	0.01409869	0.018088286
k__Bacteria|p__Bacteroidetes|c__Bacteroidia|o__Bacteroidales|f__Bacteroidaceae|g__Bacteroides|s__coprophilus	Butyrat...mol.g.	0.201406965	0.014436884	0.018455555
k__Bacteria|p__Firmicutes|c__Clostridia|o__Clostridiales|f__Lachnospiraceae|g__Blautia|s__obeum	Beta.Defensin.2..ng.g.	-0.20057309	0.014514169	0.01848785
k__Bacteria|p__Bacteroidetes|c__Bacteroidia|o__Bacteroidales|f__Prevotellaceae|g__Prevotella|s__copri	Beta.Defensin.2..ng.g.	0.199844805	0.014882711	0.018889586
k__Bacteria|p__Proteobacteria|c__Betaproteobacteria|o__Burkholderiales|f__Oxalobacteraceae|g__Oxalobacter|s__formigenes	Propionat...mol.g.	-0.199318438	0.015507093	0.019612028
k__Bacteria|p__Firmicutes|c__Bacilli	Propionat...mol.g.	0.19895721	0.01569904	0.019784379
k__Bacteria|p__Proteobacteria|c__Betaproteobacteria|o__Burkholderiales	Acetat...mol.g.	0.198679394	0.015848068	0.019901615
k__Bacteria|p__Firmicutes|c__Erysipelotrichi|o__Erysipelotrichales|f__Erysipelotrichaceae	Calprotectin...g.g.	0.197750734	0.015988222	0.019936722
k__Bacteria|p__Bacteroidetes|c__Bacteroidia|o__Bacteroidales|f__Bacteroidaceae|g__Bacteroides|s__eggerthii	Valerat...mol.g.	0.198439173	0.01597792	0.019936722
k__Bacteria|p__Firmicutes|c__Clostridia|o__Clostridiales|f__[Veillonellaceae/Lachnospiraceae]	Butyrat...mol.g.	0.19795307	0.016243518	0.020184244
k__Bacteria|p__Tenericutes|c__Mollicutes	Beta.Defensin.2..ng.g.	0.196446309	0.016712406	0.020694527
k__Bacteria|p__Tenericutes|c__Mollicutes|o__RF39	Beta.Defensin.2..ng.g.	0.195778711	0.017093945	0.021093481
k__Bacteria|p__Tenericutes|c__RF3|o__ML615J-28	Propionat...mol.g.	-0.194692686	0.018126183	0.022223812
k__Bacteria|p__Firmicutes|c__Erysipelotrichi|o__Erysipelotrichales|f__Erysipelotrichaceae|g__Catenibacterium	Butyrat...mol.g.	0.194678083	0.018135023	0.022223812
k__Archaea|p__Euryarchaeota|c__Methanobacteria|o__Methanobacteriales|f__Methanobacteriaceae|g__Methanobrevibacter	Propionat...mol.g.	-0.193877814	0.018625256	0.022668241
k__Bacteria	Propionat...mol.g.	0.193952531	0.018579004	0.022668241
k__Bacteria|p__Bacteroidetes|c__Bacteroidia|o__Bacteroidales|f__Bacteroidaceae|g__Bacteroides|s__plebeius	Butyrat...mol.g.	0.193496207	0.018863035	0.022879281
k__Bacteria|p__Firmicutes|c__Clostridia|o__Clostridiales|f__Lachnospiraceae|g__Lachnospira	Calprotectin...g.g.	0.192525769	0.019063166	0.022965263
k__Bacteria|p__Firmicutes|c__Clostridia|o__Clostridiales|f__[Veillonellaceae/Clostridiaceae]	Propionat...mol.g.	0.193276708	0.019000991	0.022965263
k__Bacteria|p__Bacteroidetes|c__Bacteroidia|o__Bacteroidales|f__Paraprevotellaceae|g__Prevotella	Beta.Defensin.2..ng.g.	0.192085737	0.019344066	0.023224934
k__Bacteria|p__Actinobacteria|c__Coriobacteriia|o__Coriobacteriales|f__Coriobacteriaceae|g__Slackia	Valerat...mol.g.	0.192633208	0.019410477	0.023226201
k__Bacteria|p__Tenericutes	Beta.Defensin.2..ng.g.	0.191392235	0.019794039	0.023605684
k__Bacteria|p__Firmicutes|c__Clostridia|o__Clostridiales|f__Lachnospiraceae|g__Anaerostipes	Valerat...mol.g.	-0.191362821	0.02024132	0.024058363
k__Bacteria|p__Firmicutes|c__Erysipelotrichi|o__Erysipelotrichales|f__Erysipelotrichaceae|g__Coprobacillus	Propionat...mol.g.	-0.190567592	0.020776887	0.024612609
k__Bacteria|p__Bacteroidetes|c__Bacteroidia|o__Bacteroidales|f__Porphyromonadaceae|g__Parabacteroides	Propionat...mol.g.	-0.189390669	0.021591931	0.025493145
k__Bacteria|p__Firmicutes|c__Clostridia|o__Clostridiales|f__[Veillonellaceae/Clostridiaceae]	Chromogranin.A..nmol.g.	0.18815547	0.022015786	0.025652682
k__Bacteria|p__Actinobacteria|c__Coriobacteriia|o__Coriobacteriales|f__Coriobacteriaceae|g__Slackia	Beta.Defensin.2..ng.g.	0.188339221	0.021884141	0.025652682
k__Bacteria|p__Bacteroidetes|c__Bacteroidia|o__Bacteroidales|f__Bacteroidaceae|g__Bacteroides|s__eggerthii	Beta.Defensin.2..ng.g.	0.188194093	0.021988059	0.025652682
k__Bacteria|p__Bacteroidetes|c__Bacteroidia|o__Bacteroidales|f__Bacteroidaceae|g__Bacteroides|s__plebeius	Acetat...mol.g.	0.188850909	0.021974847	0.025652682
k__Bacteria|p__Proteobacteria|c__Betaproteobacteria|o__Burkholderiales	Butyrat...mol.g.	0.188417063	0.022286848	0.025883659
k__Bacteria|p__Firmicutes|c__Clostridia|o__Clostridiales|f__[Ruminococcaceae/Clostridiaceae]	Valerat...mol.g.	-0.188153666	0.022478125	0.02602077
k__Bacteria|p__Firmicutes|c__Clostridia|o__Clostridiales|f__Veillonellaceae|g__Mitsuokella	Beta.Defensin.2..ng.g.	0.187095533	0.022788543	0.026294461
k__Bacteria|p__Firmicutes|c__Clostridia|o__Clostridiales|f__Ruminococcaceae|g__Ruminococcus	Acetat...mol.g.	-0.187092637	0.023263004	0.026668743
k__Bacteria|p__Lentisphaerae|c__Lentisphaeria|o__Victivallales|f__Victivallaceae	Caproat...mol.g.	0.187166959	0.023207269	0.026668743
k__Bacteria|p__Firmicutes|c__Clostridia|o__Clostridiales|f__Lachnospiraceae	Calprotectin...g.g.	0.186235145	0.023432857	0.026765061
k__Bacteria|p__Firmicutes|c__Clostridia|o__Clostridiales|f__Ruminococcaceae	Calprotectin...g.g.	-0.186149755	0.023497649	0.026765061
k__Bacteria|p__Bacteroidetes|c__Bacteroidia|o__Bacteroidales|f__Odoribacteraceae|g__Odoribacter	Butyrat...mol.g.	0.186492623	0.023717192	0.026928822
k__Bacteria|p__Firmicutes|c__Clostridia|o__Clostridiales|f__[Ruminococcaceae/Lachnospiraceae]	Beta.Defensin.2..ng.g.	0.185369014	0.024097217	0.027273173

k__Bacteria|p__Firmicutes|c__Clostridia|o__Clostridiales|f__Lachnospiraceae|g__Ruminococcus|s__gnavus	Valerat...mol.g.	-0.185776664	0.024269088	0.027380497
k__Bacteria|p__Firmicutes|c__Clostridia|o__Clostridiales|f__Lachnospiraceae|g__Blautia	Acetat...mol.g.	-0.185120295	0.024784693	0.027873717
k__Bacteria|p__Bacteroidetes|c__Bacteroidia|o__Bacteroidales|f__Bacteroidaceae|g__Bacteroides|s__uniformis	Acetat...mol.g.	0.184325738	0.025421386	0.028499576
k__Bacteria|p__Actinobacteria|c__Actinobacteria|o__Bifidobacteriales|f__Bifidobacteriaceae|g__Bifidobacterium|s__adolescentis	Propionat...mol.g.	0.183734341	0.02590433	0.028949673
k__Bacteria|p__Firmicutes|c__Clostridia|o__Clostridiales|f__Ruminococcaceae	Beta.Defensin.2..ng.g.	-0.181897773	0.026924451	0.029995396
k__Bacteria|p__Firmicutes|c__Clostridia|o__Clostridiales|f__Ruminococcaceae|g__Ruminococcus|s__bromii	Valerat...mol.g.	-0.182372349	0.027046493	0.030037197
k__Bacteria|p__Tenericutes|c__RF3|o__ML615J-28	Butyrat...mol.g.	-0.18225752	0.027144725	0.030052378
k__Bacteria|p__Bacteroidetes|c__Bacteroidia|o__Bacteroidales|f__Porphyromonadaceae|g__Parabacteroides|s__gordonii	Butyrat...mol.g.	-0.181991211	0.027373716	0.03021178
k__Bacteria|p__Firmicutes|c__Clostridia|o__Clostridiales|f__Ruminococcaceae|g__Faecalibacterium|s__prausnitzii	Acetat...mol.g.	0.181828142	0.027514746	0.030273415
k__Bacteria|p__Firmicutes|c__Clostridia|o__Clostridiales|f__[Veillonellaceae/Lachnospiraceae]	Caproat...mol.g.	-0.180334573	0.028835567	0.031628742
k__Bacteria|p__Bacteroidetes|c__Bacteroidia|o__Bacteroidales|f__Paraprevotellaceae|g__Prevotella	Propionat...mol.g.	0.179911044	0.0292198	0.031951578
k__Bacteria|p__Bacteroidetes|c__Bacteroidia|o__Bacteroidales|f__Porphyromonadaceae|g__Parabacteroides|s__gordonii	Acetat...mol.g.	-0.179753759	0.029363598	0.032010326
k__Bacteria|p__Firmicutes|c__Erysipelotrichi|o__Erysipelotrichales|f__Erysipelotrichaceae|g__Catenibacterium	Beta.Defensin.2..ng.g.	0.178518284	0.029944541	0.032444587
k__Bacteria|p__Lentisphaerae|c__Lentisphaeria|o__Victivallales|f__Victivallaceae	Beta.Defensin.2..ng.g.	0.178547147	0.029917576	0.032444587
k__Bacteria|p__Firmicutes|c__Clostridia|o__Clostridiales|f__Lachnospiraceae|g__Roseburia|s__faecis	Calprotectin...g.g.	0.178345381	0.030106513	0.032520932
k__Bacteria|p__Bacteroidetes|c__Bacteroidia|o__Bacteroidales|f__Barnesiellaceae	Beta.Defensin.2..ng.g.	0.177922731	0.030505579	0.032668337
k__Bacteria|p__Bacteroidetes|c__Bacteroidia|o__Bacteroidales|f__Paraprevotellaceae	Beta.Defensin.2..ng.g.	0.17792571	0.030502751	0.032668337
k__Bacteria|p__Bacteroidetes|c__Bacteroidia|o__Bacteroidales|f__Porphyromonadaceae|g__Parabacteroides|s__distasonis	Valerat...mol.g.	-0.178513508	0.030518745	0.032668337
k__Bacteria|p__Firmicutes|c__Bacilli|o__Lactobacillales|f__Streptococcaceae|g__[Streptococcus/Lactococcus]	Caproat...mol.g.	0.177992023	0.031015859	0.033100763
k__Bacteria|p__Firmicutes|c__Clostridia|o__Clostridiales|f__Veillonellaceae|g__Dialister	Valerat...mol.g.	0.177415463	0.031573462	0.033594963
k__Bacteria|p__Firmicutes|c__Clostridia|o__Clostridiales|f__Veillonellaceae|g__Dialister	Beta.Defensin.2..ng.g.	0.175205547	0.033180004	0.034990082
k__Bacteria|p__Firmicutes|c__Clostridia|o__Clostridiales|f__Lachnospiraceae|g__Anaerostipes	Acetat...mol.g.	-0.175925228	0.033054253	0.034990082
k__Bacteria|p__Firmicutes|c__Clostridia|o__Clostridiales|f__Lachnospiraceae|g__Anaerostipes	Propionat...mol.g.	-0.175837413	0.033143316	0.034990082
k__Bacteria|p__Firmicutes|c__Clostridia|o__Clostridiales|f__Veillonellaceae|g__Acidaminococcus	Beta.Defensin.2..ng.g.	0.174439136	0.033969328	0.035662412
k__Bacteria|p__Firmicutes|c__Clostridia|o__Clostridiales|f__Christensenellaceae	Calprotectin...g.g.	-0.174392131	0.034018251	0.035662412
k__Bacteria|p__Bacteroidetes|c__Bacteroidia|o__Bacteroidales|f__Bacteroidaceae|g__Bacteroides|s__caccae	Calprotectin...g.g.	-0.17384517	0.034591927	0.036157156
k__Bacteria|p__Bacteroidetes|c__Bacteroidia|o__Bacteroidales|f__Bacteroidaceae|g__Bacteroides|s__ovatus	Calprotectin...g.g.	0.172064103	0.036516974	0.038057375
k__Bacteria|p__Firmicutes|c__Clostridia|o__Clostridiales|f__Veillonellaceae|g__Mitsuokella	Calprotectin...g.g.	0.171096257	0.03760042	0.039071944
k__Bacteria|p__Actinobacteria|c__Coriobacteriia|o__Coriobacteriales|f__Coriobacteriaceae	Chromogranin.A..nmol.g.	-0.170798883	0.0379387	0.039308526
k__Bacteria|p__Firmicutes|c__Clostridia|o__Clostridiales|f__Ruminococcaceae	Caproat...mol.g.	-0.170887701	0.038502605	0.039776824
k__Bacteria|p__Proteobacteria|c__Gammaproteobacteria|o__Aeromonadales|f__Succinivibrionaceae|g__Succinivibrio	Chromogranin.A..nmol.g.	0.169825295	0.039064189	0.040123715
k__Bacteria|p__Firmicutes|c__Clostridia|o__Clostridiales|f__[Ruminococcaceae/[Mogibacteriaceae]]	Butyrat...mol.g.	0.170494116	0.03895839	0.040123715
k__Bacteria|p__Actinobacteria	Acetat...mol.g.	-0.169512208	0.040115282	0.041084576
k__Bacteria|p__Firmicutes|c__Clostridia|o__Clostridiales|f__Ruminococcaceae|g__Oscillospira	Valerat...mol.g.	-0.169032842	0.040690468	0.041553908
k__Bacteria|p__Tenericutes|c__Mollicutes|o__Anaeroplasmatales|f__Anaeroplasmataceae	Caproat...mol.g.	0.168392049	0.041470139	0.042228776
k__Bacteria|p__Tenericutes|c__Mollicutes|o__Anaeroplasmatales|f__Anaeroplasmataceae	Chromogranin.A..nmol.g.	-0.166415592	0.043229509	0.043786647
k__Bacteria|p__Actinobacteria|c__Coriobacteriia|o__Coriobacteriales|f__Coriobacteriaceae|g__Eggerthella|s__lenta	Beta.Defensin.2..ng.g.	-0.166321962	0.043348934	0.043786647
k__Bacteria|p__Firmicutes|c__Clostridia|o__Clostridiales|f__Peptococcaceae|g__rc4-4	Calprotectin...g.g.	-0.166305742	0.043369651	0.043786647
k__Bacteria|p__Firmicutes|c__Clostridia|o__Clostridiales|f__[Veillonellaceae/Clostridiaceae]	Butyrat...mol.g.	0.166574778	0.043749642	0.044045164
k__Bacteria|p__Firmicutes|c__Clostridia|o__Clostridiales|f__Peptococcaceae|g__rc4-4	Beta.Defensin.2..ng.g.	-0.165351425	0.04460319	0.044737776
k__Bacteria|p__Actinobacteria|c__Coriobacteriia|o__Coriobacteriales|f__Coriobacteriaceae	Caproat...mol.g.	0.165848584	0.044689378	0.044737776
k__Bacteria|p__Bacteroidetes|c__Bacteroidia|o__Bacteroidales|f__Paraprevotellaceae|g__Prevotella	Chromogranin.A..nmol.g.	-0.16359731	0.046946957	0.046710497
k__Bacteria|p__Bacteroidetes|c__Bacteroidia|o__Bacteroidales|f__Odoribacteraceae	Beta.Defensin.2..ng.g.	0.163613965	0.046924231	0.046710497
k__Bacteria|p__Bacteroidetes|c__Bacteroidia|o__Bacteroidales|f__Porphyromonadaceae|g__Parabacteroides|s__distasonis	Calprotectin...g.g.	-0.163497688	0.047083088	0.046710497
k__Bacteria|p__Firmicutes|c__Clostridia|o__Clostridiales|f__Clostridiaceae|g__Clostridium	Acetat...mol.g.	0.163952128	0.047223235	0.046710497

k__Bacteria|p__Firmicutes|c__Erysipelotrichi|o__Erysipelotrichales|f__Erysipelotrichaceae	Valerat...mol.g.	-0.163883484	0.047317148	0.046710497
k__Bacteria|p__Firmicutes|c__Clostridia|o__Clostridiales|f__Lachnospiraceae|g__Lachnospira	Valerat...mol.g.	-0.161798931	0.050243937	0.049462366
k__Bacteria|p__Bacteroidetes|c__Bacteroidia|o__Bacteroidales|f__Odoribacteraceae	Calprotectin...g.g.	0.160829065	0.050853805	0.049727182
k__Bacteria|p__Firmicutes|c__Clostridia|o__Clostridiales|f__Ruminococcaceae|g__Ruminococcus|s__bromii	Butyrat...mol.g.	-0.161323063	0.050932713	0.049727182
k__Bacteria|p__Firmicutes|c__Erysipelotrichi|o__Erysipelotrichales|f__Erysipelotrichaceae|g__Eubacterium|s__biforme	Caproat...mol.g.	0.161495661	0.050681995	0.049727182
