# Supplementary material for: The influence of a short-term gluten-free diet on the human gut microbiome
Source: Genome Med. 2016 Apr 21;8:45. doi: 10.1186/s13073-016-0295-y (PMC4841035; doi:10.1186/s13073-016-0295-y)
Supplement: Additional file 9: Table S4. — Correlation of predicted HUMAnN pathway activity and levels of fecal biomarkers. (PDF 228 kb) [file 13073_2016_295_MOESM9_ESM.pdf]

Kegg_Pathway	Fecal_Biomarker	Cor(rho)	P-val	Q-val
ko00380_Tryptophan_metabolism	Chromogranin.A..nmol.g.	-0.590626487	2.76E-15	1.30E-12
ko00620_Pyruvate_metabolism	Propionat...mol.g.	-0.545109725	9.44E-13	1.48E-10
ko00253_Tetracycline_biosynthesis	Propionat...mol.g.	-0.548387066	6.48E-13	1.48E-10
ko00680_Methane_metabolism	Propionat...mol.g.	-0.535245588	2.86E-12	3.36E-10
ko00500_Starch_and_sucrose_metabolism	Chromogranin.A..nmol.g.	0.523955307	8.27E-12	7.77E-10
ko00630_Glyoxylate_and_dicarboxylate_metabolism	Caproat...mol.g.	-0.504836027	7.02E-11	5.49E-09
ko00362_Benzoate_degradation	Propionat...mol.g.	-0.50247784	8.88E-11	5.96E-09
ko00620_Pyruvate_metabolism	Butyrat...mol.g.	-0.498189429	1.36E-10	7.10E-09
ko00670_One_carbon_pool_by_folate	Caproat...mol.g.	0.498148707	1.36E-10	7.10E-09
ko00791_Atrazine_degradation	Chromogranin.A..nmol.g.	-0.492550905	2.03E-10	9.55E-09
ko00903_Limonene_and_pinene_degradation	Chromogranin.A..nmol.g.	-0.490890615	2.39E-10	1.02E-08
ko00290_Valine_leucine_and_isoleucine_biosynthesis	Propionat...mol.g.	-0.48957626	3.12E-10	1.16E-08
ko00130_Ubiquinone_and_other_terpenoid<>quinone_biosynthesis	Valerat...mol.g.	0.489294361	3.20E-10	1.16E-08
ko00253_Tetracycline_biosynthesis	Butyrat...mol.g.	-0.485922503	4.41E-10	1.48E-08
ko00640_Propanoate_metabolism	Propionat...mol.g.	-0.48290635	5.85E-10	1.83E-08
ko00540_Lipopolysaccharide_biosynthesis	Valerat...mol.g.	0.4815342	6.64E-10	1.95E-08
ko00620_Pyruvate_metabolism	Acetat...mol.g.	-0.480170307	7.54E-10	2.08E-08
ko05010_Alzheimer's_disease	Chromogranin.A..nmol.g.	-0.476597682	9.17E-10	2.27E-08
ko00790_Folate_biosynthesis	Valerat...mol.g.	0.478543856	8.76E-10	2.27E-08
ko00061_Fatty_acid_biosynthesis	Propionat...mol.g.	-0.476790806	1.03E-09	2.42E-08
ko00310_Lysine_degradation	Chromogranin.A..nmol.g.	-0.474787466	1.08E-09	2.42E-08
ko00640_Propanoate_metabolism	Acetat...mol.g.	-0.470565012	1.81E-09	3.86E-08
ko00633_Nitrotoluene_degradation	Propionat...mol.g.	-0.468106367	2.25E-09	4.60E-08
ko03010_Ribosome	Caproat...mol.g.	0.46736397	2.40E-09	4.71E-08
ko00510_N<>Glycan_biosynthesis	Valerat...mol.g.	0.465418833	2.86E-09	5.37E-08
ko04974_Protein_digestion_and_absorption	Propionat...mol.g.	0.464028546	3.23E-09	5.83E-08
ko00540_Lipopolysaccharide_biosynthesis	Propionat...mol.g.	0.463138409	3.49E-09	6.07E-08
ko04146_Peroxisome	Valerat...mol.g.	0.459489148	4.79E-09	8.03E-08
ko00030_Pentose_phosphate_pathway	Valerat...mol.g.	-0.45909434	4.96E-09	8.03E-08
ko00680_Methane_metabolism	Acetat...mol.g.	-0.45833597	5.30E-09	8.29E-08
ko00253_Tetracycline_biosynthesis	Acetat...mol.g.	-0.455075648	7.00E-09	9.67E-08
ko00622_Xylene_degradation	Propionat...mol.g.	-0.455082004	7.00E-09	9.67E-08
ko00640_Propanoate_metabolism	Butyrat...mol.g.	-0.455462975	6.78E-09	9.67E-08
ko00240_Pyrimidine_metabolism	Caproat...mol.g.	0.456153541	6.39E-09	9.67E-08
ko03060_Protein_export	Chromogranin.A..nmol.g.	-0.452539208	7.72E-09	9.80E-08
ko00621_Dioxin_degradation	Propionat...mol.g.	-0.454477538	7.37E-09	9.80E-08
ko04141_Protein_processing_in_endoplasmic_reticulum	Valerat...mol.g.	0.453982608	7.69E-09	9.80E-08
ko00660_C5<>Branched_dibasic_acid_metabolism	Propionat...mol.g.	-0.452732141	8.55E-09	1.06E-07
ko00052_Galactose_metabolism	Chromogranin.A..nmol.g.	0.448659644	1.07E-08	1.29E-07
ko03440_Homologous_recombination	Caproat...mol.g.	0.44914154	1.16E-08	1.36E-07
ko00920_Sulfur_metabolism	Chromogranin.A..nmol.g.	0.44632006	1.30E-08	1.48E-07
ko00061_Fatty_acid_biosynthesis	Butyrat...mol.g.	-0.447509398	1.32E-08	1.48E-07
ko00130_Ubiquinone_and_other_terpenoid<>quinone_biosynthesis	Propionat...mol.g.	0.446305907	1.46E-08	1.60E-07
ko00630_Glyoxylate_and_dicarboxylate_metabolism	Chromogranin.A..nmol.g.	0.443902737	1.60E-08	1.70E-07
ko00900_Terpenoid_backbone_biosynthesis	Caproat...mol.g.	0.444986584	1.63E-08	1.70E-07
ko00625_Chloroalkane_and_chloroalkene_degradation	Butyrat...mol.g.	-0.444563659	1.69E-08	1.73E-07
ko00330_Arginine_and_proline_metabolism	Valerat...mol.g.	-0.443674138	1.82E-08	1.82E-07
ko03030_DNA_replication	Chromogranin.A..nmol.g.	-0.439973198	2.20E-08	2.16E-07
ko00650_Butanoate_metabolism	Propionat...mol.g.	-0.440873265	2.29E-08	2.19E-07
ko00040_Pentose_and_glucuronate_interconversions	Beta.Defensin.2..ng.g.	-0.438944618	2.40E-08	2.21E-07
ko00625_Chloroalkane_and_chloroalkene_degradation	Acetat...mol.g.	-0.440481644	2.36E-08	2.21E-07
ko04122_Sulfur_relay_system	Valerat...mol.g.	-0.438768311	2.72E-08	2.45E-07
ko00250_Alanine_aspartate_and_glutamate_metabolism	Chromogranin.A..nmol.g.	0.435142656	3.26E-08	2.79E-07
ko00630_Glyoxylate_and_dicarboxylate_metabolism	Valerat...mol.g.	-0.436890609	3.16E-08	2.79E-07
ko03030_DNA_replication	Caproat...mol.g.	0.436681302	3.21E-08	2.79E-07
ko05012_Parkinson's_disease	Chromogranin.A..nmol.g.	-0.434434645	3.45E-08	2.90E-07
ko00908_Zeatin_biosynthesis	Propionat...mol.g.	0.433952124	4.00E-08	3.29E-07
ko00460_Cyanoamino_acid_metabolism	Chromogranin.A..nmol.g.	0.431581052	4.34E-08	3.45E-07
ko04910_Insulin_signaling_pathway	Valerat...mol.g.	-0.433099314	4.28E-08	3.45E-07
ko00290_Valine_leucine_and_isoleucine_biosynthesis	Valerat...mol.g.	-0.429551704	5.66E-08	4.36E-07
ko00040_Pentose_and_glucuronate_interconversions	Caproat...mol.g.	-0.429744482	5.58E-08	4.36E-07
ko00625_Chloroalkane_and_chloroalkene_degradation	Propionat...mol.g.	-0.429286399	5.78E-08	4.38E-07
ko03018_RNA_degradation	Valerat...mol.g.	0.428809313	6.00E-08	4.47E-07
ko03010_Ribosome	Chromogranin.A..nmol.g.	-0.426033794	6.72E-08	4.92E-07
ko00680_Methane_metabolism	Butyrat...mol.g.	-0.4272022	6.80E-08	4.92E-07
ko00908_Zeatin_biosynthesis	Valerat...mol.g.	0.426451796	7.21E-08	5.13E-07

ko03060_Protein_export	Caproat...mol.g.	0.425625864	7.69E-08	5.39E-07
ko00020_Citrate_cycle_[TCA_cycle]	Valerat...mol.g.	0.423943556	8.76E-08	6.05E-07
ko02020_Two<>component_system	Valerat...mol.g.	-0.421470407	1.06E-07	7.21E-07
ko04112_Cell_cycle_<>_Caulobacter	Caproat...mol.g.	0.420926972	1.10E-07	7.41E-07
ko00670_One_carbon_pool_by_folate	Valerat...mol.g.	0.418676523	1.31E-07	8.67E-07
ko03420_Nucleotide_excision_repair	Caproat...mol.g.	0.418450886	1.33E-07	8.70E-07
ko00621_Dioxin_degradation	Butyrat...mol.g.	-0.417474041	1.44E-07	9.24E-07
ko00230_Purine_metabolism	Caproat...mol.g.	0.416838596	1.51E-07	9.56E-07
ko00520_Amino_sugar_and_nucleotide_sugar_metabolism	Chromogranin.A..nmol.g.	0.414432507	1.64E-07	1.02E-06
ko00660_C5<>Branched_dibasic_acid_metabolism	Valerat...mol.g.	-0.414101277	1.85E-07	1.14E-06
ko03430_Mismatch_repair	Caproat...mol.g.	0.413338053	1.96E-07	1.19E-06
ko00622_Xylene_degradation	Butyrat...mol.g.	-0.413148316	1.99E-07	1.20E-06
ko00591_Linoleic_acid_metabolism	Caproat...mol.g.	-0.411243776	2.29E-07	1.36E-06
ko04910_Insulin_signaling_pathway	Chromogranin.A..nmol.g.	0.409562309	2.36E-07	1.37E-06
ko04115_p53_signaling_pathway	Chromogranin.A..nmol.g.	-0.409565904	2.35E-07	1.37E-06
ko05145_Toxoplasmosis	Chromogranin.A..nmol.g.	-0.409185329	2.42E-07	1.39E-06
ko00565_Ether_lipid_metabolism	Chromogranin.A..nmol.g.	0.408709486	2.51E-07	1.42E-06
ko03060_Protein_export	Valerat...mol.g.	0.408419057	2.82E-07	1.57E-06
ko00040_Pentose_and_glucuronate_interconversions	Valerat...mol.g.	-0.408320827	2.84E-07	1.57E-06
ko00561_Glycerolipid_metabolism	Propionat...mol.g.	-0.40735749	3.04E-07	1.66E-06
ko00920_Sulfur_metabolism	Caproat...mol.g.	-0.406495741	3.24E-07	1.75E-06
ko00780_Biotin_metabolism	Propionat...mol.g.	0.406167447	3.32E-07	1.77E-06
ko00510_N<>Glycan_biosynthesis	Propionat...mol.g.	0.405496867	3.49E-07	1.84E-06
ko00300_Lysine_biosynthesis	Propionat...mol.g.	-0.404850843	3.65E-07	1.91E-06
ko03030_DNA_replication	Beta.Defensin.2..ng.g.	0.402854554	3.85E-07	1.99E-06
ko00790_Folate_biosynthesis	Beta.Defensin.2..ng.g.	0.40155703	4.23E-07	2.14E-06
ko00780_Biotin_metabolism	Butyrat...mol.g.	0.402934693	4.20E-07	2.14E-06
ko00900_Terpenoid_backbone_biosynthesis	Beta.Defensin.2..ng.g.	0.400439048	4.59E-07	2.29E-06
ko00230_Purine_metabolism	Beta.Defensin.2..ng.g.	0.399526524	4.90E-07	2.42E-06
ko00760_Nicotinate_and_nicotinamide_metabolism	Valerat...mol.g.	0.40058088	4.97E-07	2.43E-06
ko00330_Arginine_and_proline_metabolism	Propionat...mol.g.	-0.400137894	5.13E-07	2.48E-06
ko00480_Glutathione_metabolism	Valerat...mol.g.	0.398197482	5.89E-07	2.82E-06
ko00362_Benzoate_degradation	Butyrat...mol.g.	-0.396583999	6.60E-07	3.13E-06
ko04974_Protein_digestion_and_absorption	Valerat...mol.g.	0.396312598	6.73E-07	3.16E-06
ko00760_Nicotinate_and_nicotinamide_metabolism	Beta.Defensin.2..ng.g.	0.394542247	6.98E-07	3.25E-06
ko00230_Purine_metabolism	Valerat...mol.g.	0.395329925	7.21E-07	3.32E-06
ko00660_C5<>Branched_dibasic_acid_metabolism	Acetat...mol.g.	-0.394115553	7.85E-07	3.58E-06
ko00790_Folate_biosynthesis	Caproat...mol.g.	0.393969772	7.93E-07	3.58E-06
ko00480_Glutathione_metabolism	Propionat...mol.g.	0.393732439	8.07E-07	3.61E-06
ko00471_D<>Glutamine_and_D<>glutamate_metabolism	Caproat...mol.g.	0.393502907	8.20E-07	3.63E-06
ko00590_Arachidonic_acid_metabolism	Valerat...mol.g.	0.393057415	8.45E-07	3.68E-06
ko04141_Protein_processing_in_endoplasmic_reticulum	Caproat...mol.g.	0.393124879	8.42E-07	3.68E-06
ko00061_Fatty_acid_biosynthesis	Acetat...mol.g.	-0.39202334	9.09E-07	3.89E-06
ko03013_RNA_transport	Valerat...mol.g.	-0.391976886	9.12E-07	3.89E-06
ko00760_Nicotinate_and_nicotinamide_metabolism	Caproat...mol.g.	0.390322168	1.02E-06	4.33E-06
ko03440_Homologous_recombination	Beta.Defensin.2..ng.g.	0.388743177	1.05E-06	4.39E-06
ko00970_Aminoacyl<>tRNA_biosynthesis	Caproat...mol.g.	0.389732075	1.06E-06	4.43E-06
ko00630_Glyoxylate_and_dicarboxylate_metabolism	Beta.Defensin.2..ng.g.	-0.387587817	1.13E-06	4.59E-06
ko00621_Dioxin_degradation	Acetat...mol.g.	-0.388996506	1.12E-06	4.59E-06
ko00010_Glycolysis_/_Gluconeogenesis	Propionat...mol.g.	-0.388704033	1.14E-06	4.59E-06
ko00281_Geraniol_degradation	Valerat...mol.g.	0.388782526	1.14E-06	4.59E-06
ko00622_Xylene_degradation	Acetat...mol.g.	-0.387347456	1.25E-06	4.99E-06
ko04210_Apoptosis	Chromogranin.A..nmol.g.	-0.385985662	1.27E-06	5.00E-06
ko04122_Sulfur_relay_system	Caproat...mol.g.	-0.385810031	1.39E-06	5.45E-06
ko00780_Biotin_metabolism	Acetat...mol.g.	0.384013464	1.57E-06	6.11E-06
ko00790_Folate_biosynthesis	Propionat...mol.g.	0.382353358	1.76E-06	6.78E-06
ko00071_Fatty_acid_metabolism	Propionat...mol.g.	-0.381074534	1.92E-06	7.27E-06
ko00561_Glycerolipid_metabolism	Valerat...mol.g.	-0.381126263	1.91E-06	7.27E-06
ko00670_One_carbon_pool_by_folate	Beta.Defensin.2..ng.g.	0.379045625	2.03E-06	7.62E-06
ko05110_Vibrio_cholerae_infection	Chromogranin.A..nmol.g.	-0.378121649	2.16E-06	7.92E-06
ko03060_Protein_export	Beta.Defensin.2..ng.g.	0.378105337	2.16E-06	7.92E-06
ko00520_Amino_sugar_and_nucleotide_sugar_metabolism	Calprotectin...g.g.	0.378294284	2.13E-06	7.92E-06
ko04976_Bile_secretion	Chromogranin.A..nmol.g.	-0.377769999	2.21E-06	7.98E-06
ko05146_Amoebiasis	Chromogranin.A..nmol.g.	-0.377646854	2.23E-06	7.98E-06
ko00362_Benzoate_degradation	Valerat...mol.g.	-0.378951982	2.21E-06	7.98E-06
ko00740_Riboflavin_metabolism	Acetat...mol.g.	0.378106727	2.34E-06	8.32E-06
ko03070_Bacterial_secretion_system	Chromogranin.A..nmol.g.	-0.376493475	2.40E-06	8.42E-06

ko03430_Mismatch_repair	Beta.Defensin.2..ng.g.	0.376598653	2.39E-06	8.42E-06
ko00940_Phenylpropanoid_biosynthesis	Chromogranin.A..nmol.g.	0.376265809	2.44E-06	8.49E-06
ko03440_Homologous_recombination	Chromogranin.A..nmol.g.	-0.37550172	2.57E-06	8.87E-06
ko00290_Valine_leucine_and_isoleucine_biosynthesis	Acetat...mol.g.	-0.376370785	2.62E-06	9.00E-06
ko00253_Tetracycline_biosynthesis	Valerat...mol.g.	-0.375940858	2.70E-06	9.19E-06
ko00240_Pyrimidine_metabolism	Beta.Defensin.2..ng.g.	0.374073941	2.82E-06	9.53E-06
ko00640_Propanoate_metabolism	Chromogranin.A..nmol.g.	-0.373024819	3.02E-06	1.01E-05
ko03050_Proteasome	Chromogranin.A..nmol.g.	-0.371716856	3.29E-06	1.09E-05
ko03013_RNA_transport	Propionat...mol.g.	-0.372957683	3.28E-06	1.09E-05
ko00240_Pyrimidine_metabolism	Chromogranin.A..nmol.g.	-0.371394144	3.36E-06	1.10E-05
ko00983_Drug_metabolism_<>_other_enzymes	Caproat...mol.g.	0.371855467	3.53E-06	1.15E-05
ko03008_Ribosome_biogenesis_in_eukaryotes	Chromogranin.A..nmol.g.	-0.370198439	3.64E-06	1.18E-05
ko00052_Galactose_metabolism	Caproat...mol.g.	-0.371074487	3.71E-06	1.19E-05
ko00130_Ubiquinone_and_other_terpenoid<>quinone_biosynthesis	Caproat...mol.g.	0.370942177	3.74E-06	1.20E-05
ko00642_Ethylbenzene_degradation	Butyrat...mol.g.	-0.370582424	3.83E-06	1.22E-05
ko00600_Sphingolipid_metabolism	Chromogranin.A..nmol.g.	0.368973119	3.94E-06	1.24E-05
ko00330_Arginine_and_proline_metabolism	Caproat...mol.g.	-0.36857572	4.36E-06	1.37E-05
ko05143_African_trypanosomiasis	Chromogranin.A..nmol.g.	-0.366903652	4.50E-06	1.40E-05
ko00513_Various_types_of_N<>glycan_biosynthesis	Chromogranin.A..nmol.g.	-0.365638261	4.88E-06	1.50E-05
ko03015_mRNA_surveillance_pathway	Chromogranin.A..nmol.g.	-0.365638261	4.88E-06	1.50E-05
ko00280_Valine_leucine_and_isoleucine_degradation	Chromogranin.A..nmol.g.	-0.36425323	5.34E-06	1.62E-05
ko00440_Phosphonate_and_phosphinate_metabolism	Caproat...mol.g.	-0.365449426	5.33E-06	1.62E-05
ko00300_Lysine_biosynthesis	Valerat...mol.g.	-0.365239469	5.40E-06	1.63E-05
ko00633_Nitrotoluene_degradation	Acetat...mol.g.	-0.364659314	5.60E-06	1.67E-05
ko05010_Alzheimer's_disease	Caproat...mol.g.	0.364719831	5.58E-06	1.67E-05
ko04112_Cell_cycle_<>_Caulobacter	Valerat...mol.g.	0.364009706	5.84E-06	1.72E-05
ko00620_Pyruvate_metabolism	Valerat...mol.g.	-0.362470142	6.43E-06	1.87E-05
ko03030_DNA_replication	Valerat...mol.g.	0.362540036	6.41E-06	1.87E-05
ko00363_Bisphenol_degradation	Caproat...mol.g.	-0.362419529	6.45E-06	1.87E-05
ko03410_Base_excision_repair	Chromogranin.A..nmol.g.	-0.360286673	6.86E-06	1.98E-05
ko00970_Aminoacyl<>tRNA_biosynthesis	Chromogranin.A..nmol.g.	-0.360092325	6.95E-06	1.98E-05
ko03020_RNA_polymerase	Propionat...mol.g.	-0.361295258	6.93E-06	1.98E-05
ko00410_beta<>Alanine_metabolism	Valerat...mol.g.	0.360037063	7.50E-06	2.12E-05
ko00190_Oxidative_phosphorylation	Valerat...mol.g.	0.359717816	7.65E-06	2.15E-05
ko00626_Naphthalene_degradation	Butyrat...mol.g.	-0.359422431	7.79E-06	2.18E-05
ko00624_Polycyclic_aromatic_hydrocarbon_degradation	Chromogranin.A..nmol.g.	0.357910402	7.97E-06	2.21E-05
ko00072_Synthesis_and_degradation_of_ketone_bodies	Propionat...mol.g.	-0.357961248	8.53E-06	2.36E-05
ko02020_Two<>component_system	Propionat...mol.g.	-0.357623125	8.71E-06	2.39E-05
ko03018_RNA_degradation	Beta.Defensin.2..ng.g.	0.355303331	9.37E-06	2.54E-05
ko00510_N<>Glycan_biosynthesis	Caproat...mol.g.	0.356448572	9.37E-06	2.54E-05
ko00040_Pentose_and_glucuronate_interconversions	Chromogranin.A..nmol.g.	0.354617254	9.77E-06	2.63E-05
ko04142_Lysosome	Propionat...mol.g.	0.35563783	9.85E-06	2.63E-05
ko02020_Two<>component_system	Caproat...mol.g.	-0.355722757	9.80E-06	2.63E-05
ko00071_Fatty_acid_metabolism	Chromogranin.A..nmol.g.	-0.354389589	9.91E-06	2.63E-05
ko00440_Phosphonate_and_phosphinate_metabolism	Valerat...mol.g.	-0.35467409	1.05E-05	2.76E-05
ko00240_Pyrimidine_metabolism	Valerat...mol.g.	0.354147049	1.08E-05	2.83E-05
ko02010_ABC_transporters	Valerat...mol.g.	-0.353508554	1.12E-05	2.93E-05
ko00531_Glycosaminoglycan_degradation	Propionat...mol.g.	0.352983845	1.16E-05	3.01E-05
ko02040_Flagellar_assembly	Propionat...mol.g.	-0.352672167	1.18E-05	3.05E-05
ko00660_C5<>Branched_dibasic_acid_metabolism	Beta.Defensin.2..ng.g.	-0.35087768	1.23E-05	3.14E-05
ko04626_Plant<>pathogen_interaction	Valerat...mol.g.	-0.352030716	1.23E-05	3.14E-05
ko03022_Basal_transcription_factors	Chromogranin.A..nmol.g.	-0.350469064	1.26E-05	3.20E-05
ko04141_Protein_processing_in_endoplasmic_reticulum	Propionat...mol.g.	0.351036329	1.30E-05	3.29E-05
ko00720_Carbon_fixation_pathways_in_prokaryotes	Valerat...mol.g.	0.350914908	1.31E-05	3.30E-05
ko03010_Ribosome	Beta.Defensin.2..ng.g.	0.349617175	1.33E-05	3.32E-05
ko04122_Sulfur_relay_system	Propionat...mol.g.	-0.349959624	1.39E-05	3.46E-05
ko00471_D<>Glutamine_and_D<>glutamate_metabolism	Beta.Defensin.2..ng.g.	0.348027198	1.46E-05	3.61E-05
ko00591_Linoleic_acid_metabolism	Valerat...mol.g.	-0.348908751	1.48E-05	3.65E-05
ko00908_Zeatin_biosynthesis	Beta.Defensin.2..ng.g.	0.347388616	1.52E-05	3.72E-05
ko04626_Plant<>pathogen_interaction	Propionat...mol.g.	-0.34802566	1.56E-05	3.81E-05
ko04112_Cell_cycle_<>_Caulobacter	Beta.Defensin.2..ng.g.	0.346659337	1.59E-05	3.84E-05
ko00010_Glycolysis_/_Gluconeogenesis	Acetat...mol.g.	-0.347367894	1.63E-05	3.92E-05
ko03010_Ribosome	Valerat...mol.g.	0.346902595	1.67E-05	4.01E-05
ko00660_C5<>Branched_dibasic_acid_metabolism	Butyrat...mol.g.	-0.346273359	1.74E-05	4.14E-05
ko04910_Insulin_signaling_pathway	Beta.Defensin.2..ng.g.	-0.343966187	1.87E-05	4.42E-05
ko03018_RNA_degradation	Caproat...mol.g.	0.344569032	1.92E-05	4.54E-05
ko00720_Carbon_fixation_pathways_in_prokaryotes	Chromogranin.A..nmol.g.	-0.34320808	1.95E-05	4.58E-05

ko00290_Valine_leucine_and_isoleucine_biosynthesis	Butyrat...mol.g.	-0.343934823	2.00E-05	4.67E-05
ko04910_Insulin_signaling_pathway	Caproat...mol.g.	-0.343824316	2.01E-05	4.67E-05
ko00510_N<>Glycan_biosynthesis	Beta.Defensin.2..ng.g.	0.342478013	2.04E-05	4.70E-05
ko02040_Flagellar_assembly	Valerat...mol.g.	-0.34348344	2.05E-05	4.70E-05
ko02030_Bacterial_chemotaxis	Valerat...mol.g.	-0.343460771	2.05E-05	4.70E-05
ko00030_Pentose_phosphate_pathway	Beta.Defensin.2..ng.g.	-0.342211474	2.07E-05	4.72E-05
ko05143_African_trypanosomiasis	Acetat...mol.g.	-0.342974664	2.11E-05	4.79E-05
ko02030_Bacterial_chemotaxis	Propionat...mol.g.	-0.342641802	2.16E-05	4.85E-05
ko03410_Base_excision_repair	Caproat...mol.g.	0.342631637	2.16E-05	4.85E-05
ko00030_Pentose_phosphate_pathway	Propionat...mol.g.	-0.342383015	2.19E-05	4.89E-05
ko00330_Arginine_and_proline_metabolism	Beta.Defensin.2..ng.g.	-0.341117556	2.21E-05	4.92E-05
ko04112_Cell_cycle_<>_Caulobacter	Chromogranin.A..nmol.g.	-0.340883304	2.24E-05	4.96E-05
ko00190_Oxidative_phosphorylation	Chromogranin.A..nmol.g.	-0.340353936	2.31E-05	5.09E-05
ko00281_Geraniol_degradation	Propionat...mol.g.	0.341151414	2.35E-05	5.16E-05
ko03020_RNA_polymerase	Butyrat...mol.g.	-0.340948373	2.38E-05	5.20E-05
ko00072_Synthesis_and_degradation_of_ketone_bodies	Valerat...mol.g.	-0.339593914	2.58E-05	5.60E-05
ko00500_Starch_and_sucrose_metabolism	Valerat...mol.g.	-0.339099307	2.65E-05	5.71E-05
ko00590_Arachidonic_acid_metabolism	Caproat...mol.g.	0.339149996	2.64E-05	5.71E-05
ko00430_Taurine_and_hypotaurine_metabolism	Valerat...mol.g.	0.338265922	2.78E-05	5.97E-05
ko00511_Other_glycan_degradation	Propionat...mol.g.	0.337288814	2.95E-05	6.29E-05
ko04122_Sulfur_relay_system	Beta.Defensin.2..ng.g.	-0.335899693	3.00E-05	6.37E-05
ko00020_Citrate_cycle_[TCA_cycle]	Caproat...mol.g.	0.336843396	3.02E-05	6.39E-05
ko00430_Taurine_and_hypotaurine_metabolism	Chromogranin.A..nmol.g.	-0.335506333	3.07E-05	6.45E-05
ko00450_Selenocompound_metabolism	Butyrat...mol.g.	0.33652631	3.08E-05	6.45E-05
ko00362_Benzoate_degradation	Acetat...mol.g.	-0.336406335	3.10E-05	6.47E-05
ko00740_Riboflavin_metabolism	Butyrat...mol.g.	0.336055959	3.16E-05	6.54E-05
ko03008_Ribosome_biogenesis_in_eukaryotes	Caproat...mol.g.	0.336059614	3.16E-05	6.54E-05
ko00983_Drug_metabolism_<>_other_enzymes	Valerat...mol.g.	0.335551694	3.26E-05	6.71E-05
ko00623_Toluene_degradation	Valerat...mol.g.	0.334327282	3.49E-05	7.16E-05
ko04210_Apoptosis	Caproat...mol.g.	0.334081587	3.54E-05	7.23E-05
ko00680_Methane_metabolism	Valerat...mol.g.	-0.3339967	3.56E-05	7.24E-05
ko00051_Fructose_and_mannose_metabolism	Valerat...mol.g.	-0.333810945	3.60E-05	7.28E-05
ko00500_Starch_and_sucrose_metabolism	Caproat...mol.g.	-0.333639603	3.63E-05	7.32E-05
ko00626_Naphthalene_degradation	Acetat...mol.g.	-0.332171316	3.95E-05	7.91E-05
ko00071_Fatty_acid_metabolism	Butyrat...mol.g.	-0.332149583	3.96E-05	7.91E-05
ko00460_Cyanoamino_acid_metabolism	Butyrat...mol.g.	0.331932352	4.01E-05	7.97E-05
ko00540_Lipopolysaccharide_biosynthesis	Caproat...mol.g.	0.331472241	4.11E-05	8.15E-05
ko03440_Homologous_recombination	Valerat...mol.g.	0.330715757	4.29E-05	8.47E-05
ko03420_Nucleotide_excision_repair	Beta.Defensin.2..ng.g.	0.328954882	4.46E-05	8.77E-05
ko00550_Peptidoglycan_biosynthesis	Caproat...mol.g.	0.329940593	4.48E-05	8.77E-05
ko00540_Lipopolysaccharide_biosynthesis	Acetat...mol.g.	0.329802577	4.52E-05	8.80E-05
ko00471_D<>Glutamine_and_D<>glutamate_metabolism	Valerat...mol.g.	0.328473159	4.87E-05	9.45E-05
ko00052_Galactose_metabolism	Beta.Defensin.2..ng.g.	-0.326722621	5.06E-05	9.78E-05
ko03430_Mismatch_repair	Chromogranin.A..nmol.g.	-0.325781512	5.33E-05	0.00010266
ko00564_Glycerophospholipid_metabolism	Propionat...mol.g.	-0.326366543	5.48E-05	0.000105002
ko00720_Carbon_fixation_pathways_in_prokaryotes	Beta.Defensin.2..ng.g.	0.324853149	5.62E-05	0.000107247
ko02060_Phosphotransferase_system_[PTS]	Propionat...mol.g.	-0.324645702	6.03E-05	0.0001146
ko00290_Valine_leucine_and_isoleucine_biosynthesis	Beta.Defensin.2..ng.g.	-0.323505649	6.06E-05	0.000114667
ko00500_Starch_and_sucrose_metabolism	Beta.Defensin.2..ng.g.	-0.321869696	6.63E-05	0.000125034
ko00770_Pantothenate_and_CoA_biosynthesis	Valerat...mol.g.	-0.322224227	6.89E-05	0.000129401
ko00540_Lipopolysaccharide_biosynthesis	Beta.Defensin.2..ng.g.	0.320471921	7.16E-05	0.000133964
ko00621_Dioxin_degradation	Valerat...mol.g.	-0.320575099	7.54E-05	0.000140506
ko00450_Selenocompound_metabolism	Propionat...mol.g.	0.319639965	7.93E-05	0.00014727
ko00010_Glycolysis_/_Gluconeogenesis	Butyrat...mol.g.	-0.318834283	8.29E-05	0.000152655
ko00030_Pentose_phosphate_pathway	Caproat...mol.g.	-0.318883898	8.27E-05	0.000152655
ko04146_Peroxisome	Propionat...mol.g.	0.317902124	8.72E-05	0.000159935
ko01040_Biosynthesis_of_unsaturated_fatty_acids	Butyrat...mol.g.	-0.317591346	8.87E-05	0.000162012
ko03450_Non<>homologous_end<>joining	Butyrat...mol.g.	-0.316724312	9.29E-05	0.000169112
ko00051_Fructose_and_mannose_metabolism	Chromogranin.A..nmol.g.	0.315067656	9.61E-05	0.000173547
ko00450_Selenocompound_metabolism	Acetat...mol.g.	0.316134157	9.59E-05	0.000173547
ko00531_Glycosaminoglycan_degradation	Valerat...mol.g.	0.315317911	0.000100201	0.000180291
ko00622_Xylene_degradation	Valerat...mol.g.	-0.314756867	0.000103257	0.00018508
ko04113_Meiosis_<>_yeast	Chromogranin.A..nmol.g.	-0.311855792	0.000114133	0.000203688
ko00670_One_carbon_pool_by_folate	Chromogranin.A..nmol.g.	-0.311723648	0.00011494	0.000203688
ko00471_D<>Glutamine_and_D<>glutamate_metabolism	Propionat...mol.g.	0.312805716	0.000114576	0.000203688
ko00900_Terpenoid_backbone_biosynthesis	Valerat...mol.g.	0.312291675	0.000117745	0.000207875
ko00623_Toluene_degradation	Propionat...mol.g.	0.312188027	0.000118394	0.000208237

ko04974_Protein_digestion_and_absorption	Acetat...mol.g.	0.31134597	0.00012379	0.000216916
ko00720_Carbon_fixation_pathways_in_prokaryotes	Caproat...mol.g.	0.311087064	0.000125494	0.000219086
ko00360_Phenylalanine_metabolism	Butyrat...mol.g.	-0.311012087	0.000125992	0.00021914
ko03018_RNA_degradation	Chromogranin.A..nmol.g.	-0.309869009	0.000126834	0.00021979
ko04146_Peroxisome	Chromogranin.A..nmol.g.	-0.308062495	0.000139515	0.000240876
ko00281_Geraniol_degradation	Beta.Defensin.2..ng.g.	0.30789093	0.000140779	0.000242168
ko03018_RNA_degradation	Propionat...mol.g.	0.308672677	0.00014248	0.0002442
ko03420_Nucleotide_excision_repair	Chromogranin.A..nmol.g.	-0.307362841	0.000144736	0.000247165
ko00364_Fluorobenzoate_degradation	Chromogranin.A..nmol.g.	-0.306435254	0.00015194	0.000258526
ko00785_Lipoic_acid_metabolism	Chromogranin.A..nmol.g.	-0.30408113	0.000171748	0.000289087
ko00590_Arachidonic_acid_metabolism	Beta.Defensin.2..ng.g.	0.304124222	0.000171365	0.000289087
ko02060_Phosphotransferase_system_[PTS]	Valerat...mol.g.	-0.305156786	0.000171077	0.000289087
ko00908_Zeatin_biosynthesis	Caproat...mol.g.	0.304679484	0.000175347	0.000294091
ko00903_Limonene_and_pinene_degradation	Acetat...mol.g.	-0.303138202	0.000189823	0.000317205
ko03450_Non<>homologous_end<>joining	Propionat...mol.g.	-0.303002026	0.000191154	0.000317205
ko02010_ABC_transporters	Propionat...mol.g.	-0.303011471	0.000191062	0.000317205
ko00591_Linoleic_acid_metabolism	Propionat...mol.g.	-0.302747017	0.00019367	0.000320248
ko02020_Two<>component_system	Beta.Defensin.2..ng.g.	-0.301481048	0.000196405	0.000323631
ko00590_Arachidonic_acid_metabolism	Propionat...mol.g.	0.301715646	0.000204163	0.000335238
ko04141_Protein_processing_in_endoplasmic_reticulum	Beta.Defensin.2..ng.g.	0.300429701	0.00020728	0.000339169
ko00601_Glycosphingolipid_biosynthesis_<>_lacto_and_neolacto_series	Caproat...mol.g.	-0.299835611	0.000224662	0.000366335
ko00020_Citrate_cycle_[TCA_cycle]	Propionat...mol.g.	0.299598407	0.00022738	0.000369484
ko03420_Nucleotide_excision_repair	Valerat...mol.g.	0.299467009	0.000228899	0.00037067
ko04113_Meiosis_<>_yeast	Calprotectin...g.g.	-0.297185975	0.000244459	0.000394507
ko00312_beta<>Lactam_resistance	Caproat...mol.g.	-0.29768785	0.000250412	0.00040273
ko00750_Vitamin_B6_metabolism	Valerat...mol.g.	0.29737046	0.000254442	0.000407814
ko00410_beta<>Alanine_metabolism	Caproat...mol.g.	0.297298481	0.000255364	0.0004079
ko00920_Sulfur_metabolism	Beta.Defensin.2..ng.g.	-0.296192848	0.000257025	0.000409162
ko00260_Glycine_serine_and_threonine_metabolism	Propionat...mol.g.	0.296645684	0.00026387	0.00041864
ko00561_Glycerolipid_metabolism	Acetat...mol.g.	-0.296519332	0.000265547	0.000419881
ko00480_Glutathione_metabolism	Acetat...mol.g.	0.296050873	0.000271849	0.000428404
ko00760_Nicotinate_and_nicotinamide_metabolism	Propionat...mol.g.	0.295828044	0.000274896	0.000431756
ko00471_D<>Glutamine_and_D<>glutamate_metabolism	Butyrat...mol.g.	0.295403586	0.000280786	0.000439538
ko00540_Lipopolysaccharide_biosynthesis	Butyrat...mol.g.	0.294914345	0.000287721	0.000448897
ko03070_Bacterial_secretion_system	Caproat...mol.g.	0.294385773	0.000295391	0.000459338
ko00071_Fatty_acid_metabolism	Acetat...mol.g.	-0.293387458	0.000310398	0.00048108
ko03020_RNA_polymerase	Acetat...mol.g.	-0.293073893	0.000315255	0.000487001
ko04142_Lysosome	Valerat...mol.g.	0.292929055	0.000317522	0.000488896
ko00130_Ubiquinone_and_other_terpenoid<>quinone_biosynthesis	Beta.Defensin.2..ng.g.	0.29161912	0.000323016	0.000495729
ko00473_D<>Alanine_metabolism	Caproat...mol.g.	0.292510752	0.000324156	0.000495858
ko00561_Glycerolipid_metabolism	Butyrat...mol.g.	-0.292360467	0.00032657	0.000497929
ko01053_Biosynthesis_of_siderophore_group_nonribosomal_peptides	Propionat...mol.g.	0.292189632	0.000329335	0.000500519
ko05143_African_trypanosomiasis	Butyrat...mol.g.	-0.291888859	0.000334255	0.000506358
ko00230_Purine_metabolism	Chromogranin.A..nmol.g.	-0.290508212	0.000341257	0.000513783
ko02060_Phosphotransferase_system_[PTS]	Acetat...mol.g.	-0.291462621	0.000341344	0.000513783
ko00440_Phosphonate_and_phosphinate_metabolism	Beta.Defensin.2..ng.g.	-0.289536788	0.000357984	0.000537108
ko03008_Ribosome_biogenesis_in_eukaryotes	Beta.Defensin.2..ng.g.	0.289057388	0.000366516	0.000547448
ko00623_Toluene_degradation	Acetat...mol.g.	0.289974131	0.000367208	0.000547448
ko05142_Chagas_disease_[American_trypanosomiasis]	Calprotectin...g.g.	-0.288817688	0.000370852	0.000551132
ko00750_Vitamin_B6_metabolism	Caproat...mol.g.	0.289377171	0.000378081	0.000560101
ko00480_Glutathione_metabolism	Beta.Defensin.2..ng.g.	0.287802436	0.000389749	0.000575572
ko00190_Oxidative_phosphorylation	Beta.Defensin.2..ng.g.	0.287454455	0.000396428	0.000582047
ko00052_Galactose_metabolism	Valerat...mol.g.	-0.288395368	0.000396612	0.000582047
ko00260_Glycine_serine_and_threonine_metabolism	Acetat...mol.g.	0.2875355	0.000413529	0.000604982
ko03450_Non<>homologous_end<>joining	Acetat...mol.g.	-0.287127488	0.000421787	0.000615147
ko00511_Other_glycan_degradation	Acetat...mol.g.	0.286602631	0.000432634	0.000629013
ko00190_Oxidative_phosphorylation	Propionat...mol.g.	0.286258306	0.000439889	0.000637588
ko00350_Tyrosine_metabolism	Butyrat...mol.g.	-0.285631981	0.000453376	0.000655114
ko04622_RIG<>I<>like_receptor_signaling_pathway	Beta.Defensin.2..ng.g.	-0.284299053	0.000462009	0.000665541
ko00140_Steroid_hormone_biosynthesis	Propionat...mol.g.	0.284828365	0.000471239	0.000676761
ko00440_Phosphonate_and_phosphinate_metabolism	Propionat...mol.g.	-0.284214453	0.000485321	0.00069486
ko00983_Drug_metabolism_<>_other_enzymes	Propionat...mol.g.	0.283069068	0.000512637	0.00073039
ko04210_Apoptosis	Valerat...mol.g.	0.283044103	0.000513248	0.00073039
ko00010_Glycolysis_/_Gluconeogenesis	Valerat...mol.g.	-0.282820822	0.000518741	0.000735978
ko00260_Glycine_serine_and_threonine_metabolism	Butyrat...mol.g.	0.282624752	0.00052361	0.000740648
ko00633_Nitrotoluene_degradation	Valerat...mol.g.	-0.281897084	0.00054205	0.000764428
ko00260_Glycine_serine_and_threonine_metabolism	Caproat...mol.g.	0.281300322	0.000557617	0.000784027

ko04974_Protein_digestion_and_absorption	Butyrat...mol.g.	0.280926839	0.000567568	0.000795637
ko00623_Toluene_degradation	Butyrat...mol.g.	0.28053367	0.00057822	0.000807723
ko05120_Epithelial_cell_signaling_in_Helicobacter_pylori_infection	Caproat...mol.g.	0.280482156	0.00057963	0.000807723
ko00523_Polyketide_sugar_unit_biosynthesis	Propionat...mol.g.	0.278405909	0.000639134	0.000888008
ko04910_Insulin_signaling_pathway	Propionat...mol.g.	-0.278271793	0.000643165	0.000890973
ko00642_Ethylbenzene_degradation	Propionat...mol.g.	-0.278081008	0.000648939	0.000896327
ko00930_Caprolactam_degradation	Chromogranin.A..nmol.g.	-0.276924188	0.000656214	0.000903717
ko00740_Riboflavin_metabolism	Propionat...mol.g.	0.277300868	0.00067305	0.000924193
ko00770_Pantothenate_and_CoA_biosynthesis	Beta.Defensin.2..ng.g.	-0.276252431	0.000677204	0.000927186
ko00020_Citrate_cycle_[TCA_cycle]	Beta.Defensin.2..ng.g.	0.275825114	0.000690876	0.000943155
ko00900_Terpenoid_backbone_biosynthesis	Chromogranin.A..nmol.g.	-0.275524879	0.000700633	0.000953703
ko00380_Tryptophan_metabolism	Calprotectin...g.g.	-0.275329869	0.000707039	0.000959641
ko00051_Fructose_and_mannose_metabolism	Butyrat...mol.g.	-0.276178927	0.000709171	0.000959761
ko00860_Porphyrin_and_chlorophyll_metabolism	Valerat...mol.g.	-0.275701045	0.000725091	0.000978487
ko05012_Parkinson's_disease	Acetat...mol.g.	-0.275296544	0.000738823	0.000994161
ko02010_ABC_transporters	Caproat...mol.g.	-0.275106328	0.000745362	0.001000095
ko00310_Lysine_degradation	Butyrat...mol.g.	-0.275012231	0.000748617	0.0010016
ko00260_Glycine_serine_and_threonine_metabolism	Valerat...mol.g.	0.274748971	0.000757792	0.001010995
ko00061_Fatty_acid_biosynthesis	Valerat...mol.g.	-0.274634259	0.000761822	0.001013493
ko00633_Nitrotoluene_degradation	Butyrat...mol.g.	-0.274492766	0.000766821	0.00101726
ko00310_Lysine_degradation	Calprotectin...g.g.	-0.27347025	0.000770882	0.001019768
ko00300_Lysine_biosynthesis	Acetat...mol.g.	-0.27399364	0.000784694	0.001035123
ko00310_Lysine_degradation	Propionat...mol.g.	-0.273103605	0.000817517	0.001072397
ko00920_Sulfur_metabolism	Valerat...mol.g.	-0.273131956	0.000816452	0.001072397
ko03020_RNA_polymerase	Valerat...mol.g.	-0.272606804	0.000836382	0.001094087
ko00903_Limonene_and_pinene_degradation	Butyrat...mol.g.	-0.272413018	0.000843848	0.001100788
ko01040_Biosynthesis_of_unsaturated_fatty_acids	Acetat...mol.g.	-0.272276587	0.000849142	0.001104624
ko00020_Citrate_cycle_[TCA_cycle]	Chromogranin.A..nmol.g.	-0.271159696	0.000857576	0.001112514
ko00312_beta<>Lactam_resistance	Beta.Defensin.2..ng.g.	-0.270240505	0.000894486	0.001147715
ko00903_Limonene_and_pinene_degradation	Calprotectin...g.g.	-0.270307	0.000891768	0.001147715
ko05145_Toxoplasmosis	Acetat...mol.g.	-0.271214976	0.000891382	0.001147715
ko05012_Parkinson's_disease	Butyrat...mol.g.	-0.271154327	0.000893853	0.001147715
ko04115_p53_signaling_pathway	Acetat...mol.g.	-0.270914115	0.000903699	0.001156377
ko00051_Fructose_and_mannose_metabolism	Propionat...mol.g.	-0.270469709	0.000922178	0.001176816
ko00190_Oxidative_phosphorylation	Caproat...mol.g.	0.270360183	0.000926785	0.00117949
ko03430_Mismatch_repair	Valerat...mol.g.	0.270226619	0.000932432	0.00118028
ko00860_Porphyrin_and_chlorophyll_metabolism	Caproat...mol.g.	-0.270246774	0.000931578	0.00118028
ko00360_Phenylalanine_metabolism	Acetat...mol.g.	-0.270062741	0.000939404	0.001185908
ko00650_Butanoate_metabolism	Butyrat...mol.g.	-0.269836472	0.000949108	0.001194946
ko00941_Flavonoid_biosynthesis	Butyrat...mol.g.	-0.269475681	0.000964772	0.00121142
ko05143_African_trypanosomiasis	Caproat...mol.g.	0.268756679	0.000996696	0.001248169
ko00750_Vitamin_B6_metabolism	Propionat...mol.g.	0.268617584	0.001002983	0.001252701
ko00350_Tyrosine_metabolism	Propionat...mol.g.	-0.268103534	0.001026533	0.001268618
ko03022_Basal_transcription_factors	Butyrat...mol.g.	-0.268271085	0.001018802	0.001268618
ko00363_Bisphenol_degradation	Valerat...mol.g.	-0.268129788	0.001025318	0.001268618
ko00660_C5<>Branched_dibasic_acid_metabolism	Caproat...mol.g.	-0.268216762	0.001021302	0.001268618
ko00983_Drug_metabolism_<>_other_enzymes	Beta.Defensin.2..ng.g.	0.266927529	0.00103994	0.001281814
ko03013_RNA_transport	Beta.Defensin.2..ng.g.	-0.266818075	0.001045096	0.001284796
ko00380_Tryptophan_metabolism	Valerat...mol.g.	0.267432734	0.001058025	0.001297295
ko05145_Toxoplasmosis	Butyrat...mol.g.	-0.267086117	0.001074642	0.001314239
ko00340_Histidine_metabolism	Beta.Defensin.2..ng.g.	-0.266049926	0.001081943	0.00131973
ko04115_p53_signaling_pathway	Butyrat...mol.g.	-0.266735744	0.001091682	0.00132816
ko00564_Glycerophospholipid_metabolism	Beta.Defensin.2..ng.g.	-0.265574229	0.001105353	0.001341318
ko01040_Biosynthesis_of_unsaturated_fatty_acids	Propionat...mol.g.	-0.266108795	0.001122791	0.001358966
ko00400_Phenylalanine_tyrosine_and_tryptophan_biosynthesis	Valerat...mol.g.	-0.265632483	0.001146965	0.001384657
ko03320_PPAR_signaling_pathway	Valerat...mol.g.	0.265543698	0.001151523	0.001386595
ko03070_Bacterial_secretion_system	Calprotectin...g.g.	-0.264251853	0.001172889	0.001405117
ko00130_Ubiquinone_and_other_terpenoid<>quinone_biosynthesis	Butyrat...mol.g.	0.265170734	0.001170853	0.001405117
ko00908_Zeatin_biosynthesis	Acetat...mol.g.	0.264764245	0.001192259	0.001424687
ko00360_Phenylalanine_metabolism	Propionat...mol.g.	-0.2644314	0.001210052	0.001442279
ko00770_Pantothenate_and_CoA_biosynthesis	Propionat...mol.g.	-0.264372842	0.001213207	0.001442379
ko00410_beta<>Alanine_metabolism	Propionat...mol.g.	0.263502033	0.001261026	0.001495445
ko00564_Glycerophospholipid_metabolism	Valerat...mol.g.	-0.263342971	0.001269944	0.001502228
ko00650_Butanoate_metabolism	Valerat...mol.g.	-0.262950051	0.001292222	0.00152474
ko00361_Chlorocyclohexane_and_chlorobenzene_degradation	Valerat...mol.g.	-0.262211438	0.001335069	0.001571349
ko00130_Ubiquinone_and_other_terpenoid<>quinone_biosynthesis	Acetat...mol.g.	0.26167015	0.001367289	0.001605247
ko03013_RNA_transport	Caproat...mol.g.	-0.261389571	0.001384268	0.001621129

ko00550_Peptidoglycan_biosynthesis	Beta.Defensin.2..ng.g.	0.258775884	0.001494624	0.001739351
ko03008_Ribosome_biogenesis_in_eukaryotes	Valerat...mol.g.	0.259693353	0.001491076	0.001739351
ko00940_Phenylpropanoid_biosynthesis	Caproat...mol.g.	-0.259612838	0.001496328	0.001739351
ko00785_Lipoic_acid_metabolism	Valerat...mol.g.	0.259011411	0.001536095	0.001781168
ko00624_Polycyclic_aromatic_hydrocarbon_degradation	Acetat...mol.g.	0.257869833	0.001614238	0.001862579
ko04146_Peroxisome	Caproat...mol.g.	0.257883358	0.001613291	0.001862579
ko00471_D<>Glutamine_and_D<>glutamate_metabolism	Acetat...mol.g.	0.257138567	0.001666179	0.00191311
ko00230_Purine_metabolism	Propionat...mol.g.	0.257194803	0.001662131	0.00191311
ko00860_Porphyrin_and_chlorophyll_metabolism	Beta.Defensin.2..ng.g.	-0.25563271	0.001713808	0.001962998
ko00072_Synthesis_and_degradation_of_ketone_bodies	Caproat...mol.g.	-0.256244605	0.001731741	0.001978713
ko00351_DDT_degradation	Calprotectin...g.g.	-0.255311764	0.00173776	0.001980771
ko00511_Other_glycan_degradation	Butyrat...mol.g.	0.256060507	0.001745531	0.001984811
ko00561_Glycerolipid_metabolism	Beta.Defensin.2..ng.g.	-0.254871964	0.001771077	0.002004246
ko00312_beta<>Lactam_resistance	Acetat...mol.g.	0.255721856	0.001771158	0.002004246
ko00908_Zeatin_biosynthesis	Butyrat...mol.g.	0.253400606	0.001956235	0.002208358
ko00364_Fluorobenzoate_degradation	Calprotectin...g.g.	-0.252315504	0.001976515	0.0022259
ko03410_Base_excision_repair	Beta.Defensin.2..ng.g.	0.252215833	0.001984946	0.002230048
ko00670_One_carbon_pool_by_folate	Propionat...mol.g.	0.252457298	0.002036342	0.002282329
ko00460_Cyanoamino_acid_metabolism	Acetat...mol.g.	0.252274528	0.002052203	0.002286864
ko00400_Phenylalanine_tyrosine_and_tryptophan_biosynthesis	Propionat...mol.g.	-0.252319404	0.002048298	0.002286864
ko00051_Fructose_and_mannose_metabolism	Caproat...mol.g.	-0.252242469	0.002054996	0.002286864
ko00565_Ether_lipid_metabolism	Beta.Defensin.2..ng.g.	-0.249835263	0.002196284	0.002428749
ko00650_Butanoate_metabolism	Acetat...mol.g.	-0.250651923	0.002198011	0.002428749
ko00600_Sphingolipid_metabolism	Caproat...mol.g.	-0.250751854	0.002188767	0.002428749
ko03070_Bacterial_secretion_system	Valerat...mol.g.	0.250499414	0.002212188	0.002436153
ko00340_Histidine_metabolism	Caproat...mol.g.	-0.250468333	0.002215087	0.002436153
ko00351_DDT_degradation	Acetat...mol.g.	0.250081283	0.002251482	0.002470395
ko02010_ABC_transporters	Beta.Defensin.2..ng.g.	-0.249130279	0.002262676	0.00247689
ko00562_Inositol_phosphate_metabolism	Chromogranin.A..nmol.g.	-0.249071523	0.00226829	0.002477262
ko00510_N<>Glycan_biosynthesis	Butyrat...mol.g.	0.249216553	0.00233476	0.002543938
ko00480_Glutathione_metabolism	Butyrat...mol.g.	0.249131549	0.002343095	0.002547111
ko00310_Lysine_degradation	Acetat...mol.g.	-0.249016095	0.002354459	0.002553553
ko03022_Basal_transcription_factors	Calprotectin...g.g.	-0.247869271	0.002385984	0.002576883
ko00363_Bisphenol_degradation	Propionat...mol.g.	-0.248688827	0.002386944	0.002576883
ko00625_Chloroalkane_and_chloroalkene_degradation	Chromogranin.A..nmol.g.	-0.247181486	0.002455776	0.002645112
ko00300_Lysine_biosynthesis	Beta.Defensin.2..ng.g.	-0.246783259	0.002497026	0.002677261
ko00600_Sphingolipid_metabolism	Acetat...mol.g.	0.247652275	0.002492531	0.002677261
ko00030_Pentose_phosphate_pathway	Chromogranin.A..nmol.g.	0.246572528	0.002519107	0.002691426
ko00312_beta<>Lactam_resistance	Butyrat...mol.g.	0.247372925	0.002521701	0.002691426
ko00510_N<>Glycan_biosynthesis	Acetat...mol.g.	0.247249929	0.002534641	0.002699104
ko05146_Amoebiasis	Acetat...mol.g.	-0.2470171	0.002559303	0.0027192
ko00643_Styrene_degradation	Caproat...mol.g.	-0.246858162	0.002576263	0.00273104
ko00970_Aminoacyl<>tRNA_biosynthesis	Beta.Defensin.2..ng.g.	0.245691191	0.002613385	0.002764153
ko00195_Photosynthesis	Butyrat...mol.g.	0.245837198	0.002687652	0.002836316
ko00521_Streptomycin_biosynthesis	Propionat...mol.g.	0.245689163	0.002704159	0.002847338
ko00250_Alanine_aspartate_and_glutamate_metabolism	Beta.Defensin.2..ng.g.	-0.244593797	0.002735231	0.002873612
ko00642_Ethylbenzene_degradation	Valerat...mol.g.	-0.245323455	0.002745333	0.002877787
ko00300_Lysine_biosynthesis	Caproat...mol.g.	-0.244792237	0.002806151	0.002934989
ko00626_Naphthalene_degradation	Propionat...mol.g.	-0.243458304	0.002964274	0.003093482
ko04974_Protein_digestion_and_absorption	Beta.Defensin.2..ng.g.	0.242140344	0.00302642	0.003151333
ko00643_Styrene_degradation	Propionat...mol.g.	-0.242672497	0.003061146	0.00318044
ko00710_Carbon_fixation_in_photosynthetic_organisms	Caproat...mol.g.	0.242235791	0.003116208	0.003230501
ko02030_Bacterial_chemotaxis	Caproat...mol.g.	-0.242170611	0.003124502	0.003231965
ko00430_Taurine_and_hypotaurine_metabolism	Caproat...mol.g.	0.242036411	0.003141643	0.003242553
ko00280_Valine_leucine_and_isoleucine_degradation	Valerat...mol.g.	0.24178718	0.0031737	0.003268457
ko03013_RNA_transport	Acetat...mol.g.	-0.241683203	0.003187161	0.003275137
ko00591_Linoleic_acid_metabolism	Butyrat...mol.g.	-0.240997675	0.003277208	0.003360316
ko00513_Various_types_of_N<>glycan_biosynthesis	Butyrat...mol.g.	-0.240346936	0.003364799	0.003435129
ko03015_mRNA_surveillance_pathway	Butyrat...mol.g.	-0.240346936	0.003364799	0.003435129
ko00480_Glutathione_metabolism	Caproat...mol.g.	0.239944024	0.003420085	0.003483996
ko00380_Tryptophan_metabolism	Caproat...mol.g.	0.239626481	0.003464231	0.003521329
ko00361_Chlorocyclohexane_and_chlorobenzene_degradation	Caproat...mol.g.	-0.239454478	0.003488358	0.003538194
ko00860_Porphyrin_and_chlorophyll_metabolism	Chromogranin.A..nmol.g.	0.23837473	0.003528007	0.003570698
ko00400_Phenylalanine_tyrosine_and_tryptophan_biosynthesis	Chromogranin.A..nmol.g.	0.238063772	0.003572599	0.003608054
ko05012_Parkinson's_disease	Propionat...mol.g.	-0.238686213	0.003597978	0.003625887
ko05145_Toxoplasmosis	Propionat...mol.g.	-0.238083506	0.003686136	0.003706774
ko00340_Histidine_metabolism	Valerat...mol.g.	-0.23792599	0.003709494	0.003722292

ko04115_p53_signaling_pathway	Propionat...mol.g.	-0.237784547	0.003730582	0.003735471
ko04974_Protein_digestion_and_absorption	Caproat...mol.g.	0.237715772	0.003740874	0.003737807
ko00195_Photosynthesis	Acetat...mol.g.	0.237627634	0.003754102	0.00374306
ko00030_Pentose_phosphate_pathway	Butyrat...mol.g.	-0.237168747	0.003823654	0.00380433
ko00051_Fructose_and_mannose_metabolism	Acetat...mol.g.	-0.236196035	0.003974928	0.003946479
ko05100_Bacterial_invasion_of_epithelial_cells	Valerat...mol.g.	0.236080661	0.003993223	0.003956279
ko00624_Polycyclic_aromatic_hydrocarbon_degradation	Calprotectin...g.g.	0.235208808	0.004006342	0.003957324
ko04622_RIG<>I<>like_receptor_signaling_pathway	Caproat...mol.g.	-0.235968182	0.004011132	0.003957324
ko04146_Peroxisome	Beta.Defensin.2..ng.g.	0.234894528	0.00405687	0.003994058
ko00906_Carotenoid_biosynthesis	Chromogranin.A..nmol.g.	0.234692773	0.004089608	0.004017866
ko02030_Bacterial_chemotaxis	Beta.Defensin.2..ng.g.	-0.23443734	0.004131396	0.004050447
ko00312_beta<>Lactam_resistance	Chromogranin.A..nmol.g.	0.234321178	0.004150526	0.004060725
ko04112_Cell_cycle_<>_Caulobacter	Propionat...mol.g.	0.234937216	0.004178673	0.004079763
ko00650_Butanoate_metabolism	Chromogranin.A..nmol.g.	-0.233643735	0.004263685	0.004154127
ko00350_Tyrosine_metabolism	Valerat...mol.g.	-0.234276372	0.004289343	0.004170473
ko00250_Alanine_aspartate_and_glutamate_metabolism	Acetat...mol.g.	0.233972742	0.004341067	0.004212043
ko00600_Sphingolipid_metabolism	Propionat...mol.g.	0.232715802	0.004561197	0.004416505
ko00250_Alanine_aspartate_and_glutamate_metabolism	Caproat...mol.g.	-0.232053847	0.004681102	0.00452328
ko00624_Polycyclic_aromatic_hydrocarbon_degradation	Butyrat...mol.g.	0.230553816	0.004963326	0.004786142
ko00642_Ethylbenzene_degradation	Acetat...mol.g.	-0.230313631	0.005009904	0.004821157
ko02060_Phosphotransferase_system_[PTS]	Butyrat...mol.g.	-0.22994913	0.005081337	0.004879898
ko04146_Peroxisome	Butyrat...mol.g.	0.229828237	0.005105229	0.004892838
ko00511_Other_glycan_degradation	Chromogranin.A..nmol.g.	0.228715828	0.005173673	0.004928309
ko00790_Folate_biosynthesis	Acetat...mol.g.	0.22954483	0.005161633	0.004928309
ko00300_Lysine_biosynthesis	Butyrat...mol.g.	-0.229484445	0.005173723	0.004928309
ko03018_RNA_degradation	Acetat...mol.g.	0.228071451	0.005463946	0.005183736
ko00460_Cyanoamino_acid_metabolism	Caproat...mol.g.	-0.228121189	0.005453488	0.005183736
ko00195_Photosynthesis	Chromogranin.A..nmol.g.	0.226412126	0.005655818	0.00535495
ko04113_Meiosis_<>_yeast	Beta.Defensin.2..ng.g.	0.226303543	0.005679502	0.005366554
ko00351_DDT_degradation	Butyrat...mol.g.	0.226941134	0.005706486	0.005381225
ko00960_Tropane_piperidine_and_pyridine_alkaloid_biosynthesis	Beta.Defensin.2..ng.g.	0.225241759	0.005915809	0.005567437
ko05146_Amoebiasis	Butyrat...mol.g.	-0.22565963	0.005993053	0.005628852
ko04142_Lysosome	Acetat...mol.g.	0.225374035	0.006058639	0.005679094
ko00340_Histidine_metabolism	Chromogranin.A..nmol.g.	0.224327971	0.006126158	0.005730944
ko00361_Chlorocyclohexane_and_chlorobenzene_degradation	Beta.Defensin.2..ng.g.	-0.22382022	0.006245899	0.005830717
ko00600_Sphingolipid_metabolism	Butyrat...mol.g.	0.224524029	0.006257644	0.005830717
ko00790_Folate_biosynthesis	Chromogranin.A..nmol.g.	-0.223539472	0.006312998	0.005870647
ko00780_Biotin_metabolism	Valerat...mol.g.	0.224149249	0.006347222	0.005890808
ko00565_Ether_lipid_metabolism	Calprotectin...g.g.	0.223156546	0.006405552	0.005933218
ko04626_Plant<>pathogen_interaction	Beta.Defensin.2..ng.g.	-0.222994897	0.006444985	0.005957991
ko03020_RNA_polymerase	Caproat...mol.g.	-0.223499792	0.00650516	0.006001805
ko00623_Toluene_degradation	Beta.Defensin.2..ng.g.	0.222546758	0.006555435	0.006036331
ko00250_Alanine_aspartate_and_glutamate_metabolism	Butyrat...mol.g.	0.223024403	0.006622975	0.006086588
ko00053_Ascorbate_and_aldarate_metabolism	Butyrat...mol.g.	-0.222417835	0.006776055	0.006215107
ko00071_Fatty_acid_metabolism	Calprotectin...g.g.	-0.221535647	0.00681085	0.006222714
ko00941_Flavonoid_biosynthesis	Propionat...mol.g.	-0.222324646	0.00679985	0.006222714
ko03070_Bacterial_secretion_system	Beta.Defensin.2..ng.g.	0.221212214	0.0068944	0.006286818
ko00140_Steroid_hormone_biosynthesis	Valerat...mol.g.	0.221627386	0.006980259	0.006352775
ko00520_Amino_sugar_and_nucleotide_sugar_metabolism	Propionat...mol.g.	0.221097032	0.007120318	0.006457546
ko00906_Carotenoid_biosynthesis	Valerat...mol.g.	-0.22108742	0.00712288	0.006457546
ko00253_Tetracycline_biosynthesis	Caproat...mol.g.	-0.220876276	0.007179349	0.0064962
ko00400_Phenylalanine_tyrosine_and_tryptophan_biosynthesis	Caproat...mol.g.	-0.220159912	0.007373913	0.006659419
ko00362_Benzoate_degradation	Caproat...mol.g.	-0.220086197	0.007394197	0.00666492
ko04113_Meiosis_<>_yeast	Valerat...mol.g.	0.220012279	0.007414587	0.006670496
ko00785_Lipoic_acid_metabolism	Calprotectin...g.g.	-0.21913522	0.007452983	0.006679686
ko00281_Geraniol_degradation	Caproat...mol.g.	0.219872611	0.007453249	0.006679686
ko00340_Histidine_metabolism	Propionat...mol.g.	-0.219557323	0.007541185	0.006745621
ko00591_Linoleic_acid_metabolism	Beta.Defensin.2..ng.g.	-0.218706011	0.007573301	0.00676147
ko00590_Arachidonic_acid_metabolism	Chromogranin.A..nmol.g.	-0.218410527	0.00765713	0.006823341
ko04122_Sulfur_relay_system	Chromogranin.A..nmol.g.	0.218190265	0.007720153	0.006866472
ko02040_Flagellar_assembly	Beta.Defensin.2..ng.g.	-0.218130362	0.007737373	0.006868779
ko00360_Phenylalanine_metabolism	Caproat...mol.g.	-0.21852305	0.007836148	0.00694334
ko00790_Folate_biosynthesis	Butyrat...mol.g.	0.217600979	0.008107689	0.007170414
ko00941_Flavonoid_biosynthesis	Acetat...mol.g.	-0.217089114	0.008261999	0.007293151
ko00780_Biotin_metabolism	Chromogranin.A..nmol.g.	0.215778495	0.008440921	0.007437112
ko03018_RNA_degradation	Butyrat...mol.g.	0.216348597	0.008489842	0.007466207
ko00760_Nicotinate_and_nicotinamide_metabolism	Acetat...mol.g.	0.215483713	0.008762975	0.007692003

ko00521_Streptomycin_biosynthesis	Chromogranin.A..nmol.g.	0.214051571	0.008992997	0.007879186
ko04115_p53_signaling_pathway	Calprotectin...g.g.	-0.213316153	0.00923763	0.008078448
ko05012_Parkinson's_disease	Calprotectin...g.g.	-0.213082324	0.009316634	0.008132394
ko05145_Toxoplasmosis	Calprotectin...g.g.	-0.212913558	0.009374025	0.00816731
ko00940_Phenylpropanoid_biosynthesis	Butyrat...mol.g.	0.213469817	0.009429283	0.008200241
ko01053_Biosynthesis_of_siderophore_group_nonribosomal_peptides	Valerat...mol.g.	0.212941598	0.009611288	0.008343072
ko05143_African_trypanosomiasis	Propionat...mol.g.	-0.212682842	0.009701572	0.008405905
ko00564_Glycerophospholipid_metabolism	Acetat...mol.g.	-0.211295714	0.010198428	0.008803918
ko00620_Pyruvate_metabolism	Caproat...mol.g.	-0.211338621	0.010182729	0.008803918
ko00030_Pentose_phosphate_pathway	Acetat...mol.g.	-0.211082263	0.010276842	0.008855332
ko00620_Pyruvate_metabolism	Beta.Defensin.2..ng.g.	-0.210308195	0.010300468	0.008859434
ko00643_Styrene_degradation	Acetat...mol.g.	-0.210823477	0.010372622	0.008905184
ko00643_Styrene_degradation	Valerat...mol.g.	-0.210618083	0.010449199	0.008954558
ko00051_Fructose_and_mannose_metabolism	Calprotectin...g.g.	0.209418905	0.010634662	0.009096892
ko00623_Toluene_degradation	Caproat...mol.g.	0.209295378	0.010954379	0.00935334
ko00450_Selenocompound_metabolism	Chromogranin.A..nmol.g.	0.208374748	0.011039206	0.009391618
ko00311_Penicillin_and_cephalosporin_biosynthesis	Calprotectin...g.g.	-0.208423436	0.011020046	0.009391618
ko01053_Biosynthesis_of_siderophore_group_nonribosomal_peptides	Butyrat...mol.g.	0.208624627	0.011218672	0.00952704
ko04146_Peroxisome	Acetat...mol.g.	0.20834329	0.011331183	0.009605217
ko05110_Vibrio_cholerae_infection	Calprotectin...g.g.	-0.207120135	0.011543135	0.009767254
ko00290_Valine_leucine_and_isoleucine_biosynthesis	Caproat...mol.g.	-0.207652845	0.011611516	0.009807443
ko04976_Bile_secretion	Calprotectin...g.g.	-0.206874232	0.011644239	0.009817425
ko02040_Flagellar_assembly	Acetat...mol.g.	-0.20658468	0.01205721	0.010147389
ko00903_Limonene_and_pinene_degradation	Beta.Defensin.2..ng.g.	0.205828866	0.012082775	0.010150713
ko00130_Ubiquinone_and_other_terpenoid<>quinone_biosynthesis	Chromogranin.A..nmol.g.	-0.205452119	0.012244337	0.010268072
ko00040_Pentose_and_glucuronate_interconversions	Propionat...mol.g.	-0.206085277	0.012270686	0.010271826
ko00633_Nitrotoluene_degradation	Caproat...mol.g.	-0.205949828	0.012329154	0.010302405
ko00970_Aminoacyl<>tRNA_biosynthesis	Valerat...mol.g.	0.205676365	0.012447941	0.01038319
ko00053_Ascorbate_and_aldarate_metabolism	Acetat...mol.g.	-0.204572952	0.012937491	0.010772404
ko02040_Flagellar_assembly	Caproat...mol.g.	-0.204150413	0.013129374	0.010912826
ko03022_Basal_transcription_factors	Acetat...mol.g.	-0.203836906	0.013273348	0.011013002
ko02060_Phosphotransferase_system_[PTS]	Caproat...mol.g.	-0.203082483	0.013625483	0.011285232
ko04626_Plant<>pathogen_interaction	Caproat...mol.g.	-0.202649832	0.013831089	0.011435357
ko00072_Synthesis_and_degradation_of_ketone_bodies	Beta.Defensin.2..ng.g.	-0.201325474	0.014141821	0.011671717
ko00460_Cyanoamino_acid_metabolism	Propionat...mol.g.	0.201153209	0.014563377	0.011998554
ko04142_Lysosome	Butyrat...mol.g.	0.201093332	0.014593365	0.012002204
ko00983_Drug_metabolism_<>_other_enzymes	Chromogranin.A..nmol.g.	-0.19955522	0.015031504	0.012339108
ko00450_Selenocompound_metabolism	Calprotectin...g.g.	0.19950865	0.015055553	0.012339108
ko00340_Histidine_metabolism	Acetat...mol.g.	-0.199742914	0.015284153	0.012504639
ko00791_Atrazine_degradation	Caproat...mol.g.	0.199608403	0.015354496	0.012540342
ko05010_Alzheimer's_disease	Beta.Defensin.2..ng.g.	0.198571242	0.01554682	0.012675373
ko00750_Vitamin_B6_metabolism	Acetat...mol.g.	0.19899319	0.015679829	0.01276166
ko00983_Drug_metabolism_<>_other_enzymes	Acetat...mol.g.	0.198859075	0.015751542	0.012797847
ko05111_Vibrio_cholerae_pathogenic_cycle	Chromogranin.A..nmol.g.	0.197907701	0.015902946	0.012888101
ko00680_Methane_metabolism	Beta.Defensin.2..ng.g.	-0.197880833	0.015917515	0.012888101
ko00260_Glycine_serine_and_threonine_metabolism	Beta.Defensin.2..ng.g.	0.197636506	0.016050529	0.012928927
ko00940_Phenylpropanoid_biosynthesis	Beta.Defensin.2..ng.g.	-0.197660569	0.016037386	0.012928927
ko04976_Bile_secretion	Butyrat...mol.g.	-0.198406263	0.015995781	0.012928927
ko05110_Vibrio_cholerae_infection	Butyrat...mol.g.	-0.196910722	0.016826012	0.01353038
ko00630_Glyoxylate_and_dicarboxylate_metabolism	Propionat...mol.g.	-0.196453482	0.017087202	0.013716925
ko00562_Inositol_phosphate_metabolism	Calprotectin...g.g.	-0.19573741	0.017117796	0.013718035
ko00531_Glycosaminoglycan_degradation	Acetat...mol.g.	0.196314476	0.017167302	0.013734271
ko00960_Tropane_piperidine_and_pyridine_alkaloid_biosynthesis	Chromogranin.A..nmol.g.	-0.195096128	0.017491846	0.013949907
ko00523_Polyketide_sugar_unit_biosynthesis	Butyrat...mol.g.	0.195749456	0.017496249	0.013949907
ko03013_RNA_transport	Butyrat...mol.g.	-0.195689009	0.017531762	0.01395453
ko00561_Glycerolipid_metabolism	Caproat...mol.g.	-0.195451981	0.017671621	0.014042052
ko03410_Base_excision_repair	Valerat...mol.g.	0.194797406	0.018062895	0.014328717
ko01053_Biosynthesis_of_siderophore_group_nonribosomal_peptides	Acetat...mol.g.	0.194353749	0.018332341	0.014505748
ko00903_Limonene_and_pinene_degradation	Propionat...mol.g.	-0.194328405	0.018347838	0.014505748
ko00591_Linoleic_acid_metabolism	Acetat...mol.g.	-0.193564169	0.018820496	0.014854422
ko00590_Arachidonic_acid_metabolism	Acetat...mol.g.	0.193416831	0.018912822	0.014902247
ko00350_Tyrosine_metabolism	Chromogranin.A..nmol.g.	0.190446576	0.020422181	0.016064583
ko00591_Linoleic_acid_metabolism	Chromogranin.A..nmol.g.	0.190341072	0.020493315	0.016093582
ko00565_Ether_lipid_metabolism	Valerat...mol.g.	-0.19071718	0.02067522	0.016209327
ko04910_Insulin_signaling_pathway	Butyrat...mol.g.	-0.1906587	0.020714914	0.01621338
ko03022_Basal_transcription_factors	Propionat...mol.g.	-0.189739636	0.021347439	0.016680651
ko00621_Dioxin_degradation	Beta.Defensin.2..ng.g.	-0.18875744	0.021587041	0.016839853

ko00513_Various_types_of_N<>glycan_biosynthesis	Calprotectin...g.g.	-0.187880405	0.022214125	0.017271654
ko03015_mRNA_surveillance_pathway	Calprotectin...g.g.	-0.187880405	0.022214125	0.017271654
ko02030_Bacterial_chemotaxis	Acetat...mol.g.	-0.188416789	0.022287047	0.01729971
ko00500_Starch_and_sucrose_metabolism	Calprotectin...g.g.	0.187526616	0.022471489	0.017414094
ko03320_PPAR_signaling_pathway	Chromogranin.A..nmol.g.	-0.187475822	0.022508648	0.017414154
ko00240_Pyrimidine_metabolism	Propionat...mol.g.	0.187779389	0.02275235	0.017573746
ko00280_Valine_leucine_and_isoleucine_degradation	Caproat...mol.g.	0.187537959	0.022930763	0.017682468
ko02020_Two<>component_system	Acetat...mol.g.	-0.187353311	0.023068024	0.017759152
ko00363_Bisphenol_degradation	Chromogranin.A..nmol.g.	0.185695221	0.023845129	0.018326944
ko00473_D<>Alanine_metabolism	Chromogranin.A..nmol.g.	-0.185645246	0.023883601	0.018326944
ko00330_Arginine_and_proline_metabolism	Acetat...mol.g.	-0.18586671	0.024199076	0.01853873
ko00440_Phosphonate_and_phosphinate_metabolism	Butyrat...mol.g.	-0.184674466	0.02514024	0.019219009
ko00760_Nicotinate_and_nicotinamide_metabolism	Butyrat...mol.g.	0.18463875	0.025168912	0.019219009
ko00531_Glycosaminoglycan_degradation	Butyrat...mol.g.	0.184347675	0.025403622	0.01933794
ko00960_Tropane_piperidine_and_pyridine_alkaloid_biosynthesis	Valerat...mol.g.	0.18434348	0.025407018	0.01933794
ko00363_Bisphenol_degradation	Acetat...mol.g.	-0.183698199	0.025934096	0.019707172
ko00440_Phosphonate_and_phosphinate_metabolism	Acetat...mol.g.	-0.18362453	0.025994861	0.019721436
ko00480_Glutathione_metabolism	Chromogranin.A..nmol.g.	-0.182643027	0.026294665	0.019870634
ko05130_Pathogenic_Escherichia_coli_infection	Calprotectin...g.g.	-0.182535559	0.026384697	0.019870634
ko00350_Tyrosine_metabolism	Acetat...mol.g.	-0.183133404	0.026403083	0.019870634
ko00281_Geraniol_degradation	Butyrat...mol.g.	0.183261521	0.026296068	0.019870634
ko00770_Pantothenate_and_CoA_biosynthesis	Caproat...mol.g.	-0.183201974	0.026345761	0.019870634
ko00061_Fatty_acid_biosynthesis	Calprotectin...g.g.	-0.181602563	0.027177462	0.020420696
ko05111_Vibrio_cholerae_pathogenic_cycle	Calprotectin...g.g.	0.181386853	0.027363619	0.020527728
ko05100_Bacterial_invasion_of_epithelial_cells	Propionat...mol.g.	0.181877864	0.027471678	0.020575923
ko03060_Protein_export	Propionat...mol.g.	0.181619498	0.027696096	0.020710977
ko00622_Xylene_degradation	Beta.Defensin.2..ng.g.	-0.180720561	0.027945517	0.020864269
ko00281_Geraniol_degradation	Acetat...mol.g.	0.180088422	0.029058353	0.021660681
ko00720_Carbon_fixation_pathways_in_prokaryotes	Calprotectin...g.g.	-0.179327989	0.029195861	0.021728693
ko00061_Fatty_acid_biosynthesis	Chromogranin.A..nmol.g.	-0.178828577	0.029655726	0.022001208
ko00590_Arachidonic_acid_metabolism	Butyrat...mol.g.	0.179472262	0.029622461	0.022001208
ko00330_Arginine_and_proline_metabolism	Chromogranin.A..nmol.g.	0.178408083	0.030047689	0.022237093
ko00190_Oxidative_phosphorylation	Calprotectin...g.g.	-0.17833556	0.030115735	0.022237093
ko05131_Shigellosis	Calprotectin...g.g.	-0.178336866	0.030114508	0.022237093
ko04122_Sulfur_relay_system	Acetat...mol.g.	-0.178694379	0.030347917	0.022373356
ko04141_Protein_processing_in_endoplasmic_reticulum	Chromogranin.A..nmol.g.	-0.177208676	0.031190006	0.022958127
ko05150_Staphylococcus_aureus_infection	Calprotectin...g.g.	0.177112352	0.031283325	0.02299078
ko00720_Carbon_fixation_pathways_in_prokaryotes	Propionat...mol.g.	0.177295674	0.031690374	0.023253539
ko05142_Chagas_disease_[American_trypanosomiasis]	Valerat...mol.g.	0.177209009	0.031775186	0.023279398
ko00510_N<>Glycan_biosynthesis	Chromogranin.A..nmol.g.	-0.176329482	0.032050595	0.023444595
ko04622_RIG<>I<>like_receptor_signaling_pathway	Valerat...mol.g.	-0.176778177	0.032199672	0.023517012
ko05010_Alzheimer's_disease	Calprotectin...g.g.	-0.176035705	0.032342609	0.023584727
ko00643_Styrene_degradation	Beta.Defensin.2..ng.g.	-0.175856232	0.032522111	0.023651752
ko05146_Amoebiasis	Propionat...mol.g.	-0.176440976	0.032535252	0.023651752
ko03050_Proteasome	Calprotectin...g.g.	-0.175565881	0.0328143	0.023817739
ko00830_Retinol_metabolism	Chromogranin.A..nmol.g.	0.174885751	0.033507452	0.024245904
ko05010_Alzheimer's_disease	Valerat...mol.g.	0.175493346	0.033494237	0.024245904
ko00513_Various_types_of_N<>glycan_biosynthesis	Acetat...mol.g.	-0.174901995	0.034104717	0.024602267
ko03015_mRNA_surveillance_pathway	Acetat...mol.g.	-0.174901995	0.034104717	0.024602267
ko00625_Chloroalkane_and_chloroalkene_degradation	Valerat...mol.g.	-0.174284362	0.03475235	0.025008537
ko00650_Butanoate_metabolism	Caproat...mol.g.	-0.174263494	0.034774412	0.025008537
ko00062_Fatty_acid_elongation_in_mitochondria	Valerat...mol.g.	0.173300204	0.035805777	0.025671632
ko05322_Systemic_lupus_erythematosus	Valerat...mol.g.	0.173300204	0.035805777	0.025671632
ko00253_Tetracycline_biosynthesis	Beta.Defensin.2..ng.g.	-0.172515245	0.036021014	0.025747332
ko00650_Butanoate_metabolism	Calprotectin...g.g.	-0.172549868	0.035983188	0.025747332
ko00903_Limonene_and_pinene_degradation	Caproat...mol.g.	0.172552916	0.036623551	0.026138233
ko00523_Polyketide_sugar_unit_biosynthesis	Chromogranin.A..nmol.g.	0.171539257	0.037101193	0.026438946
ko00440_Phosphonate_and_phosphinate_metabolism	Chromogranin.A..nmol.g.	0.17148558	0.037161384	0.026441715
ko00363_Bisphenol_degradation	Beta.Defensin.2..ng.g.	-0.170264475	0.03855306	0.027390443
ko00523_Polyketide_sugar_unit_biosynthesis	Valerat...mol.g.	0.170470399	0.038986	0.027656191
ko00760_Nicotinate_and_nicotinamide_metabolism	Chromogranin.A..nmol.g.	-0.169688478	0.039224578	0.027775591
ko00633_Nitrotoluene_degradation	Chromogranin.A..nmol.g.	-0.1696476	0.039272606	0.027775591
ko00623_Toluene_degradation	Calprotectin...g.g.	-0.169551707	0.039385467	0.027798243
ko00791_Atrazine_degradation	Propionat...mol.g.	-0.170096856	0.039423022	0.027798243
ko04210_Apoptosis	Calprotectin...g.g.	-0.169275593	0.039711961	0.027918144
ko05010_Alzheimer's_disease	Butyrat...mol.g.	-0.169853662	0.03970975	0.027918144
ko00270_Cysteine_and_methionine_metabolism	Calprotectin...g.g.	0.167813998	0.041478331	0.029116343

ko00250_Alanine_aspartate_and_glutamate_metabolism	Propionat...mol.g.	0.168093776	0.041837304	0.029324496
ko00900_Terpenoid_backbone_biosynthesis	Propionat...mol.g.	0.167971779	0.041988261	0.029386444
ko02040_Flagellar_assembly	Butyrat...mol.g.	-0.167755024	0.042257595	0.029520959
ko03320_PPAR_signaling_pathway	Caproat...mol.g.	0.167716044	0.042306184	0.029520959
ko00410_beta<>Alanine_metabolism	Beta.Defensin.2..ng.g.	0.166941997	0.042563186	0.029656228
ko00523_Polyketide_sugar_unit_biosynthesis	Acetat...mol.g.	0.167453256	0.042634972	0.029662236
ko00072_Synthesis_and_degradation_of_ketone_bodies	Acetat...mol.g.	-0.166076213	0.044393017	0.030839663
ko00930_Caprolactam_degradation	Valerat...mol.g.	0.165982049	0.044515414	0.030879014
ko00280_Valine_leucine_and_isoleucine_degradation	Calprotectin...g.g.	-0.165350486	0.044604419	0.030895118
ko00230_Purine_metabolism	Acetat...mol.g.	0.165341413	0.04535563	0.031333833
ko00521_Streptomycin_biosynthesis	Butyrat...mol.g.	0.165329596	0.045371252	0.031333833
ko00270_Cysteine_and_methionine_metabolism	Chromogranin.A..nmol.g.	0.164603329	0.045590532	0.031439036
ko00473_D<>Alanine_metabolism	Beta.Defensin.2..ng.g.	0.16402303	0.046368909	0.031837121
ko04141_Protein_processing_in_endoplasmic_reticulum	Acetat...mol.g.	0.164580167	0.046371187	0.031837121
ko05010_Alzheimer's_disease	Acetat...mol.g.	-0.164678393	0.046239092	0.031837121
ko00621_Dioxin_degradation	Caproat...mol.g.	-0.164383724	0.04663631	0.031972403
ko00532_Glycosaminoglycan_biosynthesis_<>_chondroitin_sulfate	Beta.Defensin.2..ng.g.	-0.163558912	0.046999388	0.032174348
ko00270_Cysteine_and_methionine_metabolism	Valerat...mol.g.	-0.164004693	0.047151426	0.032231444
ko00903_Limonene_and_pinene_degradation	Valerat...mol.g.	0.163611308	0.047691047	0.03255293
ko00730_Thiamine_metabolism	Propionat...mol.g.	-0.163479838	0.047872532	0.032629381
ko00920_Sulfur_metabolism	Calprotectin...g.g.	0.16284	0.047990072	0.032662091
ko00410_beta<>Alanine_metabolism	Chromogranin.A..nmol.g.	-0.162258661	0.04880382	0.033167859
ko05146_Amoebiasis	Calprotectin...g.g.	-0.162172007	0.048926093	0.033202907
ko00640_Propanoate_metabolism	Calprotectin...g.g.	-0.161058182	0.050520538	0.034235478
ko00623_Toluene_degradation	Chromogranin.A..nmol.g.	-0.160900073	0.050750324	0.034341639
ko03013_RNA_transport	Chromogranin.A..nmol.g.	0.160668706	0.051088142	0.034470893
ko00340_Histidine_metabolism	Butyrat...mol.g.	-0.161221101	0.051081307	0.034470893
ko04626_Plant<>pathogen_interaction	Acetat...mol.g.	-0.161151881	0.05118239	0.034484938
ko00450_Selenocompound_metabolism	Valerat...mol.g.	0.160222378	0.052555878	0.035359616
ko01040_Biosynthesis_of_unsaturated_fatty_acids	Valerat...mol.g.	-0.160018362	0.052861389	0.035514284
ko00730_Thiamine_metabolism	Valerat...mol.g.	-0.159274081	0.053988416	0.036219647
ko00380_Tryptophan_metabolism	Beta.Defensin.2..ng.g.	0.158440527	0.054437871	0.036417128
ko00195_Photosynthesis	Caproat...mol.g.	-0.15898548	0.054430738	0.036417128
ko05143_African_trypanosomiasis	Valerat...mol.g.	0.158802333	0.054712984	0.036549105
ko05100_Bacterial_invasion_of_epithelial_cells	Acetat...mol.g.	0.158724132	0.054833866	0.036577825
ko00410_beta<>Alanine_metabolism	Butyrat...mol.g.	0.158261096	0.055554148	0.03695332
ko00592_alpha<>Linolenic_acid_metabolism	Caproat...mol.g.	-0.158308959	0.055479336	0.03695332
ko00051_Fructose_and_mannose_metabolism	Beta.Defensin.2..ng.g.	-0.156088099	0.0581691	0.038637997
ko00400_Phenylalanine_tyrosine_and_tryptophan_biosynthesis	Acetat...mol.g.	-0.156221577	0.058820205	0.038960271
ko00363_Bisphenol_degradation	Butyrat...mol.g.	-0.156222905	0.058818028	0.038960271
ko00564_Glycerophospholipid_metabolism	Caproat...mol.g.	-0.15605387	0.059095646	0.039087582
ko00311_Penicillin_and_cephalosporin_biosynthesis	Propionat...mol.g.	0.15599201	0.059197512	0.039099889
ko00362_Benzoate_degradation	Beta.Defensin.2..ng.g.	-0.154910742	0.060114045	0.039608788
ko00190_Oxidative_phosphorylation	Butyrat...mol.g.	0.155425763	0.060136676	0.039608788
ko01040_Biosynthesis_of_unsaturated_fatty_acids	Chromogranin.A..nmol.g.	-0.154645758	0.060559065	0.039804232
ko00930_Caprolactam_degradation	Calprotectin...g.g.	-0.154619726	0.06060293	0.039804232
ko00460_Cyanoamino_acid_metabolism	Calprotectin...g.g.	0.154232621	0.061258284	0.040178478
ko04146_Peroxisome	Calprotectin...g.g.	-0.154126357	0.061439198	0.040240934
ko00312_beta<>Lactam_resistance	Propionat...mol.g.	0.154237168	0.062147834	0.040648378
ko04210_Apoptosis	Acetat...mol.g.	-0.154005699	0.062545826	0.040851792
ko00020_Citrate_cycle_[TCA_cycle]	Calprotectin...g.g.	-0.153293466	0.062872406	0.040930319
ko00190_Oxidative_phosphorylation	Acetat...mol.g.	0.153784836	0.062927525	0.040930319
ko00640_Propanoate_metabolism	Valerat...mol.g.	-0.153797766	0.062905127	0.040930319
ko00330_Arginine_and_proline_metabolism	Butyrat...mol.g.	-0.153087227	0.064145676	0.04166494
ko05142_Chagas_disease_[American_trypanosomiasis]	Chromogranin.A..nmol.g.	-0.151971998	0.065202374	0.042292807
ko00622_Xylene_degradation	Caproat...mol.g.	-0.15217719	0.065763655	0.042598039
ko04122_Sulfur_relay_system	Butyrat...mol.g.	-0.15133238	0.067295313	0.04353012
ko00740_Riboflavin_metabolism	Beta.Defensin.2..ng.g.	0.150486942	0.067904385	0.043863681
ko00600_Sphingolipid_metabolism	Beta.Defensin.2..ng.g.	-0.149678071	0.069414016	0.044475937
ko00791_Atrazine_degradation	Beta.Defensin.2..ng.g.	0.149624394	0.069515155	0.044475937
ko00020_Citrate_cycle_[TCA_cycle]	Acetat...mol.g.	0.150313093	0.069181842	0.044475937
ko00513_Various_types_of_N<>glycan_biosynthesis	Propionat...mol.g.	-0.150171598	0.069447091	0.044475937
ko00062_Fatty_acid_elongation_in_mitochondria	Propionat...mol.g.	0.150149068	0.069489403	0.044475937
ko05322_Systemic_lupus_erythematosus	Propionat...mol.g.	0.150149068	0.069489403	0.044475937
ko03015_mRNA_surveillance_pathway	Propionat...mol.g.	-0.150171598	0.069447091	0.044475937
ko03060_Protein_export	Calprotectin...g.g.	-0.148982044	0.070734775	0.045133273
ko03320_PPAR_signaling_pathway	Calprotectin...g.g.	-0.149018098	0.070665863	0.045133273

ko00830_Retinol_metabolism	Beta.Defensin.2..ng.g.	0.148798864	0.07108574	0.045295668
ko00670_One_carbon_pool_by_folate	Acetat...mol.g.	0.149183361	0.071322781	0.045385129
ko00281_Geraniol_degradation	Chromogranin.A..nmol.g.	-0.148546922	0.071570759	0.045481298
ko00072_Synthesis_and_degradation_of_ketone_bodies	Butyrat...mol.g.	-0.14867272	0.072307969	0.045887681
ko05012_Parkinson's_disease	Caproat...mol.g.	0.148215092	0.07320022	0.046391226
ko00643_Styrene_degradation	Butyrat...mol.g.	-0.148134366	0.073358535	0.046428902
ko04141_Protein_processing_in_endoplasmic_reticulum	Butyrat...mol.g.	0.147864244	0.073890298	0.046702516
ko00983_Drug_metabolism_<>_other_enzymes	Butyrat...mol.g.	0.147683043	0.074248755	0.046866002
ko05111_Vibrio_cholerae_pathogenic_cycle	Caproat...mol.g.	-0.146903695	0.07580655	0.047785059
ko02060_Phosphotransferase_system_[PTS]	Calprotectin...g.g.	0.145913673	0.076802366	0.04834788
ko03320_PPAR_signaling_pathway	Propionat...mol.g.	0.145510184	0.07865767	0.049449528
ko00633_Nitrotoluene_degradation	Beta.Defensin.2..ng.g.	-0.144680473	0.079356332	0.049822058
ko03050_Proteasome	Butyrat...mol.g.	-0.145051078	0.079615691	0.049918154
