# Supplementary material for: The influence of a short-term gluten-free diet on the human gut microbiome
Source: Genome Med. 2016 Apr 21;8:45. doi: 10.1186/s13073-016-0295-y (PMC4841035; doi:10.1186/s13073-016-0295-y)
Supplement: Additional file 10: Table S5. — Correlation of predicted HUMAnN module activity and levels of fecal biomarkers. (PDF 143 kb) [file 13073_2016_295_MOESM10_ESM.pdf]

Keg_Module	Fecal_Biomarker	Cor(rho)	P-val	Q-val
M00185_Sulfate_transport_system	Chromogranin.A..nmol.g.	0.523766952	8.44E-12	9.60E-10
M00237_Branched<>chain_amino_acid_transport_system	Valerat...mol.g.	-0.527362217	6.76E-12	9.60E-10
M00050_Guanine_nucleotide_biosynthesis_IMP_=>_GDP/dGDPGTP/dGTP	Valerat...mol.g.	0.477106299	9.99E-10	5.54E-08
M00049_Adenine_nucleotide_biosynthesis_IMP_=>_ADP/dADPATP/dATP	Valerat...mol.g.	0.478934886	8.45E-10	5.54E-08
M00178_Ribosome_bacteria	Caproat...mol.g.	0.474949109	1.22E-09	5.54E-08
M00159_V<>type_ATPase_prokaryotes	Propionat...mol.g.	-0.467250669	2.43E-09	9.21E-08
M00003_Gluconeogenesis_oxaloacetate_=>_fructose<>6P	Chromogranin.A..nmol.g.	0.447217765	1.21E-08	3.06E-07
M00053_Pyrimidine_deoxyribonuleotide_biosynthesis_CDP/CTP_=>_dCDP/dCTPdTDP/dTTP	Chromogranin.A..nmol.g.	-0.449218627	1.02E-08	3.06E-07
M00200_Sorbitol/mannitol_transport_system	Chromogranin.A..nmol.g.	-0.447880221	1.14E-08	3.06E-07
M00144_Complex_I_[NADH_dehydrogenase]_NADH_dehydrogenase_I	Chromogranin.A..nmol.g.	-0.443917328	1.59E-08	3.30E-07
M00159_V<>type_ATPase_prokaryotes	Butyrat...mol.g.	-0.445923712	1.51E-08	3.30E-07
M00127_Thiamine_biosynthesis_AIR_=>_thiamine<>P/thiamine<>2P	Valerat...mol.g.	0.442289475	2.04E-08	3.87E-07
M00016_Lysine_biosynthesis_aspartate_=>_lysine	Chromogranin.A..nmol.g.	-0.435290329	3.22E-08	5.64E-07
M00061_Uronic_acid_metabolism	Beta.Defensin.2..ng.g.	-0.430848509	4.60E-08	7.48E-07
M00222_Phosphate_transport_system	Caproat...mol.g.	-0.428245059	6.27E-08	9.52E-07
M00133_Polyamine_biosynthesis_arginine_=>_agmatine_=>_putrescine_=>_spermidine	Chromogranin.A..nmol.g.	-0.422944581	8.55E-08	1.08E-06
M00159_V<>type_ATPase_prokaryotes	Acetat...mol.g.	-0.425001724	8.07E-08	1.08E-06
M00197_Putative_sugar_transport_system	Valerat...mol.g.	-0.425492165	7.77E-08	1.08E-06
M00061_Uronic_acid_metabolism	Chromogranin.A..nmol.g.	0.420980737	9.94E-08	1.19E-06
M00222_Phosphate_transport_system	Valerat...mol.g.	-0.418638193	1.31E-07	1.50E-06
M00123_Biotin_biosynthesis_pimeloyl<>CoA_=>_biotin	Propionat...mol.g.	0.414577086	1.79E-07	1.93E-06
M00299_Spermidine/putrescine_transport_system	Valerat...mol.g.	-0.414010604	1.86E-07	1.93E-06
M00018_Threonine_biosynthesis_apartate_=>_homoserine_=>_threonine	Chromogranin.A..nmol.g.	0.411418799	2.05E-07	1.95E-06
M00299_Spermidine/putrescine_transport_system	Caproat...mol.g.	-0.412960025	2.01E-07	1.95E-06
M00026_Histidine_biosynthesis_PRPP_=>_histidine	Valerat...mol.g.	-0.409920283	2.52E-07	2.30E-06
M00194_Maltose/maltodextrin_transport_system	Valerat...mol.g.	-0.407852346	2.94E-07	2.57E-06
M00011_Citrate_cycle_second_carbon_oxidation	Chromogranin.A..nmol.g.	-0.405328718	3.22E-07	2.71E-06
M00237_Branched<>chain_amino_acid_transport_system	Caproat...mol.g.	-0.398995659	5.56E-07	4.52E-06
M00009_Citrate_cycle_[TCA_cycle_Krebs_cycle]	Chromogranin.A..nmol.g.	-0.391794522	8.47E-07	6.57E-06
M00360_Aminoacyl<>tRNA_biosynthesis_prokaryotes	Caproat...mol.g.	0.392722279	8.65E-07	6.57E-06
M00064_ADP<>L<>glycero<>D<>manno<>heptose_biosynthesis	Propionat...mol.g.	0.388974154	1.12E-06	8.06E-06
M00359_Aminoacyl<>tRNA_biosynthesis_eukaryotes	Caproat...mol.g.	0.388826697	1.13E-06	8.06E-06
M00149_Complex_II_[succinate_dehydrogenase_/_fumarate_reductase]_succinate_dehydrogenase	Chromogranin.A..nmol.g.	-0.385918599	1.27E-06	8.77E-06
M00061_Uronic_acid_metabolism	Caproat...mol.g.	-0.383305594	1.65E-06	1.11E-05
M00049_Adenine_nucleotide_biosynthesis_IMP_=>_ADP/dADPATP/dATP	Chromogranin.A..nmol.g.	-0.38031751	1.86E-06	1.21E-05
M00007_Pentose_phosphate_pathway_non<>oxidative_phase_fructose_6P_=>_ribose_5P	Valerat...mol.g.	-0.380742788	1.96E-06	1.24E-05
M00050_Guanine_nucleotide_biosynthesis_IMP_=>_GDP/dGDPGTP/dGTP	Chromogranin.A..nmol.g.	-0.377829851	2.20E-06	1.35E-05
M00219_AI<>2_transport_system	Butyrat...mol.g.	-0.374752107	2.92E-06	1.75E-05
M00125_Riboflavin_biosynthesis_GTP_=>_riboflavin/FMN/FAD	Butyrat...mol.g.	0.373876775	3.09E-06	1.80E-05
M00064_ADP<>L<>glycero<>D<>manno<>heptose_biosynthesis	Acetat...mol.g.	0.371077956	3.71E-06	2.11E-05
M00157_F<>type_ATPase_bacteria	Chromogranin.A..nmol.g.	0.369100834	3.91E-06	2.12E-05
M00164_ATP_synthase	Chromogranin.A..nmol.g.	0.369100834	3.91E-06	2.12E-05
M00125_Riboflavin_biosynthesis_GTP_=>_riboflavin/FMN/FAD	Propionat...mol.g.	0.369096653	4.22E-06	2.23E-05
M00237_Branched<>chain_amino_acid_transport_system	Propionat...mol.g.	-0.368769864	4.31E-06	2.23E-05
M00125_Riboflavin_biosynthesis_GTP_=>_riboflavin/FMN/FAD	Acetat...mol.g.	0.367454956	4.69E-06	2.32E-05
M00310_Pyruvateferredoxin_oxidoreductase	Propionat...mol.g.	-0.36761571	4.64E-06	2.32E-05
M00026_Histidine_biosynthesis_PRPP_=>_histidine	Propionat...mol.g.	-0.359198855	7.90E-06	3.82E-05
M00159_V<>type_ATPase_prokaryotes	Valerat...mol.g.	-0.357430193	8.82E-06	4.18E-05
M00133_Polyamine_biosynthesis_arginine_=>_agmatine_=>_putrescine_=>_spermidine	Beta.Defensin.2..ng.g.	0.355364413	9.33E-06	4.33E-05
M00123_Biotin_biosynthesis_pimeloyl<>CoA_=>_biotin	Acetat...mol.g.	0.354417444	1.06E-05	4.83E-05
M00299_Spermidine/putrescine_transport_system	Chromogranin.A..nmol.g.	0.352440553	1.12E-05	4.99E-05
M00007_Pentose_phosphate_pathway_non<>oxidative_phase_fructose_6P_=>_ribose_5P	Chromogranin.A..nmol.g.	0.349506818	1.34E-05	5.68E-05
M00178_Ribosome_bacteria	Beta.Defensin.2..ng.g.	0.349117415	1.37E-05	5.68E-05
M00025_Tyrosine_biosynthesis_chorismate_=>_tyrosine	Acetat...mol.g.	0.350193759	1.37E-05	5.68E-05
M00119_Pantothenate_biosynthesis_valine/L<>aspartate_=>_pantothenate	Propionat...mol.g.	0.350930548	1.31E-05	5.68E-05
M00311_2<>oxoglutarateferredoxin_oxidoreductase	Caproat...mol.g.	0.349095921	1.47E-05	5.96E-05
M00179_Ribosome_archaea	Chromogranin.A..nmol.g.	-0.343193764	1.95E-05	7.80E-05
M00207_Multiple_sugar_transport_system	Chromogranin.A..nmol.g.	-0.341695865	2.13E-05	8.10E-05
M00310_Pyruvateferredoxin_oxidoreductase	Chromogranin.A..nmol.g.	-0.341906872	2.11E-05	8.10E-05
M00127_Thiamine_biosynthesis_AIR_=>_thiamine<>P/thiamine<>2P	Propionat...mol.g.	0.342809919	2.13E-05	8.10E-05
M00061_Uronic_acid_metabolism	Valerat...mol.g.	-0.34167941	2.28E-05	8.51E-05
M00184_RNA_polymerase_archaea	Chromogranin.A..nmol.g.	-0.339872883	2.38E-05	8.73E-05
M00197_Putative_sugar_transport_system	Propionat...mol.g.	-0.340665952	2.42E-05	8.74E-05
M00310_Pyruvateferredoxin_oxidoreductase	Acetat...mol.g.	-0.339660991	2.57E-05	9.13E-05
M00018_Threonine_biosynthesis_apartate_=>_homoserine_=>_threonine	Valerat...mol.g.	-0.33921044	2.64E-05	9.23E-05
M00064_ADP<>L<>glycero<>D<>manno<>heptose_biosynthesis	Butyrat...mol.g.	0.335207814	3.32E-05	0.000112472
M00025_Tyrosine_biosynthesis_chorismate_=>_tyrosine	Butyrat...mol.g.	0.335005695	3.36E-05	0.000112472
M00311_2<>oxoglutarateferredoxin_oxidoreductase	Valerat...mol.g.	0.335082896	3.35E-05	0.000112472
M00049_Adenine_nucleotide_biosynthesis_IMP_=>_ADP/dADPATP/dATP	Propionat...mol.g.	0.331636735	4.07E-05	0.000134343
M00237_Branched<>chain_amino_acid_transport_system	Beta.Defensin.2..ng.g.	-0.329884065	4.23E-05	0.000137611
M00050_Guanine_nucleotide_biosynthesis_IMP_=>_GDP/dGDPGTP/dGTP	Propionat...mol.g.	0.330620475	4.31E-05	0.000138295
M00049_Adenine_nucleotide_biosynthesis_IMP_=>_ADP/dADPATP/dATP	Beta.Defensin.2..ng.g.	0.329038175	4.44E-05	0.000140361
M00133_Polyamine_biosynthesis_arginine_=>_agmatine_=>_putrescine_=>_spermidine	Caproat...mol.g.	0.328640809	4.82E-05	0.00015038
M00197_Putative_sugar_transport_system	Beta.Defensin.2..ng.g.	-0.32471988	5.66E-05	0.000173544
M00016_Lysine_biosynthesis_aspartate_=>_lysine	Valerat...mol.g.	0.325592378	5.72E-05	0.000173544
M00050_Guanine_nucleotide_biosynthesis_IMP_=>_GDP/dGDPGTP/dGTP	Beta.Defensin.2..ng.g.	0.323524158	6.05E-05	0.000181147
M00048_Inosine_monophosphate_biosynthesis_PRPP_+_glutamine_=>_IMP	Valerat...mol.g.	0.323469102	6.43E-05	0.000190122
M00194_Maltose/maltodextrin_transport_system	Propionat...mol.g.	-0.321814155	7.05E-05	0.000205568

M00194_Maltose/maltodextrin_transport_system	Beta.Defensin.2..ng.g.	-0.320434902	7.17E-05	0.000206689
M00049_Adenine_nucleotide_biosynthesis_IMP_=>_ADP/dADPATP/dATP	Caproat...mol.g.	0.317857551	8.74E-05	0.000248622
M00025_Tyrosine_biosynthesis_chorismate_=>_tyrosine	Propionat...mol.g.	0.316904754	9.20E-05	0.000258515
M00050_Guanine_nucleotide_biosynthesis_IMP_=>_GDP/dGDPGTP/dGTP	Caproat...mol.g.	0.316218798	9.55E-05	0.000264969
M00178_Ribosome_bacteria	Chromogranin.A..nmol.g.	-0.311775474	0.000114623	0.000303329
M00077_Chondroitin_sulfate_degradation	Calprotectin...g.g.	-0.311970053	0.00011344	0.000303329
M00123_Biotin_biosynthesis_pimeloyl<>CoA_=>_biotin	Butyrat...mol.g.	0.312940719	0.000113757	0.000303329
M00123_Biotin_biosynthesis_pimeloyl<>CoA_=>_biotin	Valerat...mol.g.	0.312899945	0.000114003	0.000303329
M00088_Ketone_body_biosynthesis_acetyl<>CoA_=>_acetoacetate/3<>hydroxybutyrate/acetone	Chromogranin.A..nmol.g.	0.309419793	0.000129883	0.000339761
M00022_Shikimate_pathway_phosphoenolpyruvate_+_erythrose<>4P_=>_chorismate	Valerat...mol.g.	-0.309835928	0.000134045	0.000346665
M00119_Pantothenate_biosynthesis_valine/L<>aspartate_=>_pantothenate	Butyrat...mol.g.	0.308004858	0.000147543	0.000377286
M00064_ADP<>L<>glycero<>D<>manno<>heptose_biosynthesis	Valerat...mol.g.	0.306720908	0.000157752	0.000398909
M00246_Nickel_transport_system	Acetat...mol.g.	0.305914059	0.0001645	0.000410654
M00026_Histidine_biosynthesis_PRPP_=>_histidine	Caproat...mol.g.	-0.305738252	0.000166006	0.000410654
M00025_Tyrosine_biosynthesis_chorismate_=>_tyrosine	Valerat...mol.g.	0.304382281	0.000178056	0.000435727
M00185_Sulfate_transport_system	Caproat...mol.g.	-0.303063413	0.000190553	0.000461349
M00007_Pentose_phosphate_pathway_non<>oxidative_phase_fructose_6P_=>_ribose_5P	Beta.Defensin.2..ng.g.	-0.297849459	0.000236385	0.000560388
M00178_Ribosome_bacteria	Valerat...mol.g.	0.29887574	0.00023585	0.000560388
M00053_Pyrimidine_deoxyribonuleotide_biosynthesis_CDP/CTP_=>_dCDP/dCTPdTDP/dTTP	Valerat...mol.g.	0.298174908	0.000244343	0.000573284
M00310_Pyruvateferredoxin_oxidoreductase	Butyrat...mol.g.	-0.297451222	0.000253411	0.000588491
M00048_Inosine_monophosphate_biosynthesis_PRPP_+_glutamine_=>_IMP	Caproat...mol.g.	0.296060438	0.000271719	0.000624635
M00219_Al<>2_transport_system	Propionat...mol.g.	-0.293621646	0.000306815	0.000698262
M00222_Phosphate_transport_system	Chromogranin.A..nmol.g.	0.291920747	0.000318222	0.000710207
M00230_Glutamate/aspartate_transport_system	Calprotectin...g.g.	-0.291915438	0.000318305	0.000710207
M00077_Chondroitin_sulfate_degradation	Chromogranin.A..nmol.g.	-0.290033878	0.000349332	0.000759745
M00245_Cobalt_transport_system	Chromogranin.A..nmol.g.	0.289804856	0.000353294	0.000759745
M00012_Glyoxylate_cycle	Calprotectin...g.g.	-0.289772282	0.000353861	0.000759745
M00053_Pyrimidine_deoxyribonuleotide_biosynthesis_CDP/CTP_=>_dCDP/dCTPdTDP/dTTP	Caproat...mol.g.	0.290764261	0.000353261	0.000759745
M00239_Peptides/nickel_transport_system	Valerat...mol.g.	-0.289589239	0.000374184	0.000795871
M00299_Spermidine/putrescine_transport_system	Beta.Defensin.2..ng.g.	-0.284342987	0.000461031	0.00097151
M00219_Al<>2_transport_system	Acetat...mol.g.	-0.284731457	0.000473437	0.0009885
M00126_Tetrahydrofolate_biosynthesis_GTP_=>_THF	Valerat...mol.g.	0.280988723	0.000565908	0.00117083
M00012_Glyoxylate_cycle	Chromogranin.A..nmol.g.	-0.279620396	0.00057785	0.001184767
M00311_2<>oxoglutarateferredoxin_oxidoreductase	Beta.Defensin.2..ng.g.	0.276687407	0.000663543	0.001348317
M00246_Nickel_transport_system	Propionat...mol.g.	0.27721774	0.000675667	0.001360803
M00007_Pentose_phosphate_pathway_non<>oxidative_phase_fructose_6P_=>_ribose_5P	Propionat...mol.g.	-0.276868297	0.000686771	0.001371034
M00016_Lysine_biosynthesis_aspartate_=>_lysine	Caproat...mol.g.	0.276505033	0.000698492	0.001382308
M00125_Riboflavin_biosynthesis_GTP_=>_riboflavin/FMN/FAD	Valerat...mol.g.	0.276273423	0.000706061	0.001385241
M00026_Histidine_biosynthesis_PRPP_=>_histidine	Chromogranin.A..nmol.g.	0.272787597	0.000795618	0.001521594
M00222_Phosphate_transport_system	Beta.Defensin.2..ng.g.	-0.272944088	0.000789884	0.001521594
M00222_Phosphate_transport_system	Propionat...mol.g.	-0.273859446	0.000789564	0.001521594
M00008_Entner<>Doudoroff_pathway_glucose<>6P_=>_glyceraldehyde<>3P_+_pyruvate	Calprotectin...g.g.	-0.272079029	0.000822063	0.001559066
M00127_Thiamine_biosynthesis_AIR_=>_thiamine<>P/thiamine<>2P	Butyrat...mol.g.	0.272161786	0.000853619	0.001605535
M00026_Histidine_biosynthesis_PRPP_=>_histidine	Acetat...mol.g.	-0.271927389	0.000862829	0.001609555
M00246_Nickel_transport_system	Butyrat...mol.g.	0.27086232	0.000905835	0.001676042
M00127_Thiamine_biosynthesis_AIR_=>_thiamine<>P/thiamine<>2P	Caproat...mol.g.	0.269492608	0.000964032	0.001769337
M00127_Thiamine_biosynthesis_AIR_=>_thiamine<>P/thiamine<>2P	Beta.Defensin.2..ng.g.	0.266679253	0.001051668	0.001899543
M00276_PTS_system_mannose<>specific_II_component	Acetat...mol.g.	-0.267584443	0.001050826	0.001899543
M00360_Aminoacyl<>tRNA_biosynthesis_prokaryotes	Beta.Defensin.2..ng.g.	0.265131849	0.001127539	0.002020547
M00026_Histidine_biosynthesis_PRPP_=>_histidine	Beta.Defensin.2..ng.g.	-0.26366428	0.00120409	0.002091842
M00276_PTS_system_mannose<>specific_II_component	Propionat...mol.g.	-0.264965975	0.001181591	0.002091842
M00319_Manganese/zinc/iron_transport_system	Butyrat...mol.g.	0.264667913	0.001197383	0.002091842
M00018_Threonine_biosynthesis_apartate_=>_homoserine_=>_threonine	Caproat...mol.g.	-0.264788045	0.001190995	0.002091842
M00096_C5_isoprenoid_biosynthesis_non<>mevalonate_pathway	Valerat...mol.g.	0.263794451	0.00124478	0.002130011
M00121_Heme_biosynthesis_glutamate_=>_protoheme/siroheme	Valerat...mol.g.	0.263843565	0.00124207	0.002130011
M00319_Manganese/zinc/iron_transport_system	Beta.Defensin.2..ng.g.	0.260341634	0.001395264	0.002369696
M00127_Thiamine_biosynthesis_AIR_=>_thiamine<>P/thiamine<>2P	Acetat...mol.g.	0.259256076	0.001519803	0.00256209
M00185_Sulfate_transport_system	Valerat...mol.g.	-0.259022745	0.001535337	0.002569246
M00193_Putative_spermidine/putrescine_transport_system	Beta.Defensin.2..ng.g.	-0.257744658	0.001563543	0.002597348
M00198_sn<>Glycerol_3<>phosphate_transport_system	Calprotectin...g.g.	-0.256465176	0.001653075	0.002726179
M00018_Threonine_biosynthesis_apartate_=>_homoserine_=>_threonine	Beta.Defensin.2..ng.g.	-0.25526807	0.001741044	0.002850597
M00008_Entner<>Doudoroff_pathway_glucose<>6P_=>_glyceraldehyde<>3P_+_pyruvate	Chromogranin.A..nmol.g.	-0.254651208	0.001788018	0.002865659
M00051_Uridine_monophosphate_biosynthesis_glutamine_[+_PRPP]_=>_UMP	Chromogranin.A..nmol.g.	0.254885085	0.001770074	0.002865659
M00359_Aminoacyl<>tRNA_biosynthesis_eukaryotes	Beta.Defensin.2..ng.g.	0.254666508	0.001786839	0.002865659
M00018_Threonine_biosynthesis_apartate_=>_homoserine_=>_threonine	Propionat...mol.g.	-0.252464854	0.002035688	0.003239785
M00003_Gluconeogenesis_oxaloacetate_=>_fructose<>6P	Calprotectin...g.g.	0.25135976	0.002058719	0.003253686
M00245_Cobalt_transport_system	Beta.Defensin.2..ng.g.	-0.25009278	0.002172477	0.003409794
M00185_Sulfate_transport_system	Calprotectin...g.g.	0.249904324	0.002189877	0.003413562
M00230_Glutamate/aspartate_transport_system	Acetat...mol.g.	0.249674862	0.002290281	0.003545785
M00119_Pantothenate_biosynthesis_valine/L<>aspartate_=>_pantothenate	Acetat...mol.g.	0.249104875	0.002345716	0.003582862
M00121_Heme_biosynthesis_glutamate_=>_protoheme/siroheme	Caproat...mol.g.	0.249114991	0.002344722	0.003582862
M00319_Manganese/zinc/iron_transport_system	Propionat...mol.g.	0.248095529	0.002446872	0.003712453
M00003_Gluconeogenesis_oxaloacetate_=>_fructose<>6P	Butyrat...mol.g.	0.24721803	0.002538008	0.003825224
M00246_Nickel_transport_system	Beta.Defensin.2..ng.g.	0.246028981	0.00257688	0.003858261
M00022_Shikimate_pathway_phosphoenolpyruvate_+_erythrose<>4P_=>_chorismate	Chromogranin.A..nmol.g.	0.24562855	0.002620206	0.003897489
M00277_PTS_system_N<>acetylgalactosamine<>specific_II_component	Chromogranin.A..nmol.g.	-0.245399034	0.002645335	0.003909316
M00310_Pyruvateferredoxin_oxidoreductase	Calprotectin...g.g.	-0.244989616	0.002690702	0.003940024
M00231_Octopine/nopaline_transport_system	Valerat...mol.g.	0.245719772	0.002700739	0.003940024
M00174_Methane_oxidation_methylotroph_methane_=>_CO2	Valerat...mol.g.	0.245543329	0.002720511	0.00394359

M00335_Sec_[secretion]_system	Calprotectin...g.g.	-0.244091098	0.002792744	0.004022675
M00117_Ubiquinone_biosynthesis_prokaryotes_chorismate_=>_ubiquinone	Calprotectin...g.g.	-0.243751419	0.002832222	0.004053882
M00276_PTS_system_mannose<>specific_II_component	Calprotectin...g.g.	0.243556951	0.00285505	0.004061016
M00096_C5_isoprenoid_biosynthesis_non<>mevalonate_pathway	Propionat...mol.g.	0.243955099	0.002904468	0.004072895
M00051_Uridine_monophosphate_biosynthesis_glutamine_[+_PRPP]_=>_UMP	Propionat...mol.g.	0.243949433	0.002905144	0.004072895
M00184_RNA_polymerase_archaea	Butyrat...mol.g.	-0.243849483	0.002917091	0.004072895
M00179_Ribosome_archaea	Butyrat...mol.g.	-0.243433538	0.002967285	0.004117715
M00349_Microcin_C_transport_system	Calprotectin...g.g.	-0.239557109	0.00336299	0.004638553
M00237_Branched<>chain_amino_acid_transport_system	Chromogranin.A..nmol.g.	0.239305751	0.003397476	0.004657891
M00124_Pyridoxal_biosynthesis_erythrose<>4P_=>_pyridoxal<>5P	Calprotectin...g.g.	-0.238885251	0.003455882	0.004709593
M00200_Sorbitol/mannitol_transport_system	Calprotectin...g.g.	-0.23833009	0.003534377	0.004787894
M00121_Heme_biosynthesis_glutamate_=>_protoheme/siroheme	Chromogranin.A..nmol.g.	-0.237780579	0.00361365	0.004837691
M00016_Lysine_biosynthesis_aspartate_=>_lysine	Calprotectin...g.g.	-0.237822493	0.003607548	0.004837691
M00197_Putative_sugar_transport_system	Caproat...mol.g.	-0.236619265	0.003908459	0.005201761
M00026_Histidine_biosynthesis_PRPP_=>_histidine	Butyrat...mol.g.	-0.236360494	0.00394898	0.005225134
M00239_Peptides/nickel_transport_system	Propionat...mol.g.	-0.234791766	0.004202808	0.005528845
M00007_Pentose_phosphate_pathway_non<>oxidative_phase_fructose_6P_=>_ribose_5P	Butyrat...mol.g.	-0.232639014	0.004574963	0.005983831
M00260_DNA_polymerase_III_complex_bacteria	Calprotectin...g.g.	-0.231489857	0.004642093	0.006036939
M00336_Twin<>arginine_translocation_[Tat]_system	Calprotectin...g.g.	-0.231222204	0.00469116	0.006066085
M00232_General_L<>amino_acid_transport_system	Calprotectin...g.g.	-0.229772329	0.004965117	0.006384063
M00149_Complex_II_[succinate_dehydrogenase_/_fumarate_reductase]_succinate_dehydrogenase	Butyrat...mol.g.	-0.230078408	0.005055898	0.006464266
M00232_General_L<>amino_acid_transport_system	Acetat...mol.g.	0.229308008	0.005209193	0.006549872
M00125_Riboflavin_biosynthesis_GTP_=>_riboflavin/FMN/FAD	Caproat...mol.g.	0.229533124	0.005163975	0.006549872
M00239_Peptides/nickel_transport_system	Caproat...mol.g.	-0.229410265	0.00518861	0.006549872
M00207_Multiple_sugar_transport_system	Propionat...mol.g.	-0.228920886	0.005287781	0.006592828
M00193_Putative_spermidine/putrescine_transport_system	Valerat...mol.g.	-0.228854838	0.005301294	0.006592828
M00225_Lysine/arginine/ornithine_transport_system	Calprotectin...g.g.	-0.227089743	0.005509995	0.006815133
M00115_NAD_biosynthesis_aspartate_=>_NAD	Acetat...mol.g.	-0.226845739	0.005727389	0.007045729
M00149_Complex_II_[succinate_dehydrogenase_/_fumarate_reductase]_succinate_dehydrogenase	Calprotectin...g.g.	-0.224940743	0.005984379	0.007322292
M00096_C5_isoprenoid_biosynthesis_non<>mevalonate_pathway	Butyrat...mol.g.	0.225075606	0.006127856	0.007457751
M00348_Glutathione_transport_system	Butyrat...mol.g.	0.224845152	0.006181788	0.007483369
M00053_Pyrimidine_deoxyribonuleotide_biosynthesis_CDP/CTP_=>_dCDP/dCTPdTDP/dTTP	Beta.Defensin.2..ng.g.	0.223544426	0.006311808	0.007600338
M00185_Sulfate_transport_system	Beta.Defensin.2..ng.g.	-0.222183969	0.006646081	0.007960731
M00022_Shikimate_pathway_phosphoenolpyruvate_+_erythrose<>4P_=>_chorismate	Propionat...mol.g.	-0.221810849	0.006932382	0.00826019
M00319_Manganese/zinc/iron_transport_system	Acetat...mol.g.	0.221515223	0.007009674	0.008265734
M00004_Pentose_phosphate_pathway_[Pentose_phosphate_cycle]	Caproat...mol.g.	-0.221577518	0.006993323	0.008265734
M00194_Maltose/maltodextrin_transport_system	Caproat...mol.g.	-0.221290217	0.007069014	0.00829274
M00226_Histidine_transport_system	Calprotectin...g.g.	-0.220150962	0.007174981	0.008373887
M00299_Spermidine/putrescine_transport_system	Propionat...mol.g.	-0.218741293	0.00777307	0.009025628
M00197_Putative_sugar_transport_system	Chromogranin.A..nmol.g.	0.217721978	0.007855675	0.009075242
M00231_Octopine/nopaline_transport_system	Acetat...mol.g.	0.217800819	0.008048141	0.00925063
M00246_Nickel_transport_system	Valerat...mol.g.	0.217477094	0.008144801	0.009268115
M00119_Pantothenate_biosynthesis_valine/L<>aspartate_=>_pantothenate	Valerat...mol.g.	0.217539512	0.008126084	0.009268115
M00006_Pentose_phosphate_pathway_oxidative_phase_glucose_6P_=>_ribulose_5P	Caproat...mol.g.	-0.21719806	0.00822894	0.009317272
M00174_Methane_oxidation_methylotroph_methane_=>_CO2	Acetat...mol.g.	0.216703857	0.008379851	0.009441171
M00348_Glutathione_transport_system	Calprotectin...g.g.	-0.215133244	0.008643587	0.009560076
M00157_F<>type_ATPase_bacteria	Acetat...mol.g.	0.215967079	0.008609383	0.009560076
M00348_Glutathione_transport_system	Acetat...mol.g.	0.215740406	0.008681111	0.009560076
M00164_ATP_synthase	Acetat...mol.g.	0.215967079	0.008609383	0.009560076
M00157_F<>type_ATPase_bacteria	Valerat...mol.g.	-0.21556358	0.008737431	0.009560076
M00164_ATP_synthase	Valerat...mol.g.	-0.21556358	0.008737431	0.009560076
M00133_Polyamine_biosynthesis_arginine_=>_agmatine_=>_putrescine_=>_spermidine	Acetat...mol.g.	-0.215166166	0.00886519	0.009653453
M00095_C5_isoprenoid_biosynthesis_mevalonate_pathway	Chromogranin.A..nmol.g.	-0.214116121	0.008971801	0.009676941
M00311_2<>oxoglutarateferredoxin_oxidoreductase	Propionat...mol.g.	0.214885931	0.008956269	0.009676941
M00149_Complex_II_[succinate_dehydrogenase_/_fumarate_reductase]_succinate_dehydrogenase	Acetat...mol.g.	-0.214459822	0.009096344	0.009764993
M00133_Polyamine_biosynthesis_arginine_=>_agmatine_=>_putrescine_=>_spermidine	Butyrat...mol.g.	-0.214068618	0.009226644	0.00985837
M00144_Complex_I_[NADH_dehydrogenase]_NADH_dehydrogenase_I	Calprotectin...g.g.	-0.213077792	0.009318171	0.009909639
M00025_Tyrosine_biosynthesis_chorismate_=>_tyrosine	Beta.Defensin.2..ng.g.	0.212120287	0.00964799	0.010132361
M00300_Putrescine_transport_system	Calprotectin...g.g.	-0.212082646	0.009661164	0.010132361
M00245_Cobalt_transport_system	Valerat...mol.g.	-0.212832034	0.009649426	0.010132361
M00200_Sorbitol/mannitol_transport_system	Beta.Defensin.2..ng.g.	0.211899296	0.009725559	0.010153108
M00194_Maltose/maltodextrin_transport_system	Chromogranin.A..nmol.g.	0.210977165	0.010055201	0.010449309
M00233_Glutamate_transport_system	Butyrat...mol.g.	0.21146696	0.0101359	0.010485293
M00230_Glutamate/aspartate_transport_system	Butyrat...mol.g.	0.210647807	0.010438087	0.010749037
M00197_Putative_sugar_transport_system	Butyrat...mol.g.	-0.208941973	0.011092939	0.011371941
M00239_Peptides/nickel_transport_system	Beta.Defensin.2..ng.g.	-0.206902424	0.011632609	0.011866726
M00034_Methionine_salvage_pathway	Calprotectin...g.g.	-0.20666227	0.011732012	0.011866726
M00144_Complex_I_[NADH_dehydrogenase]_NADH_dehydrogenase_I	Acetat...mol.g.	-0.207426253	0.011704834	0.011866726
M00194_Maltose/maltodextrin_transport_system	Butyrat...mol.g.	-0.206660106	0.012025253	0.012109515
M00011_Citrate_cycle_second_carbon_oxidation	Calprotectin...g.g.	-0.205463422	0.012239463	0.012270929
M00276_PTS_system_mannose<>specific_II_component	Valerat...mol.g.	-0.205748148	0.012416663	0.012339863
M00319_Manganese/zinc/iron_transport_system	Valerat...mol.g.	0.205771666	0.012406431	0.012339863
M00157_F<>type_ATPase_bacteria	Butyrat...mol.g.	0.205024264	0.012735257	0.012546907
M00164_ATP_synthase	Butyrat...mol.g.	0.205024264	0.012735257	0.012546907
M00125_Riboflavin_biosynthesis_GTP_=>_riboflavin/FMN/FAD	Beta.Defensin.2..ng.g.	0.202926557	0.013377121	0.013041543
M00035_Methionine_degradation	Calprotectin...g.g.	-0.202857736	0.013409231	0.013041543
M00206_Cellobiose_transport_system	Butyrat...mol.g.	0.203717324	0.013328628	0.013041543
M00009_Citrate_cycle_[TCA_cycle_Krebs_cycle]	Calprotectin...g.g.	-0.20218114	0.013728523	0.013295262
M00202_Oligogalacturonide_transport_system	Calprotectin...g.g.	-0.201728221	0.013945955	0.01333559

M00276_PTS_system_mannose<>specific_II_component	Butyrat...mol.g.	-0.202468497	0.013918067	0.01333559
M00001_Glycolysis_[Embden<>Meyerhof_pathway]_glucose_=>_pyruvate	Butyrat...mol.g.	-0.202485497	0.013909893	0.01333559
M00197_Putative_sugar_transport_system	Acetat...mol.g.	-0.20183209	0.014227115	0.013443381
M00231_Octopine/nopaline_transport_system	Propionat...mol.g.	0.201965043	0.014162061	0.013443381
M00159_V<>type_ATPase_prokaryotes	Caproat...mol.g.	-0.201814198	0.014235889	0.013443381
M00174_Methane_oxidation_methylotroph_methane_=>_CO2	Propionat...mol.g.	0.201550803	0.01436561	0.013509823
M00095_C5_isoprenoid_biosynthesis_mevalonate_pathway	Calprotectin...g.g.	-0.199946489	0.014830769	0.013782546
M00245_Cobalt_transport_system	Calprotectin...g.g.	0.199933706	0.01483729	0.013782546
M00003_Gluconeogenesis_oxaloacetate_=>_fructose<>6P	Propionat...mol.g.	0.200822641	0.014729609	0.013782546
M00231_Octopine/nopaline_transport_system	Butyrat...mol.g.	0.199553043	0.015383528	0.014231864
M00174_Methane_oxidation_methylotroph_methane_=>_CO2	Butyrat...mol.g.	0.199154144	0.01559414	0.014368301
M00018_Threonine_biosynthesis_apartate_=>_homoserine_=>_threonine	Calprotectin...g.g.	0.197745807	0.015990905	0.014674466
M00019_Leucine_biosynthesis_pyruvate_=>_2<>oxoisovalerate_=>_leucine	Calprotectin...g.g.	-0.197481912	0.016135191	0.014747408
M00136_GABA_biosynthesis_prokaryotes_putrescine_=>_GABA	Calprotectin...g.g.	-0.197153396	0.016316389	0.014853369
M00126_Tetrahydrofolate_biosynthesis_GTP_=>_THF	Propionat...mol.g.	0.197621045	0.01642713	0.014894602
M00287_PTS_system_galactosamine<>specific_II_component	Calprotectin...g.g.	-0.196614237	0.016617606	0.015007517
M00003_Gluconeogenesis_oxaloacetate_=>_fructose<>6P	Acetat...mol.g.	0.196888716	0.016838502	0.015128642
M00002_Glycolysis_core_module_involving_three<>carbon_compounds	Acetat...mol.g.	-0.196807491	0.016884676	0.015128642
M00007_Pentose_phosphate_pathway_non<>oxidative_phase_fructose_6P_=>_ribose_5P	Acetat...mol.g.	-0.19657515	0.01701736	0.015187733
M00002_Glycolysis_core_module_involving_three<>carbon_compounds	Caproat...mol.g.	0.196327117	0.017160004	0.015255216
M00133_Polyamine_biosynthesis_arginine_=>_agmatine_=>_putrescine_=>_spermidine	Valerat...mol.g.	0.195770259	0.017484041	0.015482804
M00185_Sulfate_transport_system	Butyrat...mol.g.	0.19543211	0.01768339	0.015598641
M00194_Maltose/maltodextrin_transport_system	Acetat...mol.g.	-0.195060215	0.017904907	0.015733062
M00095_C5_isoprenoid_biosynthesis_mevalonate_pathway	Beta.Defensin.2..ng.g.	-0.193422377	0.018501708	0.016194943
M00319_Manganese/zinc/iron_transport_system	Calprotectin...g.g.	0.192994142	0.018768058	0.016365142
M00157_F<>type_ATPase_bacteria	Calprotectin...g.g.	0.192343424	0.019179137	0.016471158
M00164_ATP_synthase	Calprotectin...g.g.	0.192343424	0.019179137	0.016471158
M00049_Adenine_nucleotide_biosynthesis_IMP_=>_ADP/dADPATP/dATP	Acetat...mol.g.	0.193250604	0.019017455	0.016471158
M00232_General_L<>amino_acid_transport_system	Butyrat...mol.g.	0.193065192	0.019134754	0.016471158
M00009_Citrate_cycle_[TCA_cycle_Krebs_cycle]	Butyrat...mol.g.	-0.192488728	0.019503457	0.016686717
M00144_Complex_I_[NADH_dehydrogenase]_NADH_dehydrogenase_I	Butyrat...mol.g.	-0.191645728	0.020053686	0.017093221
M00022_Shikimate_pathway_phosphoenolpyruvate_+_erythrose<>4P_=>_chorismate	Acetat...mol.g.	-0.190470074	0.020843397	0.017700056
M00002_Glycolysis_core_module_involving_three<>carbon_compounds	Butyrat...mol.g.	-0.189262757	0.021682151	0.018327327
M00200_Sorbitol/mannitol_transport_system	Caproat...mol.g.	0.189176569	0.021743124	0.018327327
M00051_Uridine_monophosphate_biosynthesis_glutamine_[+_PRPP]_=>_UMP	Acetat...mol.g.	0.189040141	0.021839942	0.018341005
M00117_Ubiquinone_biosynthesis_prokaryotes_chorismate_=>_ubiquinone	Chromogranin.A..nmol.g.	-0.188113382	0.022046034	0.018435151
M00001_Glycolysis_[Embden<>Meyerhof_pathway]_glucose_=>_pyruvate	Acetat...mol.g.	-0.188656685	0.022114055	0.018435151
M00077_Chondroitin_sulfate_degradation	Caproat...mol.g.	0.188340577	0.022342247	0.018557404
M00050_Guanine_nucleotide_biosynthesis_IMP_=>_GDP/dGDPGTP/dGTP	Acetat...mol.g.	0.187783991	0.02274896	0.018826509
M00360_Aminoacyl<>tRNA_biosynthesis_prokaryotes	Valerat...mol.g.	0.183342291	0.026228792	0.021627691
M00200_Sorbitol/mannitol_transport_system	Acetat...mol.g.	-0.182821453	0.026665211	0.021908175
M00126_Tetrahydrofolate_biosynthesis_GTP_=>_THF	Caproat...mol.g.	0.182002851	0.027363672	0.022401161
M00157_F<>type_ATPase_bacteria	Caproat...mol.g.	-0.181459263	0.027836062	0.02262511
M00164_ATP_synthase	Caproat...mol.g.	-0.181459263	0.027836062	0.02262511
M00324_Dipeptide_transport_system	Calprotectin...g.g.	-0.179963105	0.028619823	0.023179367
M00237_Branched<>chain_amino_acid_transport_system	Butyrat...mol.g.	-0.179534597	0.029564971	0.023859937
M00331_Type_II_general_secretion_system	Valerat...mol.g.	0.178051129	0.030959172	0.024896818
M00049_Adenine_nucleotide_biosynthesis_IMP_=>_ADP/dADPATP/dATP	Calprotectin...g.g.	-0.177303282	0.031098583	0.024920871
M00022_Shikimate_pathway_phosphoenolpyruvate_+_erythrose<>4P_=>_chorismate	Butyrat...mol.g.	-0.177734416	0.031263954	0.024965484
M00126_Tetrahydrofolate_biosynthesis_GTP_=>_THF	Beta.Defensin.2..ng.g.	0.17661529	0.031768652	0.025191721
M00050_Guanine_nucleotide_biosynthesis_IMP_=>_GDP/dGDPGTP/dGTP	Calprotectin...g.g.	-0.176622054	0.031762005	0.025191721
M00060_Lipopolysaccharide_biosynthesis_KDO2<>lipid_A	Calprotectin...g.g.	-0.175985333	0.032392903	0.02544974
M00207_Multiple_sugar_transport_system	Acetat...mol.g.	-0.176650538	0.03232635	0.02544974
M00115_NAD_biosynthesis_aspartate_=>_NAD	Butyrat...mol.g.	-0.176465199	0.032511046	0.02544974
M00200_Sorbitol/mannitol_transport_system	Butyrat...mol.g.	-0.176434888	0.032541337	0.02544974
M00009_Citrate_cycle_[TCA_cycle_Krebs_cycle]	Acetat...mol.g.	-0.174963355	0.034040937	0.026531365
M00150_Complex_II_[succinate_dehydrogenase_/_fumarate_reductase]_fumarate_reductase	Calprotectin...g.g.	-0.17386642	0.034569488	0.026851359
M00207_Multiple_sugar_transport_system	Calprotectin...g.g.	-0.17336013	0.035107478	0.027176483
M00149_Complex_II_[succinate_dehydrogenase_/_fumarate_reductase]_succinate_dehydrogenase	Propionat...mol.g.	-0.173316662	0.035787941	0.027609316
M00004_Pentose_phosphate_pathway_[Pentose_phosphate_cycle]	Beta.Defensin.2..ng.g.	-0.172435654	0.036108097	0.027741914
M00342_Bacterial_proteasome	Valerat...mol.g.	0.172823622	0.036325511	0.027741914
M00011_Citrate_cycle_second_carbon_oxidation	Caproat...mol.g.	0.172850248	0.036296307	0.027741914
M00179_Ribosome_archaea	Acetat...mol.g.	-0.172523789	0.036655741	0.027900486
M00184_RNA_polymerase_archaea	Acetat...mol.g.	-0.172369822	0.036826299	0.027936871
M00049_Adenine_nucleotide_biosynthesis_IMP_=>_ADP/dADPATP/dATP	Butyrat...mol.g.	0.172258312	0.036950243	0.02793777
M00185_Sulfate_transport_system	Acetat...mol.g.	0.171688275	0.037589342	0.028233391
M00035_Methionine_degradation	Valerat...mol.g.	0.171755517	0.037513472	0.028233391
M00277_PTS_system_N<>acetylgalactosamine<>specific_II_component	Valerat...mol.g.	0.171515036	0.037785405	0.028287296
M00064_ADP<>L<>glycero<>D<>manno<>heptose_biosynthesis	Beta.Defensin.2..ng.g.	0.1705051	0.038275407	0.028560179
M00359_Aminoacyl<>tRNA_biosynthesis_eukaryotes	Valerat...mol.g.	0.170496845	0.038955215	0.028972443
M00001_Glycolysis_[Embden<>Meyerhof_pathway]_glucose_=>_pyruvate	Valerat...mol.g.	-0.170355167	0.039120377	0.029000508
M00077_Chondroitin_sulfate_degradation	Beta.Defensin.2..ng.g.	0.169166909	0.039841097	0.029438895
M00230_Glutamate/aspartate_transport_system	Propionat...mol.g.	0.168524002	0.041308572	0.030424444
M00317_Manganese/iron_transport_system	Calprotectin...g.g.	-0.166962938	0.042536858	0.031228035
M00016_Lysine_biosynthesis_aspartate_=>_lysine	Acetat...mol.g.	-0.166909239	0.043322419	0.031702481
M00050_Guanine_nucleotide_biosynthesis_IMP_=>_GDP/dGDPGTP/dGTP	Butyrat...mol.g.	0.166678315	0.043617015	0.031815759
M00012_Glyoxylate_cycle	Valerat...mol.g.	0.166372832	0.044009299	0.031999343
M00233_Glutamate_transport_system	Acetat...mol.g.	0.166031422	0.044451202	0.03221772
M00144_Complex_I_[NADH_dehydrogenase]_NADH_dehydrogenase_I	Beta.Defensin.2..ng.g.	0.165329826	0.044631445	0.032245665

M00002_Glycolysis_core_module_involving_three<>carbon_compounds	Beta.Defensin.2..ng.g.	0.163958246	0.046456489	0.033458021
M00006_Pentose_phosphate_pathway_oxidative_phase_glucose_6P_=>_ribulose_5P	Beta.Defensin.2..ng.g.	-0.162669976	0.048226893	0.034623499
M00011_Citrate_cycle_second_carbon_oxidation	Butyrat...mol.g.	-0.162566027	0.049149909	0.035175198
M00159_V<>type_ATPase_prokaryotes	Beta.Defensin.2..ng.g.	-0.161572356	0.049779221	0.035513899
M00051_Uridine_monophosphate_biosynthesis_glutamine_[+_PRPP]_=>_UMP	Butyrat...mol.g.	0.16171601	0.050363399	0.035818385
M00232_General_L<>amino_acid_transport_system	Caproat...mol.g.	-0.161420745	0.050790692	0.036009745
M00179_Ribosome_archaea	Calprotectin...g.g.	-0.160123699	0.051891275	0.036562237
M00229_Arginine_transport_system	Calprotectin...g.g.	-0.160161234	0.051835629	0.036562237
M00053_Pyrimidine_deoxyribonuleotide_biosynthesis_CDP/CTP_=>_dCDP/dCTPdTDP/dTTP	Calprotectin...g.g.	-0.1597944	0.052381582	0.036793792
M00219_AI<>2_transport_system	Caproat...mol.g.	0.16022042	0.052558802	0.03680468
M00246_Nickel_transport_system	Caproat...mol.g.	0.15939679	0.053801249	0.037559145
M00198_sn<>Glycerol_3<>phosphate_transport_system	Chromogranin.A..nmol.g.	-0.158657824	0.054103416	0.037654586
M00207_Multiple_sugar_transport_system	Butyrat...mol.g.	-0.157424286	0.056875645	0.039463303
M00335_Sec_[secretion]_system	Chromogranin.A..nmol.g.	-0.156469436	0.057550353	0.039527043
M00184_RNA_polymerase_archaea	Calprotectin...g.g.	-0.15645655	0.057571173	0.039527043
M00193_Putative_spermidine/putrescine_transport_system	Acetat...mol.g.	-0.157249164	0.057155448	0.039527043
M00184_RNA_polymerase_archaea	Propionat...mol.g.	-0.156933768	0.057662234	0.039527043
M00206_Cellobiose_transport_system	Acetat...mol.g.	0.156405624	0.058519142	0.039993983
M00127_Thiamine_biosynthesis_AIR_=>_thiamine<>P/thiamine<>2P	Chromogranin.A..nmol.g.	-0.155421301	0.059264168	0.040141524
M00331_Type_II_general_secretion_system	Calprotectin...g.g.	-0.15547398	0.059177042	0.040141524
M00179_Ribosome_archaea	Propionat...mol.g.	-0.156155884	0.058927974	0.040141524
M00121_Heme_biosynthesis_glutamate_=>_protoheme/siroheme	Beta.Defensin.2..ng.g.	0.154770069	0.06034996	0.040649342
M00001_Glycolysis_[Embden<>Meyerhof_pathway]_glucose_=>_pyruvate	Propionat...mol.g.	-0.15528554	0.060371126	0.040649342
M00016_Lysine_biosynthesis_aspartate_=>_lysine	Beta.Defensin.2..ng.g.	0.154546102	0.060727127	0.040768429
M00360_Aminoacyl<>tRNA_biosynthesis_prokaryotes	Chromogranin.A..nmol.g.	-0.154116391	0.061456189	0.041136529
M00342_Bacterial_proteasome	Chromogranin.A..nmol.g.	-0.153551414	0.062425642	0.041662908
M00317_Manganese/iron_transport_system	Beta.Defensin.2..ng.g.	-0.153250607	0.06294689	0.041887951
M00096_C5_isoprenoid_biosynthesis_non<>mevalonate_pathway	Beta.Defensin.2..ng.g.	0.153035717	0.063321432	0.04201434
M00324_Dipeptide_transport_system	Beta.Defensin.2..ng.g.	-0.152786521	0.06375805	0.042127479
M00009_Citrate_cycle_[TCA_cycle_Krebs_cycle]	Caproat...mol.g.	0.153248615	0.063862163	0.042127479
M00025_Tyrosine_biosynthesis_chorismate_=>_tyrosine	Caproat...mol.g.	0.152999401	0.064300391	0.042293971
M00230_Glutamate/aspartate_transport_system	Valerat...mol.g.	0.152705456	0.064820431	0.04251316
M00088_Ketone_body_biosynthesis_acetyl<>CoA_=>_acetoacetate/3<>hydroxybutyrate/acetone	Acetat...mol.g.	0.15177949	0.066481112	0.04347704
M00051_Uridine_monophosphate_biosynthesis_glutamine_[+_PRPP]_=>_UMP	Calprotectin...g.g.	0.150868229	0.067202097	0.04382262
M00124_Pyridoxal_biosynthesis_erythrose<>4P_=>_pyridoxal<>5P	Chromogranin.A..nmol.g.	-0.150570662	0.067749672	0.043894265
M00028_Ornithine_biosynthesis_glutamate_=>_ornithine	Calprotectin...g.g.	-0.150494408	0.067890577	0.043894265
M00193_Putative_spermidine/putrescine_transport_system	Propionat...mol.g.	-0.151092054	0.067736298	0.043894265
M00119_Pantothenate_biosynthesis_valine/L<>aspartate_=>_pantothenate	Chromogranin.A..nmol.g.	0.15014243	0.068544064	0.04419123
M00219_AI<>2_transport_system	Chromogranin.A..nmol.g.	-0.149888578	0.069018537	0.04437143
M00348_Glutathione_transport_system	Propionat...mol.g.	0.149271098	0.071154609	0.0454877
M00232_General_L<>amino_acid_transport_system	Propionat...mol.g.	0.149347012	0.07100936	0.0454877
M00220_Rhamnose_transport_system	Beta.Defensin.2..ng.g.	-0.148337139	0.07197666	0.045567701
M00019_Leucine_biosynthesis_pyruvate_=>_2<>oxoisovalerate_=>_leucine	Beta.Defensin.2..ng.g.	-0.14818829	0.072265794	0.045567701
M00123_Biotin_biosynthesis_pimeloyl<>CoA_=>_biotin	Beta.Defensin.2..ng.g.	0.148321316	0.072007351	0.045567701
M00200_Sorbitol/mannitol_transport_system	Propionat...mol.g.	-0.14868669	0.07228087	0.045567701
M00028_Ornithine_biosynthesis_glutamate_=>_ornithine	Valerat...mol.g.	0.148993947	0.071686943	0.045567701
M00007_Pentose_phosphate_pathway_non<>oxidative_phase_fructose_6P_=>_ribose_5P	Caproat...mol.g.	-0.148015098	0.073592943	0.046266702
M00260_DNA_polymerase_III_complex_bacteria	Chromogranin.A..nmol.g.	-0.146735798	0.07513688	0.047008291
M00115_NAD_biosynthesis_aspartate_=>_NAD	Propionat...mol.g.	-0.147119715	0.075372139	0.047008291
M00348_Glutathione_transport_system	Caproat...mol.g.	-0.14710972	0.075392194	0.047008291
M00034_Methionine_salvage_pathway	Valerat...mol.g.	0.146957601	0.075697957	0.04706998
M00349_Microcin_C_transport_system	Chromogranin.A..nmol.g.	-0.14629535	0.076025486	0.047144831
M00117_Ubiquinone_biosynthesis_prokaryotes_chorismate_=>_ubiquinone	Valerat...mol.g.	0.144909732	0.079912522	0.049420594
M00336_Twin<>arginine_translocation_[Tat]_system	Chromogranin.A..nmol.g.	-0.143881645	0.081046879	0.049851188
M00144_Complex_I_[NADH_dehydrogenase]_NADH_dehydrogenase_I	Caproat...mol.g.	0.14447122	0.080839076	0.049851188
